# Supplementary material for: Efficacy of exon-skipping therapy for DMD cardiomyopathy with mutations in actin binding domain 1
Source: Mol Ther Nucleic Acids. 2023 Oct 19;34:102060. doi: 10.1016/j.omtn.2023.102060 (PMC10654596; doi:10.1016/j.omtn.2023.102060)
Supplement: Document S1. Figures S1–S10 and Tables S1–S3 [file mmc1.pdf]

OMTN, Volume *34*

Supplemental information

Efficacy of exon-skipping therapy for DMD cardiomyopathy with mutations in actin binding domain 1

Naoko Shiba, Xiao Yang, Mitsuto Sato, Shin Kadota, Yota Suzuki, Masahiro Agata, Kohei Nagamine, Masaki Izumi, Yusuke Honda, Tomoya Koganehira, Hideki Kobayashi, Hajime Ichimura, Shinichiro Chuma, Junichi Nakai, Shugo Tohyama, Keiichi Fukuda, Daigo Miyazaki, Akinori Nakamura, and Yuji Shiba

Supplemental Tables

Table S1. List of primers for genomic PCR and RT-PCR

genomic PCR	Forward primer (5'->3')	Reverse primer (5'->3')
P1 (*AAVS1* intron1)	CTCTTCTCTGTTCAGCCCTAAGAATC	
P2 (*AAVS1* intron1)		CATAGCTCAGTCTGGTCTATCTGCC
P3 (Puro)		CGCGCGTGAGGAAGAGTTCTTG
P4 (*R-CaMP1.07*)	ATGGGTTCTCATCATCATCATCATCATGGTAT GG	
DMD exon1-exon11	GATCACTCACTTTCCCCCTACAG	TGAGGCATTCCCATCTTGAATTTAG
DMD exon1-exon 6	GGCAATTACCTTCGGAGAAAAACG	TTACATTTTTGACCTGCCAGTGGA
Off-target sequencing for *MEF2C-AS1*	AGCTAGGATTTTTAGGAGTGAGCAA	TTGAGAGGGAGTGCTATAAACACAA
Off-target sequencing for *CCIN*	GGTGCTTGGTGAAAGGTTATATCTC	GATTGATGGTGTAGTCCTTTGTCTG
Off-target sequencing for *POLR2K*	CTGGGAATTCAGAGGAATGTCTTCA	GGGAAGCAACTTTACCCTTTATTGT
Off-target sequencing for *NRF1*	TTTGTTATCTGTGCTGAATTTGGGA	GTTCCTGACATACTAATCCATGAATCTT
Off-target sequencing for *LINC00661*	AACGATGACTAGGATGATGAGTGAG	AAACCAAGAGTCTAAAGGCACAAAG
Off-target sequencing for *TOM1L1*	TAGTTATTCTTTGTGTGTCGCACTG	TTAAGGTGTCTTCCATCTCCAAACT
Off-target sequencing for *PARP16*	CAATGTAAAAACTGTGGTAGTGGCT	TAGTCTTTGCTGAGTAAAAGGGGAA
Off-target sequencing for *ENHO/RP11-296L22.8*	CAGGGTAGAGCCATAGTTCCATTTA	CTCACCTAGAGCCTGGAATTAGGAT
Off-target sequencing for *KLHL4-ACA64*	TGAATTGGAGCCTGAACAACTTTAC	TTATATTCATATACCTAAATCTTTTCACTTGCC
Off-target sequencing for *VIT*	CTAATGGTGACACTGGAGGATTTGA	GGAAATCTCAAACTCTTTGGTGAGG
Off-target sequencing for *MAS1*	GAAATACATTTGGCCACCAGTAGAG	TGAGCAAAAATATCAGAGTCCTCCA
Off-target sequencing for *SLAIN2*	ATGTACTAGTGCCCAATCATCAGTT	TGGTTAGAGTAGCGCTGTTGATATT
**RT-PCR**	**Forward primer (5'->3')**	**Reverse primer (5'->3')**
DMD exon2 - exon12	CATTCACAAAATGGGTAAATGCACA	AAGCACCTTATGTTGTTGTACTTGG
**qRT-PCR**	**Forward primer (5'->3')**	**Reverse primer (5'->3')**
TNNT2	TTCACCAAAGATCTGCTCCTCGCT	TTATTACTGGTGTGGAGTGGGTGTGG
MYH6	CTCAAGCTCATGGCCACTCT	GCCTCCTTTGCTTTTACCACT
MYH7	ACAAGCTGCAGCTAAAGGTC	TCAAGATGTGGCAAAGCTAC
TNNI1	CTCTTCAGCAAGAGTTTGCG	CAGCTCCACGAGGACTGAAC
TNNI3	CAGTAGGCAGGAAGGCTCAG	CCTCAAGCAGGTGAAGAAGG
MYL7	GTCTTCCTCACGCTCTTTGG	CCACCTCAGCTGGAGAGAAC
MYL2	TTGGGCGAGTGAACGTGAAAA	CCGAACGTAATCAGCCTTCAG
ATP2A2	CATGACAACCCACTGAGAAGAGAA	CGAAGGTCAGATTGGTCTCATATTT
RYR2	CTGCGCCATTCCTATAGTGG	AGTTGAAGACCGGGAGGTG
CASQ2	AAAGACCCACTCTACGTCGC	CAGGAATTCGTAGCCATCTGGA
CACNA1C	CATGCTCACGGTGTTCCA	TCCTACGGCATCATTGACC
SCN5A	GAGCAACTTGTCGGTGCTG	GATTTGGCCAGCTTGAAGAC
KCNJ2	GGTTTGCTTTGGCTCACTCG	GAACATGTCCTGTTGCTGGC
KCNJ4	TAAACTTGGCCCTGCGTCTT	CTTCTTGACGAAGCGGTTGC
KCND3	TGGCTTCGCGGAAGGGTTT	TGGTGACTCCAGCTCTTGGG
DES	CTGAGCAAAGGGGTTCTGAG	ACTTCATGCTGCTGCTGTGT
SPARC	TTCGGCATCAAGCAGAAGGA	GAAACACGAAGGGGAGGGTT
ALPK3	CTGAGGCCATGCAGAAATGC	ATCTTCCAGTCAACCCCTGC
ANKRD1	TCAAGAACTGTGCTGGGAAG	TAGCTATGCGAGAGGTCTTG
NPPA	TCCAACGCAGACCTGATGGA	GGGCACGACCTCATCTTCTA
NPPB	TGGAAACGTCCGGGTTACAG	CTTCCAGACACCTGTGGGAC
GAPDH	ATGGAAATCCCATCACCATCTT	CGCCCCACTTGATTTTGG

Table S2. DMD sgRNA target sequence

name	region	target sequence		guide sequence + **PAM**	
sgRNA1	intron 2	**ccg** tcatcttcggcagattaatt	rev	AATTAATCTGCCGAAGATGA **CGG**	chrX:32,849,933-32,849,955
sgRNA2	intron 7	**ccc** tatggatggagcatactgca	rev	TGCAGTATGCTCCATCCATA **GGG**	chrX:32,809,222-32,809,244
sgRNA3	intron 9	**cct** cgtgaagagctggtttgttt	rev	AAACAAACCAGCTCTTCACG **AGG**	chrX:32,697,747-32,697,769

PAM; protospacer adjacent motif

Table S3. Potential candidate off-target exonic loci with CFD off-target score more than 0.02.

Off-target sequence is shown with mismatch to sgRNA sequence shown in red.

	**Sequence + PAM**	**CFD Score**		**Associated Gene**
sgRNA1	ACTTAATTTGCTGAAAATGA **AGG**	0.3702	chr5:88943055-88943077	*MEF2C-AS1*
	AATCAATCTGCAGAAGGTGA **AGG**	0.0816	chr9:36170969-36170991	*CCIN*
	AATGTATCTCCCGAAGATAA **GGG**	0.0649	chr8:100153416-100153438	*POLR2K*
	AATTATTCTGCCGTGGCTGA **TGG**	0.0437	chr7:129711550-129711572	*NRF1*
sgRNA2	TGGACTATGCTCCATCCCAA **GGG**	0.0214	chr9:34521211-34521233	*ENHO/RP11-296L22.8*
	CGCAGCAGGCTCCATCCATC **CGG**	0.1299	chr19:16026659-16026681	*LINC00661*
	TGAAGGAGGCTCCATCCACA **TGG**	0.1002	chr15:65258380-65258402	*PARP16*
	AGGAGTATTCTCCATCAATA **TGG**	0.1244	chr17:54915804-54915826	*TOM1L1*
sgRNA3	GGCCAAACCAGCTCTGCACG **TGG**	0.1187	chr2:36808504-36808526	*VIT*
	AAATAACCCACCTCTTCATG **GGG**	0.0692	chr6:159908886-159908908	*MAS1*
	ACACAAACCACCTCCTCACC **TGG**	0.0394	chr4:48420247-48420269	*SLAIN2*

Supplemental Videos

Movie S1. Voltage and calcium imaging of monolayered WT hiPSC-CMs on day 50.

A. Voltage imaging

B. Calcium imaging

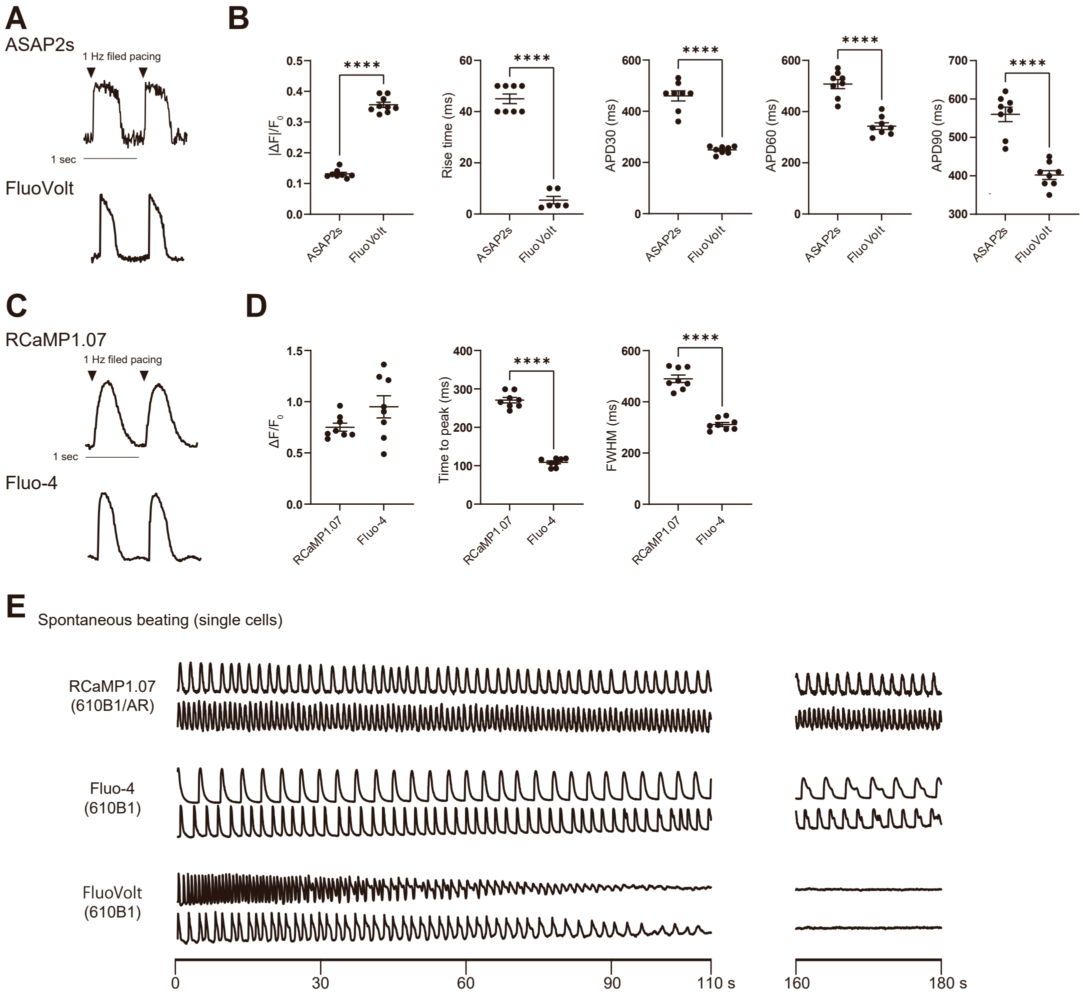

Figure S1. Comparison of live cell imaging waveform between genetically encoded fluorescent ASAP2s/RCaMP1.07 and chemical dyes for voltage and intracellular calcium on day 28 wild-type induced pluripotent stem cell (610B1) derived cardiomyocytes (hiPSC-CMs).

A. Representative traces of ASAP2s and FluoVolt imaging of a single-layered hiPSC-CMs sheet at 1 Hz field pacing. **B.** ΔF/F0, rise time, APD30, APD60, and APD90 (n=8). **C.** Representative traces of RCaMP1.07 and Fluo-4 AM imaging of single-layered hiPSC-CMs sheet at 1 Hz field pacing. **D.** ΔF/F0, Time to peak and full-width at half maximum (FWHM) (n=8). **E.** Representative traces of the single-cell spontaneous beating with RCaMP1.07 on 610B1-ASAP2s/RCaMP1.07 (619B1/AR) cell line and FluoVolt and Fluo-4 on 610B1 cell line for 180-sec traces. In the analysis with FluoVolt and Fluo-4, a decrease in waveform amplitude and the appearance of irregular rhythms were observed after approximately 1 and 2 min, respectively. Abbreviations: fluorescence (F), baseline of fluorescence (F0). Data are presented as mean ± SEM. ****$P < 0.001$.

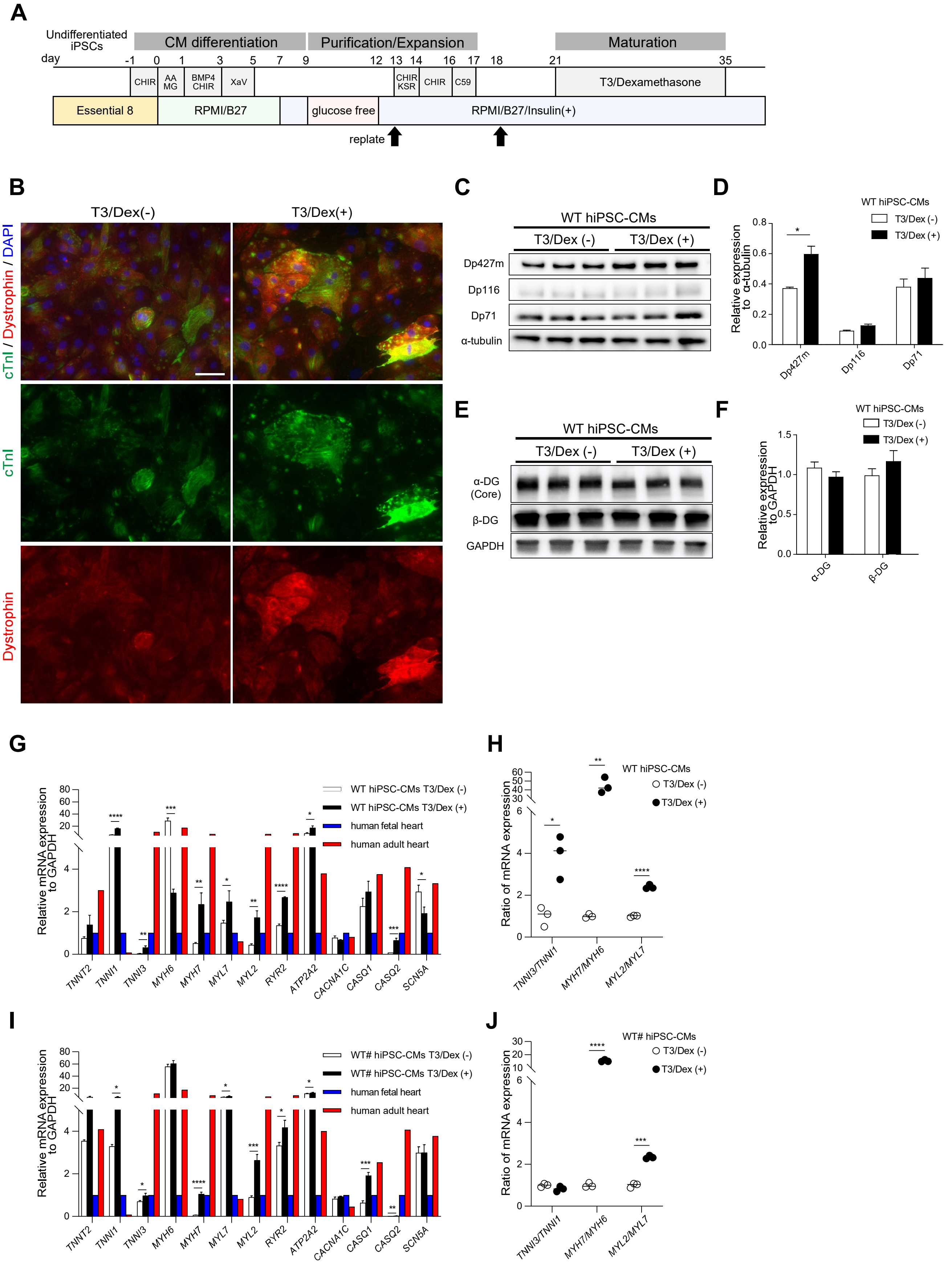
A
Undifferentiated iPSCs
CM differentiation
Purification/Expansion
Maturation
day
-1 0 1 3 5 7 9 12 13 14 16 17 18 21 35
CHIR
AA MG
BMP4 CHIR
XaV
CHIR KSR
CHIR
C59
T3/Dexamethasone
Essential 8
RPMI/B27
glucose free
RPMI/B27/Insulin(+)
replate
B
T3/Dex(-)
T3/Dex(+)
cTnI / Dystrophin / DAPI
cTnI
Dystrophin
C
WT hiPSC-CMs
T3/Dex (-)
T3/Dex (+)
Dp427m
Dp116
Dp71
α-tubulin
D
WT hiPSC-CMs
T3/Dex (-)
T3/Dex (+)
Relative expression to α-tubulin
Dp427m
Dp116
Dp71
E
WT hiPSC-CMs
T3/Dex (-)
T3/Dex (+)
α-DG (Core)
β-DG
GAPDH
F
WT hiPSC-CMs
T3/Dex (-)
T3/Dex (+)
Relative expression to GAPDH
α-DG
β-DG
G
Relative mRNA expression to GAPDH
WT hiPSC-CMs T3/Dex (-)
WT hiPSC-CMs T3/Dex (+)
human fetal heart
human adult heart
TNNT2
TNNI1
TNNI3
MYH6
MYH7
MYL7
MYL2
RYR2
ATP2A2
CACNA1C
CASQ1
CASQ2
SCN5A
H
Ratio of mRNA expression
WT hiPSC-CMs
T3/Dex (-)
T3/Dex (+)
TNNI3/TNNI1
MYH7/MYH6
MYL2/MYL7
I
Relative mRNA expression to GAPDH
WT# hiPSC-CMs T3/Dex (-)
WT# hiPSC-CMs T3/Dex (+)
human fetal heart
human adult heart
TNNT2
TNNI1
TNNI3
MYH6
MYH7
MYL7
MYL2
RYR2
ATP2A2
CACNA1C
CASQ1
CASQ2
SCN5A
J
Ratio of mRNA expression
WT# hiPSC-CMs
T3/Dex (-)
T3/Dex (+)
TNNI3/TNNI1
MYH7/MYH6
MYL2/MYL7

Figure S2. Cardiac differentiation and maturation.

A. Protocol for cardiac differentiation, purification, expansion, and maturation. Abbreviations: AA, activin A; MG, Matrigel; CHIR, CHIR99021; T3, triiodothyronine. **B.** Immunostaining of cardiac troponin I (cTnI) (green) and full-length dystrophin (red) on day 48 isogenic WT control (WT) human induced pluripotent stem cell-derived cardiomyocytes (hiPSC-CMs) with and without T3/Dexamethasone (Dex) treatment. DNA was counterstained with DAPI. Scale bar: 50 µm. **C, D.** Western blot analysis using the anti-dystrophin N-terminus antibody showing protein levels of three isoforms: Dp427, Dp116, and Dp71 in day 48 WT hiPSC-CMs with and without T3/Dex treatment (**C**). Quantification of protein expression in **C** (**D**). **E, F.** Western blot analysis using the anti-human dystroglycan antibody showing protein levels of α-dystroglycan (α-DG (core)) and β-dystroglycan (β-DG) in the same cell lysates as **C** (**E**), and quantification of protein expression in **E** (**F**). **G, I.** Relative mRNA expression of cardiac marker genes to GAPDH in day 48 WT hiPSC-CMs (**G**) and another healthy male-derived non-isogenic control hiPSC-CMs (WT#) (**I**). **H, J.** mRNA ratio of maturation marker genes, TNNI3/TNNI1, MYH7/MYH6, and MYL2/MYL7, in **G** (**H**) and **I** (**J**). Data are presented as mean ± SEM. * $P < 0.05$, ** $P < 0.01$, *** $P < 0.005$, **** $P < 0.001$.

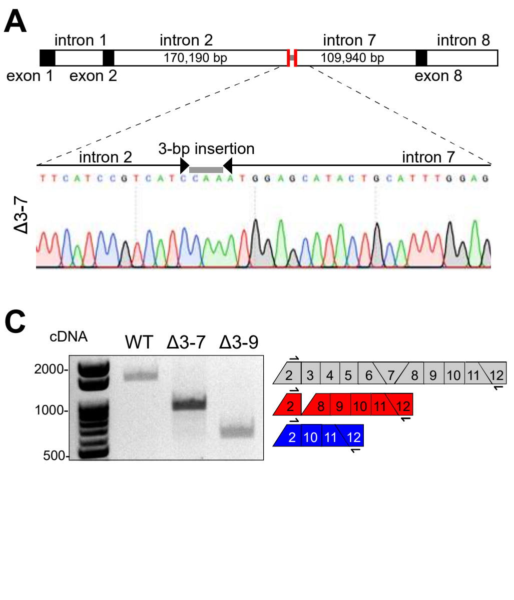

Figure S3. Sequence analysis of isogenic control (WT), Δ3–9, and Δ3–7 human induced pluripotent stem cell-cardiomyocytes (hiPSC-CMs).

A, B. Sanger sequence analyses of genomic DNA extracted from Δ3–7 (**A**) and Δ3–9 (**B**) hiPSC-CMs, including the 5′ side of intron 2 and the 3′ side of the contiguous intron, shows that each cell has a 3-bp and 15-bp insertion in a deep intron close to the protospacer adjacent motif sequences. Red vertical lines indicate cut ends by Cas9. **C.** RT-PCR analysis of CMs differentiated from ASAP2s/R-CaMP1.07-transduced WT, Δ3–7, and Δ3–9 hiPSC-CMs using primers for exons 2 and 12. **D.** Sanger sequence analyses of cDNA from cardiomyocytes differentiated from iCtrl, Δ3–7, and Δ3–9 hiPSC-CMs, including the 3′ side of exon 2 and 5′ side of the subsequent exon, showed that each cell had a correct exon deletion.

A

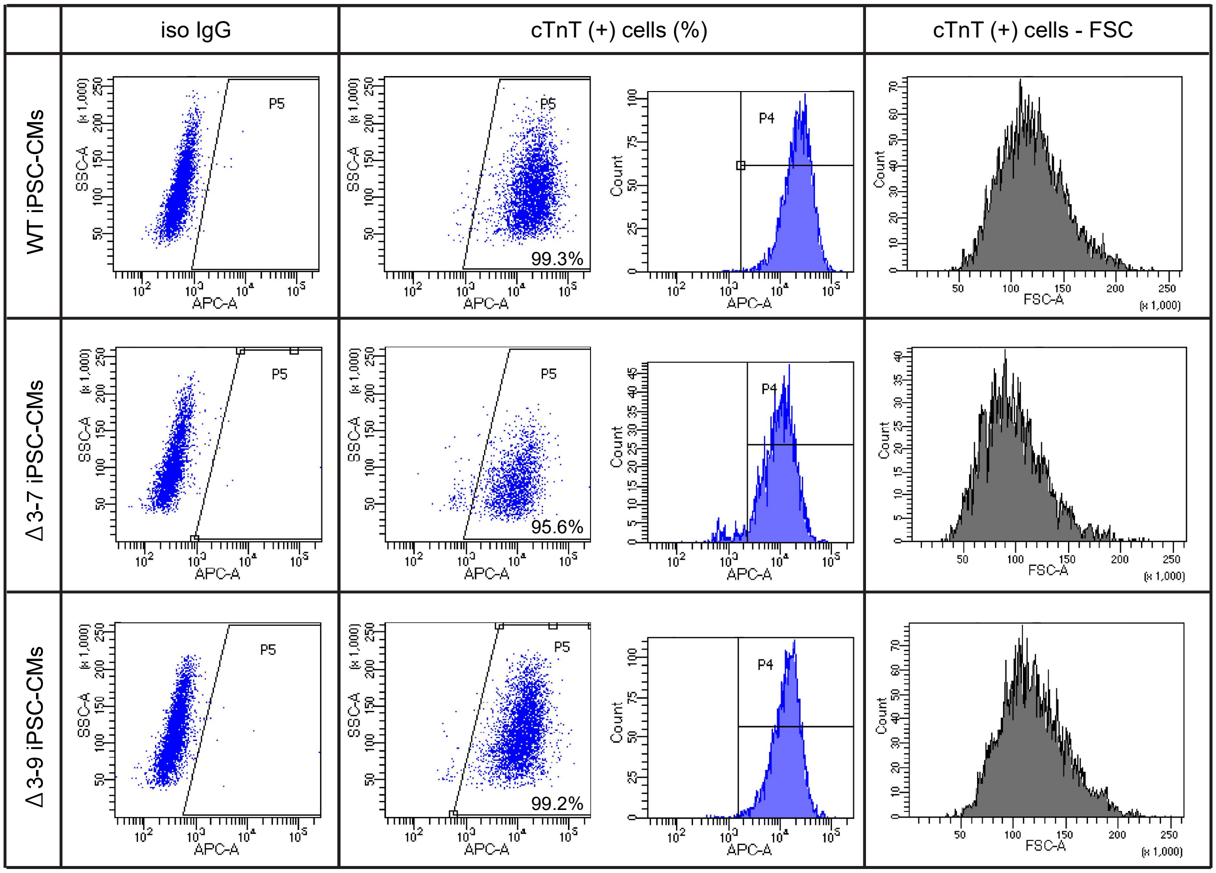

B

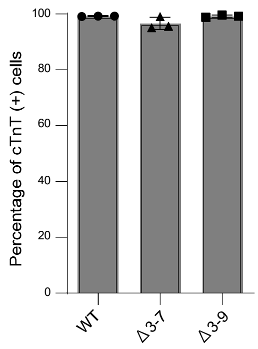

Figure S4. Flow cytometry.

A. Representative flow cytometric analysis at day 48 of human induced pluripotent stem cell-derived cardiomyocytes (hiPSC-CMs) showing the percentage of cardiac troponin T (cTnT) protein expression.

B. Percentage of cTnT positive cells in each cell line, including WT, Δ3-7, and Δ3-9 hiPSC-CMs (n=3).

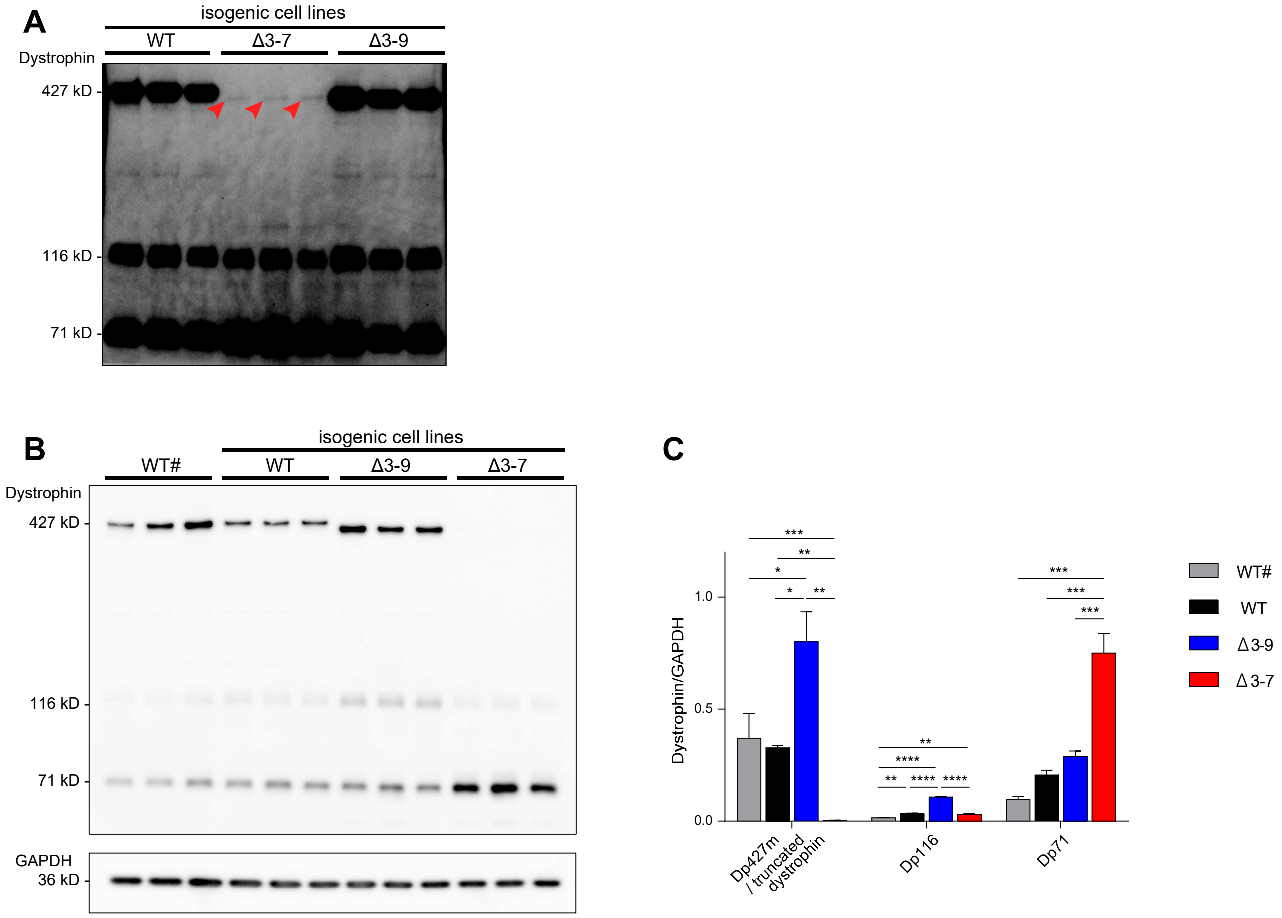

Figure S5. Western blotting analysis.

A. Strongly enhanced membrane picture of dystrophin western blotting using anti-dystrophin N-terminus antibody on Fig. 1F showing faint bands of ~395-kD internally truncated dystrophin on Δ3–7 hiPSC-CMs with the density of approximately 0.5% of WT Dp427m (red arrowhead). B, C. Western blot analysis using the anti-dystrophin N-terminus antibody in day 48 WT hiPSC-CMs, Δ3–7 hiPSC-CMs, Δ3–9 hiPSC-CMs, and another healthy male-derived hiPSC-CMs (WT#) (**B**). Quantification of protein expression in **B** (n=3 independent cardiomyocyte differentiation batches per group) (**C**). Data are presented as mean ± SEM. *$P < 0.05$, **$P < 0.01$, ***$P < 0.005$, ****$P < 0.001$.

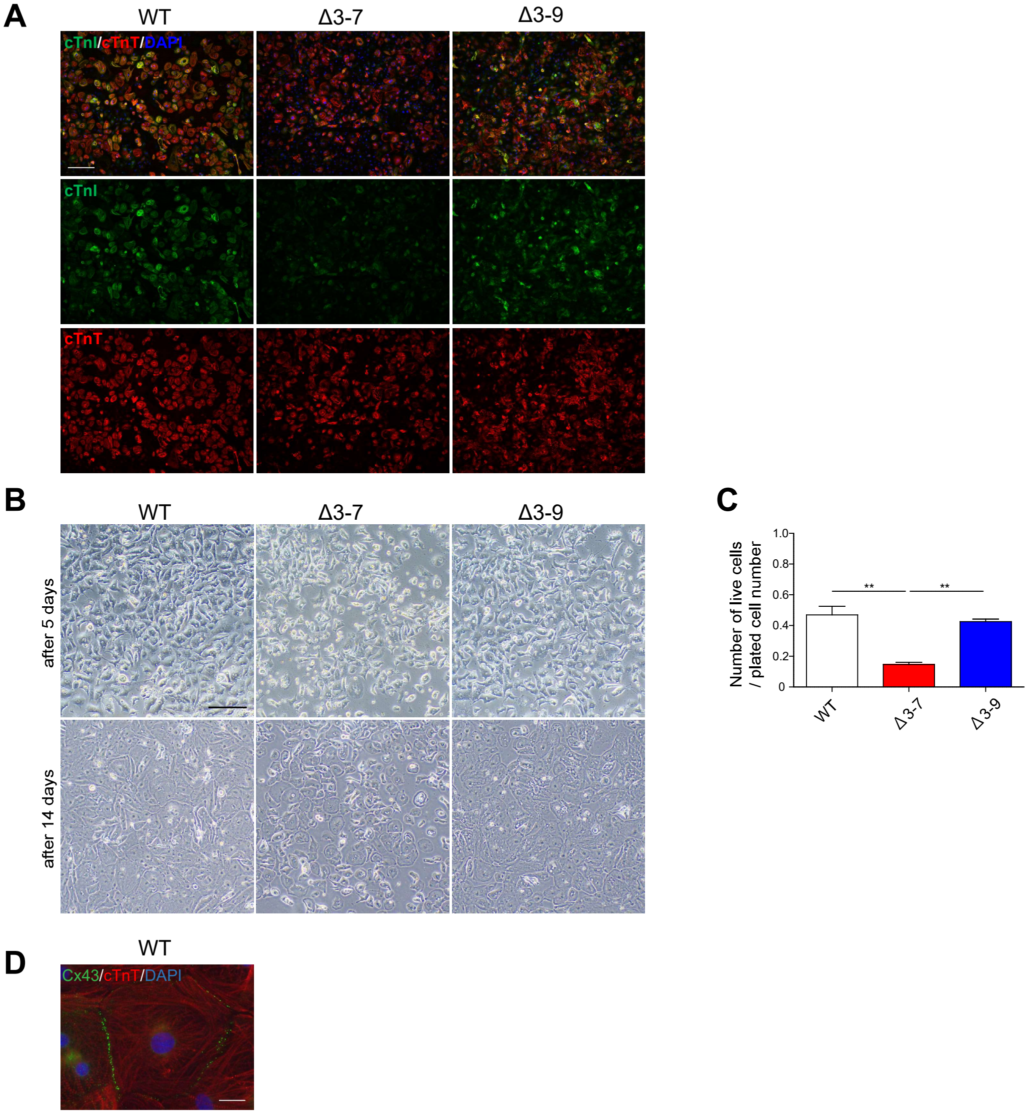

Figure S6. Immunostaining of human induced pluripotent stem cell-cardiomyocytes (hiPSC-CMs).
A. Immunostaining of cardiac troponin I (cTnI) (green) and cTnT (red) on day 48 in WT, Δ3–7, and Δ3–9 hiPSC-CMs. DNA was counterstained with DAPI. Scale bar: 200 µm. **B.** Phase contrast microscopy images of day 35 WT, Δ3–7, and Δ3–9 hiPSC-CMs at 5 d (upper) and 14 d (lower) after replating. Scale bar 500 µm. **C.** Ratio of the number of live cells 14 d after replating to the number of plated cells. **D.** Immunostaining of connexin 43 (Cx43) (green) and cTnT (red) on day 48 in WT hiPSC-CMs. DNA was counterstained with DAPI. Scale bar: 20 µm. Data are presented as mean ± SEM. **$P < 0.01$.

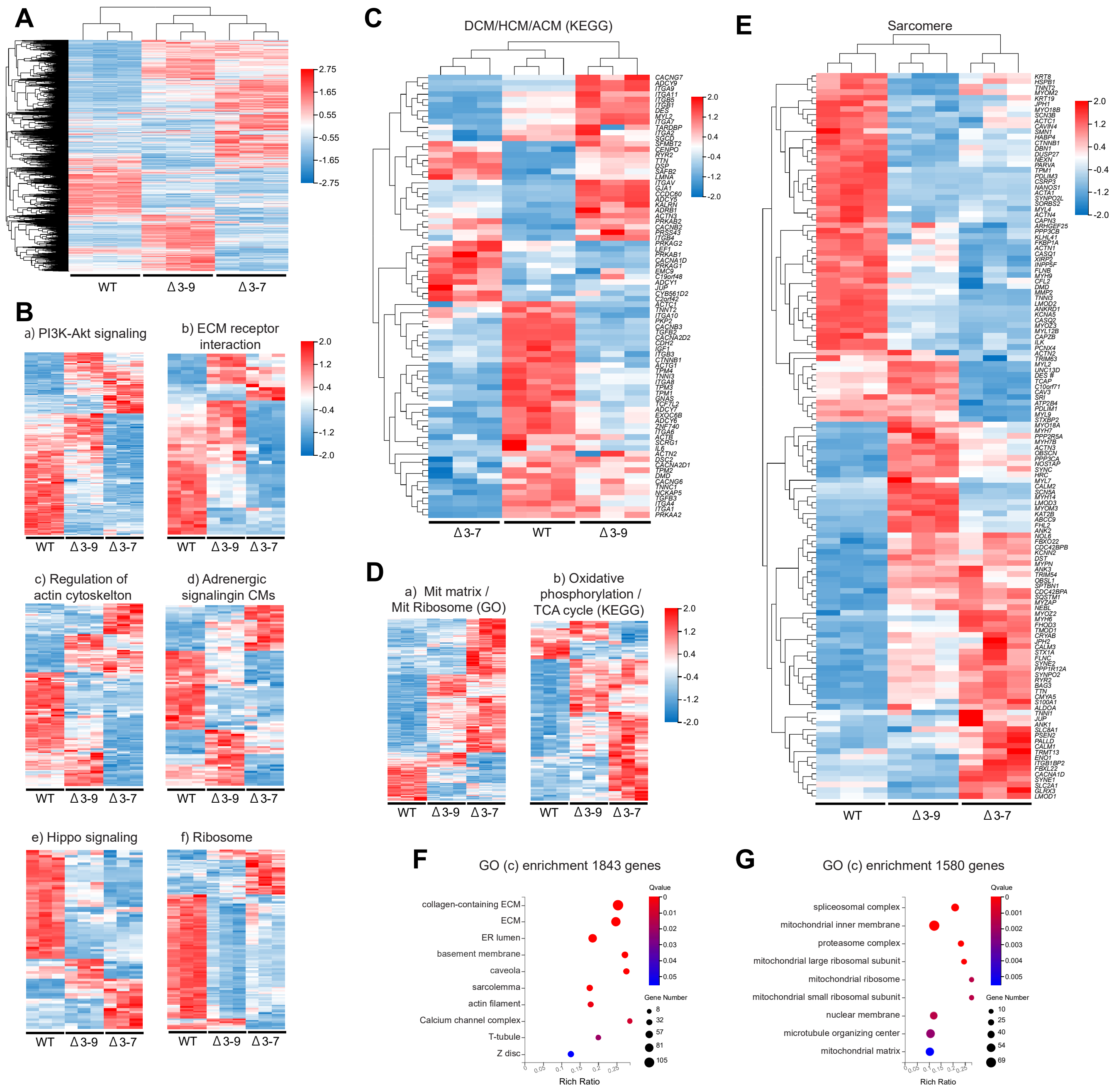

Figure S7. RNA-seq data, related to Figure 5.
A. Hierarchical clustering was performed on 10,000 genes after filtering for low expression levels and coefficients of variation. **B.** Heat map of gene normalized z-scores for log2-transformed FPKM values using the differentially expressed genes (DEGs) involved in major pathways ranked in the Kyoto Encyclopedia of Genes and Genomes pathway enrichment analysis of downregulated genes in Δ3–7 CMs in Fig. 5G, including PI3K-Akt signaling (**a**), ECM receptor interaction (**b**), regulation of actin cytoskeleton (**c**), adrenergic signaling in CMs (**d**), Hippo signaling (**e**), and ribosome (**f**). **C.** Hierarchical clustering of DEGs involved in DCM/HCM/ACM. **D.** Heat map of gene normalized z-scores for log2-transformed FPKM values using the DEGs involved in the mitochondrial matrix and mitochondrial ribosome (**a**) and oxidative phosphorylation and TCA cycle (**b**). **E.** Hierarchical clustering of DEGs involved in the sarcomere, Z-disc, I-band, M-band, and A-band. **F, G.** GO enrichment analysis of the 1843 downregulated (**F**) and 1580 upregulated genes (**G**) in Δ3–7 human induced pluripotent stem cell-cardiomyocytes. Abbreviations: HCM, hypertrophic cardiomyopathy; DCM, dilated cardiomyopathy; ACM, arrhythmogenic cardiomyopathy; CMs, cardiomyocytes; ECM, extracellular matrix.

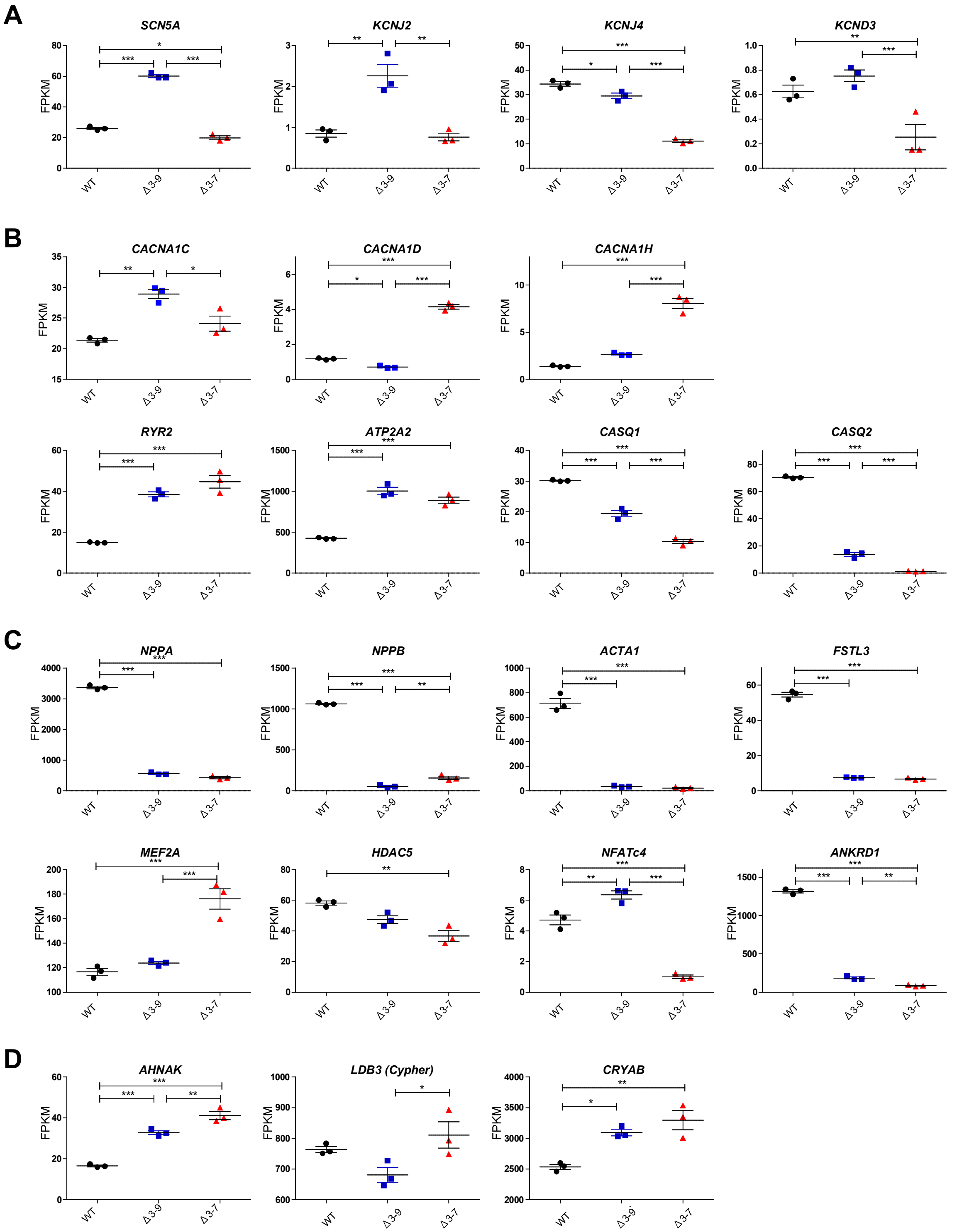

Figure S8. RNA-seq data, related to Figure 5.
A. Action potential associated gene expression (FPKM). **B.** Calcium handling-associated gene expression (FPKM). **C.** Fetal gene and cardiac hypertrophy-related gene expression (FPKM). **D.** Dystrophin-associated cardioprotective gene expression (FPKM). Data are presented as mean ± SEM. *$P < 0.05$, **$P < 0.01$, ***$P < 0.005$.

A

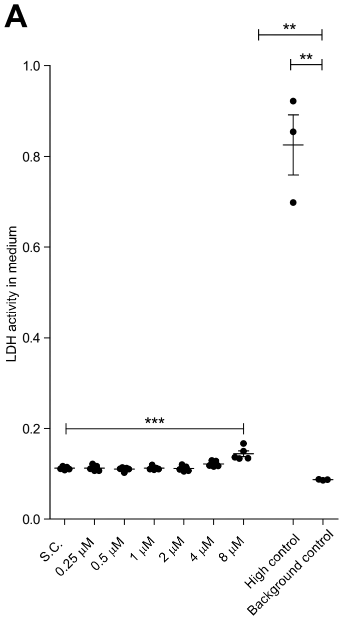

Figure S9. Lactate dehydrogenase (LDH) assay on Δ3–7 human induced pluripotent stem cell-cardiomyocytes (hiPSC-CMs) treated with Vivo-Morpholinos.

A. LDH release level after 12-h incubation with different concentrations of Vivo-Morpholinos (n=5) and Vivo-standard control (S.C.). The high control is the maximum LDH release from the Δ3–7 hiPSC-CMs treated with lysis buffer, and the background control is LDH activity in the culture medium (n=3).

Data are presented as mean ± SEM. $^{**}P < 0.01$, $^{***}P < 0.005$.

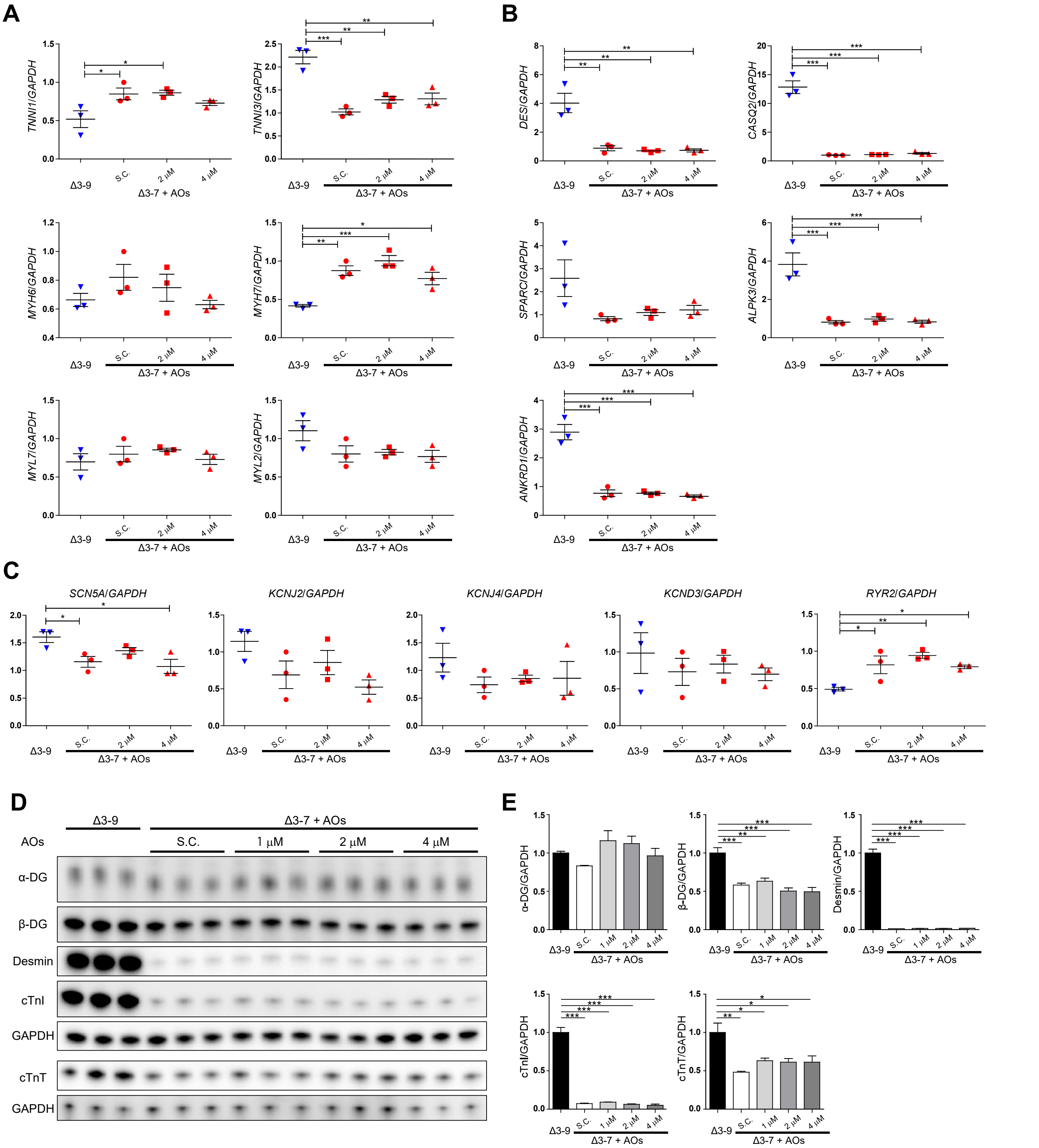

Figure S10. mRNA and protein expression of human induced pluripotent stem cell-cardiomyocytes (hiPSC-CMs) 14 d after exon skipping.

A. RT-PCR analysis of maturation marker genes, including *TNNI3, TNNI1, MYH7, MYH6, MYL2,* and *MYL7*, from untreated Δ3–9 and Δ3–7 hiPSC-CMs 14 d after administration of Vivo-standard control (S.C.) and Vivo-Morpholinos at 1.0–4.0 μM. **B.** RT-PCR analysis of selected genes that were significantly downregulated in day 48 Δ3–7 hiPSC-CMs compared with those in Δ3–9 and wild-type (WT) from untreated Δ3–9 hiPSC-CMs and Δ3–7 hiPSC-CMs 14 d after the administration of S.C. and Vivo-Morpholinos at 1.0–4.0 μM. **C.** RT-PCR analysis of channel genes related to action potential from untreated Δ3–9 hiPSC-CMs and Δ3–7 hiPSC-CMs 14 d after the administration of S.C. and Vivo-Morpholinos at 1.0-4.0 μM. **D.** Western blot analysis showing protein levels of dystrophin-glycoprotein complex composing proteins, including α-DG and β-DG, desmin, cardiac troponin T (cTnT), and cardiac troponin I (cTnI), in Δ 3–9 and Δ3–7 hiPSC-CMs 14 d after administration of S.C. and Vivo-Morpholinos at 1.0–4.0 μM. **E.** Quantification of protein expression in **D**. Data are presented as mean ± SEM. *$P < 0.05$, **$P < 0.01$, ***$P < 0.005$.
