# Supplementary material for: Efficacy of exon-skipping therapy for DMD cardiomyopathy with mutations in actin binding domain 1
Source: Mol Ther Nucleic Acids. 2023 Oct 19;34:102060. doi: 10.1016/j.omtn.2023.102060 (PMC10654596; doi:10.1016/j.omtn.2023.102060)
Supplement: Document S2. Article plus supplemental information [file mmc4.pdf]

Molecular Therapy
Nucleic Acids
Original Article

Efficacy of exon-skipping therapy for DMD cardiomyopathy with mutations in actin binding domain 1

Naoko Shiba,[1,2] Xiao Yang,[1] Mitsuto Sato,[3] Shin Kadota,[1,4] Yota Suzuki,[1] Masahiro Agata,[1] Kohei Nagamine,[1] Masaki Izumi,[1] Yusuke Honda,[1] Tomoya Koganehira,[1] Hideki Kobayashi,[1] Hajime Ichimura,[1] Shinichiro Chuma,[5] Junichi Nakai,[6] Shugo Tohyama,[7] Keiichi Fukuda,[7] Daigo Miyazaki,[3] Akinori Nakamura,[8] and Yuji Shiba[1,4]

[1]Department of Regenerative Science and Medicine, Shinshu University, Matsumoto 390-8621, Japan; [2]Department of Pediatrics, Shinshu University, Matsumoto 390-8621, Japan; [3]Department of Medicine (Neurology and Rheumatology), Shinshu University School of Medicine, Matsumoto 390-8621, Japan; [4]Institute for Biomedical Sciences, Shinshu University, Matsumoto 390-8621, Japan; [5]Department of Regeneration Science and Engineering, Institute for Life and Medical Sciences, Kyoto University, Kyoto 606-8507, Japan; [6]Graduate Schools of Dentistry, Tohoku University, Sendai 980-8575, Japan; [7]Department of Cardiology, Keio University School of Medicine, Tokyo 160-8582, Japan; [8]Department of Clinical Research, National Hospital Organization Matsumoto Medical Center, Matsumoto 399-8701, Japan

Exon-skipping therapy is a promising treatment strategy for Duchenne muscular dystrophy (DMD), which is caused by loss-of-function mutations in the *DMD* gene encoding dystrophin, leading to progressive cardiomyopathy. In-frame deletion of exons 3–9 (Δ3–9), manifesting a very mild clinical phenotype, is a potential targeted reading frame for exon-skipping by targeting actin-binding domain 1 (ABD1); however, the efficacy of this approach for DMD cardiomyopathy remains uncertain. In this study, we compared three isogenic human induced pluripotent stem cell-derived cardiomyocytes (hiPSC-CMs) expressing Δ3–9, frameshifting Δ3–7, or intact *DMD*. RNA sequencing revealed a resemblance in the expression patterns of mechano-transduction-related genes between Δ3–9 and wild-type samples. Furthermore, we observed similar electrophysiological properties between Δ3–9 and wild-type hiPSC-CMs; Δ3–7 hiPSC-CMs showed electrophysiological alterations with accelerated CaMKII activation. Consistently, Δ3–9 hiPSC-CMs expressed substantial internally truncated dystrophin protein, resulting in maintaining F-actin binding and desmin retention. Antisense oligonucleotides targeting exon 8 efficiently induced skipping exons 8–9 to restore functional dystrophin and electrophysiological parameters in Δ3–7 hiPSC-CMs, bringing the cell characteristics closer to those of Δ3–9 hiPSC-CMs. Collectively, exon-skipping targeting ABD1 to convert the reading frame to Δ3–9 may become a promising therapy for DMD cardiomyopathy.

INTRODUCTION

Duchenne muscular dystrophy (DMD) is a type of X-linked lethal muscular dystrophy that affects 1 in 3,600–6,000 live male births.[1,2] This disorder is caused by loss-of-function mutations in the *DMD* gene, which encodes a 427-kDa cytoskeletal protein, dystrophin (Dp427m). *DMD* has 79 exons, consisting of 4 major domains: an N-terminal F-actin binding domain (ABD1) (encoded by exons 1–8), a central rod domain containing a second actin-binding domain (ABD2) (encoded by exons 8–64), a cysteine-rich domain (encoded by exons 64–70), and a C-terminal domain (encoded by exons 71–79).[3,4] Dystrophin is a major component of the dystrophin-associated protein complex (DAPC), which links the actin cytoskeleton to the extracellular matrix (ECM)[5,6] and plays an essential role in stabilizing the sarcolemma, calcium homeostasis, and maintaining muscle cell integrity in skeletal and cardiac muscles. Dystrophin is also reported to play an important role in the regulation of cell division and propagation in satellite cells.[7] Patients with DMD exhibit muscle degeneration and atrophy primarily in skeletal muscle, leading to loss of ambulation at around 10–12 years of age, and gradually develop dilated cardiomyopathy (DCM) and progressive respiratory failure in later stages, usually in the second decade.[4] With improved management for cardiopulmonary dysfunction, especially due to advancements in respiratory support technology, patients with DMD can survive into their forties, although heart failure (HF) remains the leading cause of morbidity and mortality.[4,8,9]

Mutations in *DMD* producing partially functional internally truncated dystrophin protein can cause Becker muscular dystrophy (BMD), a milder form with a later onset and slower progression than DMD, with broad variations in its clinical manifestation that range from asymptomatic to progressive cardiomyopathy leading to early death.[10–12] Single or multiple exonic deletions and duplications

Received 20 December 2022; accepted 17 October 2023;
https://doi.org/10.1016/j.omtn.2023.102060.

Correspondence: Akinori Nakamura, MD, PhD, Department of Clinical Research, National Hospital Organization Matsumoto Medical Center, 2-20-30 Muraimachi-Minami, Matsumoto 399-8701, Japan.
E-mail: anakamu@shinshu-u.ac.jp
Correspondence: Yuji Shiba, MD, PhD, Department of Regenerative Science and Medicine, Institute for Biomedical Sciences, Shinshu University, 3-1-1 Asahi, Matsumoto 390-8621, Japan.
E-mail: yshiba@shinshu-u.ac.jp

This is an open access article under the CC BY license (http://creativecommons.org/licenses/by/4.0/).

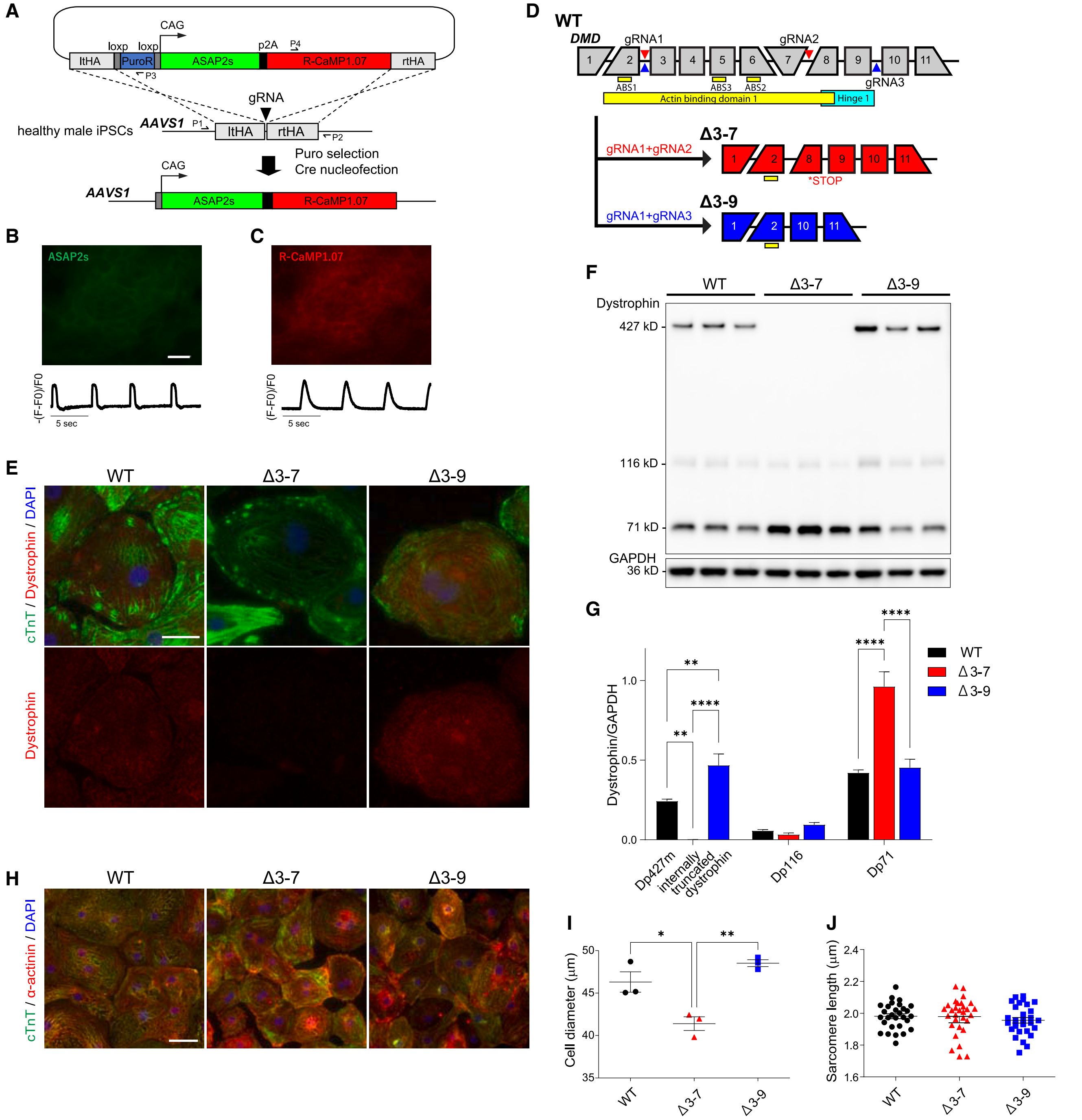

Figure 1. Generation of isogenic cell lines with in-frame and out-of-frame deletions in the ABD1 coding region of *DMD* harboring genetically encoded dual indicator for voltage and calcium in *AAVS1*

(A) An expression cassette of genetically encoded action potential indicator ASAP2s (green) and calcium indicator R-CaMP1.07 (red) was engineered into intron 1 of AAVS1, a safe harbor in a healthy human male hiPSC by CRISPR-Cas9 editing. (B and C) Representative fluorescent images of spontaneously beating hiPSC-CMs (WT, on day 50) showing voltage (ASAP2s) (B, top) and intracellular calcium (R-CaMP1.07) (C, top), and representative traces of voltage (B, bottom) and intracellular calcium ions (C, bottom). Scale bar, 20 μm. (D) Generation of isogenic cell lines with the *DMD* Δ37 and Δ3–9 from WT hiPSCs carrying ASAP2s/RCaMP1.07 (A) by genome editing using gRNAs targeting deep introns. (E) Immunostaining of cTnT (green) and dystrophin (red) in WT, Δ3–7, and Δ3–9 hiPSC-CMs on day 48 after differentiation. DNA was counterstained with DAPI. Scale bar, 20 μm. (F) Western blot analysis showing protein levels of three isoforms of dystrophin protein, Dp427, internally truncated dystrophin, Dp116, and

(legend continued on next page)

account for 80% of the mutations that cause DMD and BMD, and the "reading-frame rule" can explain more than 90% of the mutations in *DMD*.[13] Previous studies on the pathomechanism of dystrophin-associated DCM using patient-derived or genome-edited human induced pluripotent stem cell-derived cardiomyocytes (hiPSC-CMs) recapitulated aberrant cell physiology, including abnormalities in contraction, calcium handling, and mitochondrial dysfunction.[14–21]

Exon skipping therapy to restore the reading frame and produce functional truncated dystrophin using antisense oligonucleotides (AOs), chemically synthesized nucleic acid analogs that specifically bind to a target exon during pre-mRNA, is promising and has already been approved for patients with DMD who have mutations in the most common hotspot in the rod domain in exons 45–55 of *DMD*.[13,22] In exon skipping therapy, the phenotype of the in-frame deletion in *DMD* must be as mild as possible, both in skeletal and cardiac muscles. Therefore, elucidation of the pathogenesis and molecular mechanism underlying the difference in the phenotypes of BMD and DMD is important. One potential candidate region for exon skipping therapy is the minor deletion hotspot, accounting for approximately 7% of patients with DMD, which encompasses exons 3–9 of the *DMD* gene encoding actin-binding domain 1 (ABD1) in the N-terminus of Dp427m.[22,23] This region contains three actin-binding sites (ABSs): ABS1 (encoded by exon 2), ABS2 (encoded by exon 5), and ABS3 (encoded by exon 6). In-frame deletions and missense mutations in ABD1 are commonly associated with a severe form of BMD or DMD, especially in cardiac phenotype, which is attributed to low actin affinity, instability, protein misfolding, and degradation of dystrophin.[24–30] In contrast, patients with the DMD exons 3–9 deletion (Δ3–9), in which ABS1 is spared but ABS2 and ABS3 are absent, were asymptomatic or had a very mild BMD phenotype.[23,31] As such, Δ3–9 has drawn attention as an ideal in-frame deletion goal for exon skipping or gene editing therapy.[15,23,32] AOs targeting exon 8 efficiently induce exon 8 and 9 skipping in myoblasts from patients with DMD with Δ3–7, possibly because of the frequently occurring endogenous skipping of exons 8 and 9.[32] Kyrychenko et al. (2017) demonstrated that the aberrant finding in calcium transient and contraction force was the least in Δ3–9 hiPSC-CMs among isogenic hiPSC-CMs with other in-frame deletions, Δ6–9 and Δ7–11, and frameshifting Δ8–9, and among another isogenic pair of Δ3–7 hiPSC-CMs and Δ3–9 hiPSC-CMs. These findings suggest that the Δ3–9 through genomic editing is an applicable treatment strategy targeting ABD1.

The primary objective of this study was to assess the efficacy of AO-mediated exon 3–9 skipping therapy for ABD1 mutation-induced DMD cardiomyopathy, utilizing a newly developed phonotypic analysis platform for hiPSC-CMs, all the while considering potential future clinical applications. Initially, we were focused on dissecting the pathophysiology of hiPSC-CMs expressing frameshifting Δ3–7 and in-frame Δ3–9, aiming to precisely characterize each phenotype. Notably, this study is the first attempt to conduct a comparative analysis of an isogenic hiPSC-CM set, encompassing a DMD model, a mild BMD model with extended adjacent exonic deletions as a reading frame for exon skipping, and the wild-type (WT). Our findings offer novel insights into the pathology of dystrophin-associated DCM and corroborate the similarities between Δ3–9 hiPSC-CMs and WT hiPSC-CMs, contrasting with those of Δ3–7 hiPSC-CMs. Subsequently, we investigated the potential for converting the DCM pathology observed in Δ3–7 hiPSC-CMs closer to that of Δ3–9 hiPSC-CMs through the partial restoration of Δ3–9 dystrophin via AO administration.

RESULTS

Dp427m was absent and Dp71 was increased in Δ3–7 hiPSC-CMs, whereas internally truncated dystrophin was more abundant in Δ3–9 hiPSC-CMs than Dp427m in WT hiPSC-CMs

To track the action potential and calcium transient in hiPSC-CMs simultaneously, we combined the green fluorescent voltage indicator gene (*ASAP2s*)[33] with the red fluorescent intracellular calcium ion indicator gene (*R-CaMP1.07*)[34] in a plasmid and transduced it into intron 1 of the adeno-associated virus integration site 1 gene (*AAVS1*), a safe harbor in the human genome of healthy male hiPSCs (WT hiPSCs), using the CRISPR-Cas9 gene editing method (Figure 1A). Despite the slower kinetics than those of chemical dye, both indicators worked successfully on differentiated CMs (Figures 1B, 1C, and S1A–S1E; Videos S1A and S1B). Subsequently, we generated two hiPSC lines, the *DMD* Δ3–7 hiPSC line as a model for DMD and the *DMD* Δ3–9 hiPSC line as a model for the mild form of BMD,[23,31] from the WT hiPSCs with dual indicators through electroporation of *Cas9* and two single guide RNAs (sgRNAs) targeting sequences in introns 2 and 7 for Δ3–7 and introns 2 and 9 for Δ3–9 (Figure 1D; Table S2).[15] The sequences, including both the ends of deleted lesions on undifferentiated cell-extracted genomic DNA and mRNA extracted from differentiated CMs of each cell line, were validated (Figures S3A–S3D; Table S1). When compared with WT hiPSC, these cell lines underwent one additional genome editing procedure, which could potentially introduce experimental variability. However, we confirmed the normal sequences of the top candidate exonic regions for off-target effects for the gRNA (Tables S1 and S3). Based on this, we conducted the experiments considering WT, Δ3–7, and Δ3–9 hiPSCs as genetically isogenic cell lines. To obtain a higher purity and more matured hiPSC-CMs suitable for comparative analysis of the disease phenotype, we modified our original cardiac differentiation protocol[35] by adding steps for purification/expansion and maturation with tri-iodothyronine

Dp71 in day 48 WT, Δ3–7, and Δ3–9 hiPSC-CMs. GAPDH was used as a loading control. (G) Quantification of dystrophin isoform protein in F (n = 3 independent CM differentiation batches per group). (H) Immunostaining of cTnT (green) and α-actinin (red) in day 48 control WT, Δ3–7, and Δ3–9 hiPSC-CMs. DNA was counterstained with DAPI. Scale bar, 50 μm. (I) Cell diameter determined by forward scatter in Flow cytometry using the cTnT antibody on WT, Δ3–7, and Δ3–9 hiPSC-CMs on day 48 after differentiation. (J) Sarcomere length of day 48 hiPSC-CMs. Mean length: WT, 1.981 μm; Δ3–9, 1.956 μm; and Δ3–7, 1.979 μm (n = 30). Data are presented as mean ± SEM. *$p < 0.05$, **$p < 0.01$, ****$p < 0.0001$.

(T3)/dexamethasone (Dex) (Figure S2A).[36–39] Although previous studies failed to detect α-DG protein in hiPSC-CMs,[20,40] hiPSC-CMs differentiated using this method showed a sufficiently mature phenotype, with abundant expression of dystrophin, α-dystroglycan (α-DG), and β-dystroglycan (β-DG) on day 48 in WT hiPSC-CMs (Figures S2B–S2F). Furthermore, a major sarcomeric maturation marker of hiPSC-CMs, including the switching in mRNA expression of *TNNI1* coding slow skeletal troponin I (ssTnI) to *TNNI3* coding cardiac TnI (cTnI), *MYH6* coding myosin heavy chain 6 to *MYH7* coding myosin heavy chain 7, and *MYL7* coding myosin light chain 2a (MLC2a) to *MYL2* coding MLC2v, was also promoted following T3/Dex treatment (Figures S2G–S2J) as well as electrophysiological maturation.[37,41]

We verified the loss of Dp427m protein in Δ3–7 hiPSC-CMs on day 48 after differentiation through immunostaining using an antibody against dystrophin exons 31–32 [mandys8] and western blotting using an antibody against the C-terminus of dystrophin (Figures 1E–1G). Notably, the protein level of ∼392-kDa internally truncated dystrophin was significantly higher in Δ3–9 hiPSC-CMs than the level of Dp427m in WT hiPSC-CMs (Figures 1F and 1G). The same results were confirmed when compared with other healthy male-derived hiPSC-CMs (WT#) (Figures S5B and S5C). Almost the same molecular weight of dystrophin was faintly detected in Δ3–7 hiPSC-CMs (Figure S5A), implying endogenous exon skipping associated with a milder phenotype.[32,42] Furthermore, two shorter isoforms of dystrophin with internal promoters, Dp71 and Dp116, were detected in all samples. The protein level of Dp71 in Δ3–7 hiPSC-CMs was more than double that in Δ3–9 and WT hiPSC-CMs, while the level of Dp116 was low in all samples.

Δ3–7 hiPSC-CMs are small, with some aspects of immaturity, representing low adhesion and viability

Next, we analyzed the cell size of hiPSC-CMs through forward scatter in flow cytometry using an anti-cardiac troponin T (cTnT) antibody. Δ3–7 hiPSC-CMs were revealed to be significantly smaller than the WT and Δ3–9 hiPSC-CMs, consistent with previous studies (Figures 1H, 1I, and S4A).[43,44] Regarding maturation, sarcomere length showed no significant difference among the three groups (Figure 1J); however, cTnI protein expression was the lowest in Δ3–7 hiPSC-CMs and the highest in WT hiPSC-CMs (Figures 2A and S6A), which indicated some aspects of maturation defect in the hiPSC-CMs with *DMD* mutations. Furthermore, we found that the number of live cells after replating the cryopreserved hiPSC-CMs on the Matrigel (MG)-coated dish significantly decreased in Δ3–7 hiPSC-CMs compared with that in Δ3–9 and WT hiPSC-CMs (Figures S6B and S6C), indicating impaired cell adhesion and viability in Δ3–7 hiPSC-CMs, which may also reflect proliferative deficiencies.

Desmin intermediate filaments are significantly decreased in Δ3–7 hiPSC-CMs

To evaluate the expression of cytoskeletal actin, DAPC, and major sarcomeric proteins, we performed a western blotting analysis (Figures 2A and 2B). The protein levels of α-actin were comparable among the three groups. In addition, α-DG and β-DG, which play a structural role in tethering dystrophin to the ECM, were decreased in Δ3–7 hiPSC-CMs (Figures 2A and 2B), and the molecular weight of α-DG was lower in Δ3–7 hiPSC-CMs (Figure 2A), which indicates a deficit in glycosylation and is consistent with findings in patients with DMD.[45,46] Notably, desmin was increased 2-fold in Δ3–9 hiPSC-CMs, but decreased 17-fold in Δ3–7 hiPSC-CMs compared with that in WT hiPSC-CMs. cTnT levels were significantly lower in Δ3–7 hiPSC-CMs than Δ3–9 and WT hiPSC-CMs. cTnI levels were lower in both Δ3–9 and Δ3–7 hiPSC-CMs than in WT hiPSC-CMs. As for dystrophin-associated cardioprotective proteins, including AHNAK1, Cypher, and crystallin alpha B (CRYAB), which possibly play roles in maintaining normal cardiac contraction and ion channel functions by interacting with Dp427m in CMs,[20,47–50] there were no significant differences in protein expression among the three groups (Figures 2C and 2D).

Δ3–9 dystrophin represents comparable binding ability to desmin, cTnT, and AHNAK, with about one-half the ability to bind α-actin compared with healthy Dp427m

As actin binding is the principal function of dystrophin in maintaining the stability of cell constriction and the homeostatic balance mediated by several signaling pathways, we investigated the binding ability of internally truncated dystrophin to α-actin in Δ3–9 hiPSC-CMs through co-immunoprecipitation (coIP) using an anti-dystrophin antibody. The signal intensity ratio of α-actin to dystrophin on dystrophin IP samples in western blotting analysis reflects the binding ability of dystrophin to α-actin, revealing that Δ3–9 dystrophin with an incomplete ABD1 (lacking ABS2 and ABS3) could bind to α-actin at the level of 48% of WT Dp427m in hiPSC-CMs. Furthermore, we examined the interaction of Δ3–9 truncated dystrophin with desmin and cTnT and found that Δ3–9 dystrophin could comparably bind them to WT Dp427m (Figures 2E and 2F), implying stabilization by dystrophin binding. Regarding dystrophin-associated cardioprotective proteins, the signal ratio of AHNAK to dystrophin in the dystrophin IP sample in Δ3–9 hiPSC-CMs was comparable with that in WT hiPSC-CMs. However, we could not detect signals against Cypher and CRYAB in the dystrophin IP samples in WT and Δ3–9 hiPSC-CMs, which might be due to their much weaker binding than AHNAK, as previously demonstrated in mouse hearts.[50]

Auto-phosphorylation and oxidation of Ca^{2+}/calmodulin-dependent protein kinase are accelerated in Δ3–7 hiPSC-CMs

Intracellular calcium overload is a promising hypothesis for the pathomechanism of dystrophic features in DMD skeletal and cardiac muscles, and excessive intracellular calcium concentrations have been demonstrated in hiPSC-CMs derived from patients with DMD through live cell imaging using Indo-1.[16] We examined whether Ca^{2+}/calmodulin-dependent protein kinase (CaMKII) is activated in the current hiPSC-CM model for DMD and BMD using Western blot analysis. We found that T287 phosphorylated and M281/M282 oxidized CaMKII were significantly increased, accompanied by increased protein levels of T17 phosphorylated phospholamban (PLB), which is downstream of activated CaMKII in Δ3–7 hiPSC-CMs, compared

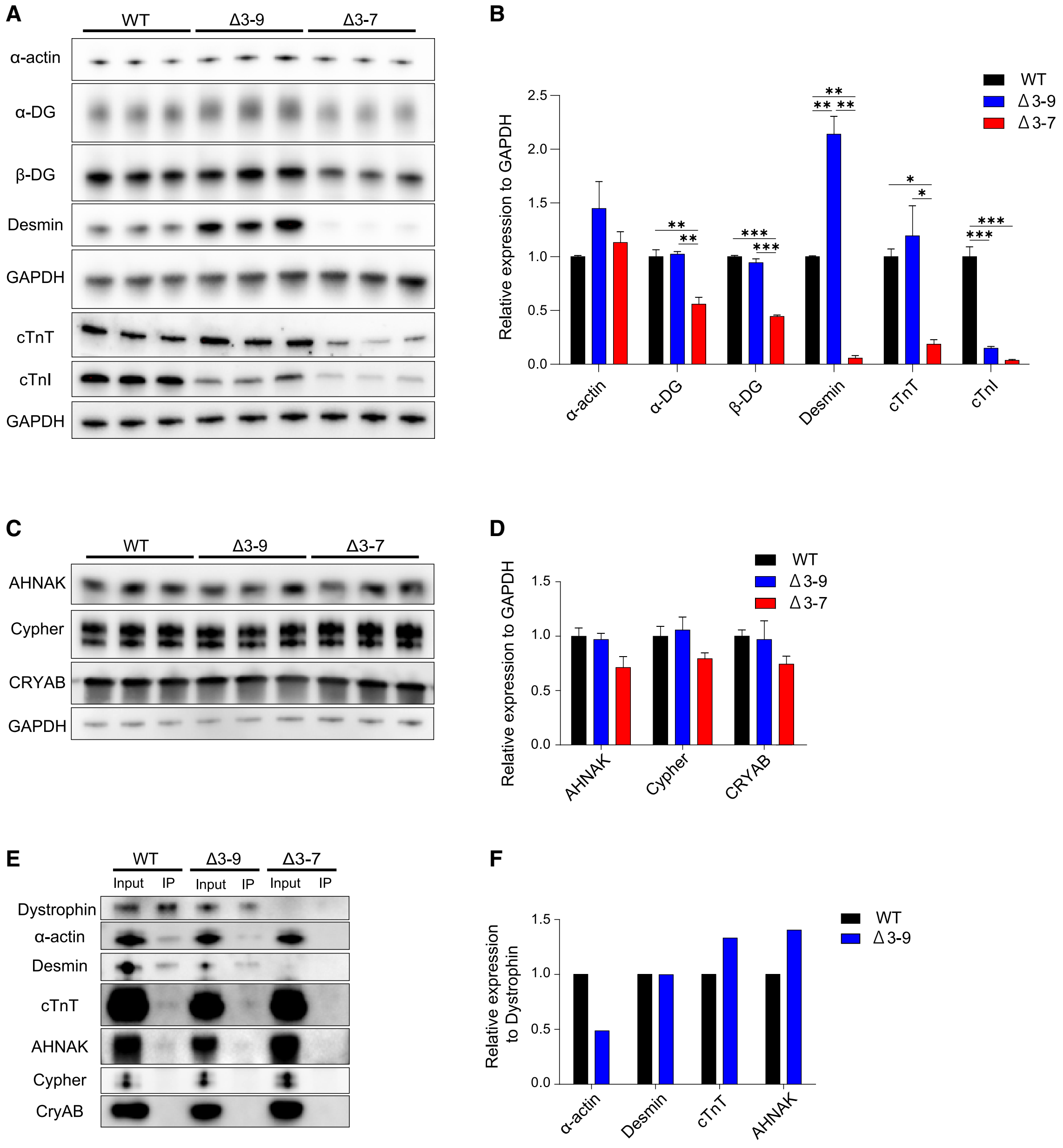

Figure 2. Western blotting analysis

(A) Western blot analysis showing protein levels of dystrophin-glycoprotein complex composing proteins, including α-DG, β-DG, α-actin, desmin, cTnT, and cardiac cTnI, in day 48 WT, Δ3–7, and Δ3–9 hiPSC-CMs. GAPDH was used as a loading control. (B) Quantification of protein expression in (A). (C) Western blot analysis showing protein levels of dystrophin-associated cardioprotective proteins, including AHNAK, Cypher, and CRYAB, in day 48 WT, Δ3–7, and Δ3–9 hiPSC-CMs. GAPDH was used as a loading control. (D) Quantification of protein expression in (C). (E) Western blot analysis of α-actin, desmin, TnT, AHNAK, Cypher, and CRYAB in WT and Δ3–9 hiPSC-CM lysates (input) and dystrophin IP samples. (F) Signal intensity ratio of α-actin, desmin, TnT, AHNAK, Cypher, and CRYAB to Dp427m in dystrophin IP samples in (E). Data are presented as mean ± SEM. *p < 0.05, **p < 0.01, ***p < 0.005.

with Δ3–9 and WT hiPSC-CMs (Figures 3A and 3B). The results indicated that the elevation of intracellular calcium might lead to the activation of other Ca^{2+}-dependent proteases, including protein kinase A, and mitochondrial dysfunction, causing apoptosis or necrosis in Δ3–7 hiPSC-CMs.

Alterations in action potential and calcium transients were detected in Δ3–7 hiPSC-CMs but not in Δ3–9 hiPSC-CMs

We performed electrophysiological analyses on day-50 hiPSC-CMs. As all cells were genetically encoded with fluorescent indicators, we could perform action potential and calcium imaging simultaneously

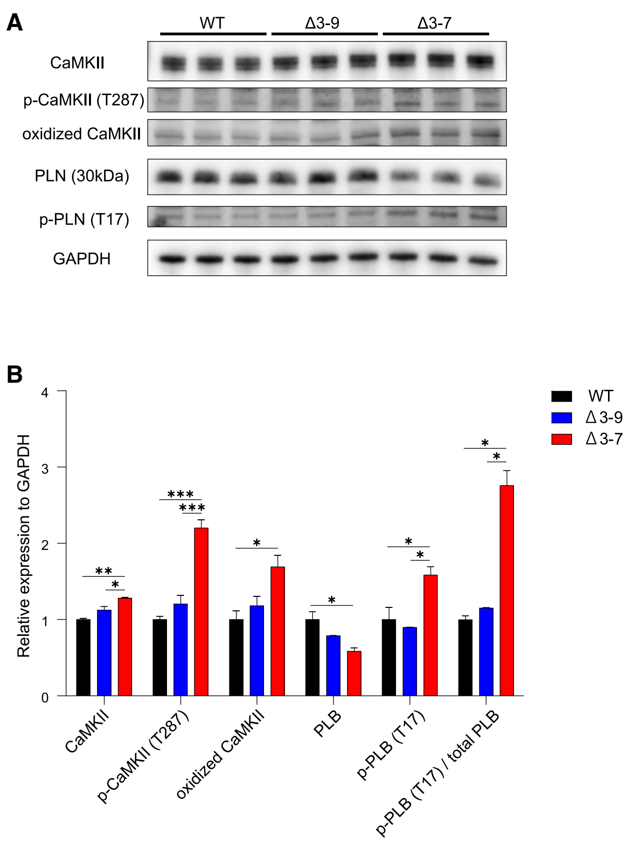

Figure 3. T287 phosphorylation and oxidation of CaMKII; phosphorylation of PLB is elevated in Δ3–7 hiPSC-CMs

(A) Western blot analysis showing that protein levels of phosphorylated CaMKII and oxidized CaMKII were elevated in D3–7 hiPSC-CMs. GAPDH was used as a loading control. (B) Quantification of protein expression of CaMKIIδ, phosphorylated (T287) and oxidized (M281/M282) CaMKII, and phosphorylated PLB (T17). Data are presented as mean ± SEM. *p < 0.05, **p < 0.01, ***p < 0.005.

using a fluorescent confocal microscope without light-sensitive indicator dyes, which could affect the cell condition and contractility (Figures 4A, 4B, and S1E).[51–53] First, we performed single-cell analyses to assess the spontaneous beating rhythm. Spontaneous calcium transient recordings revealed a higher beat rate in Δ3–7 hiPSC-CMs than in the others (Figures 4A–4C), which might correspond with the susceptibility to ventricular tachycardia and sinus tachycardia in patients with DMD.[54,55] No significant arrhythmia, including early after depolarization or delayed after depolarization, was detected in any cell line, which is inconsistent with previous studies,[15,17] while the variability of the inter-beat interval indicating accelerated beat rate variability[56] was augmented in Δ3–7 and Δ3–9 hiPSC-CMs (Figures 4A–4C). Next, we examined monolayered hiPSC-CMs seeded at a higher concentration synchronizing with gap junction formation (Figure S6D). Action potential and calcium imaging in 1/3 Hz-paced hiPSC-CMs showed that the duration of the action potential and both the time to peak and full-width at half maximum (FWHM) in calcium transient were prolonged in Δ3–7 hiPSC-CMs compared with those in Δ3–9 and WT hiPSC-CMs (Figures 4D–4K).

RNA sequencing identified the transcriptomic characteristics of hiPSC-CMs with Δ3–7 and Δ3–9

To identify the transcriptomic characteristics of the hiPSC-CMs differentiated from isogenic *DMD*-mutated hiPSC lines, we performed bulk RNA sequencing (RNA-seq) analysis on Δ3–7, Δ3–9, and WT hiPSC-CMs at 48 d after differentiation. The purity of CMs was 95% or greater in each group, and there was no variation between the groups (Figures S4A and S4B). This provided the expression data for 17,354 genes. Hierarchical clustering of normalized expression levels suggested some similarity between Δ3–7 and Δ3–9 (Figure S7A). However, principal component analysis (PCA) of the whole dataset showed a strong similarity between WT and Δ3–9 hiPSC-CMs, while Δ3–7 hiPSC-CMs seemed to be distinct and separate from them (Figure 5A). The variation observed in PC1 among the samples was inferred to be attributed to the variability in gene expression related to other mesodermal lineages rather than cardiac-related genes according to the Gene Ontology (GO) enrichment analysis of genes that significantly contribute to PC1 and PC2. Volcano plots showed that *NPPA*, *NPPB*, *CASQ2*, *ANKRD1*, *ACTA1*, and *FSTL3* were markedly downregulated in Δ3–7 and Δ3–9 hiPSC-CMs compared with those in WT hiPSC-CMs (Figures 5B, 5C, 5M-a, 5M-b, S8B, and S8C). Among them, *CASQ2* and *ANKRD1* were downregulated in Δ3–7 hiPSC-CMs compared with those in Δ3–9 hiPSC-CMs (Figures 5D, 5M-a, S8B, and S8C). Notably, these genes are associated with cardiac hypertrophy and remodeling.[57,58] In contrast, the expression levels of *DES*, *SPARC*, and *ALPK3* were downregulated in Δ3–7 hiPSC-CMs (Figures 5B and 5D). *DES* encodes desmin intermediate filaments, *SPARC* encodes secreted protein acidic and rich in cysteine (a matricellular protein that functions as a positive inotrope, possibly by interaction with integrin β1 and integrin-linked kinase [ILK] in CMs),[59] and *ALPK3* encodes a transcription factor important in cardiac differentiation and maturation,[60] all of which are causative for cardiomyopathy.

Venn analysis of differentially expressed genes (DEGs) showed that 1,843 genes were significantly downregulated (Figure 5E) and 1,580 genes were upregulated (Figure 5F) in Δ3–7 hiPSC-CMs compared with those in Δ3–9 and WT hiPSC-CMs. To examine the genes involved in the pathology exclusively in Δ3–7 hiPSC-CMs, we performed enrichment analysis on these gene sets. According to a Kyoto Encyclopedia of Genes and Genomes (KEGG) pathway analysis, terms highly enriched in the downregulated gene set included many associated with mechano-transduction (Figure 5G), including the AI3K-Akt signaling pathway (Figure S7B-a) and ECM-receptor interaction (Figure S7B-b), both of which contained several integrin genes and *ILK* (Figures 5M-c), Rap signaling pathway, regulation of actin cytoskeleton (Figure S7B-c), adrenergic signaling in CMs (Figure S7B-d), MAPK signaling pathway, and Hippo signaling pathway (Figure S7B-e). As for our GO enrichment analysis, ECM, sarcolemma, actin filament, and Z-band were included in highly enriched terms, suggesting an alteration in mechano-transduction in Δ3–7

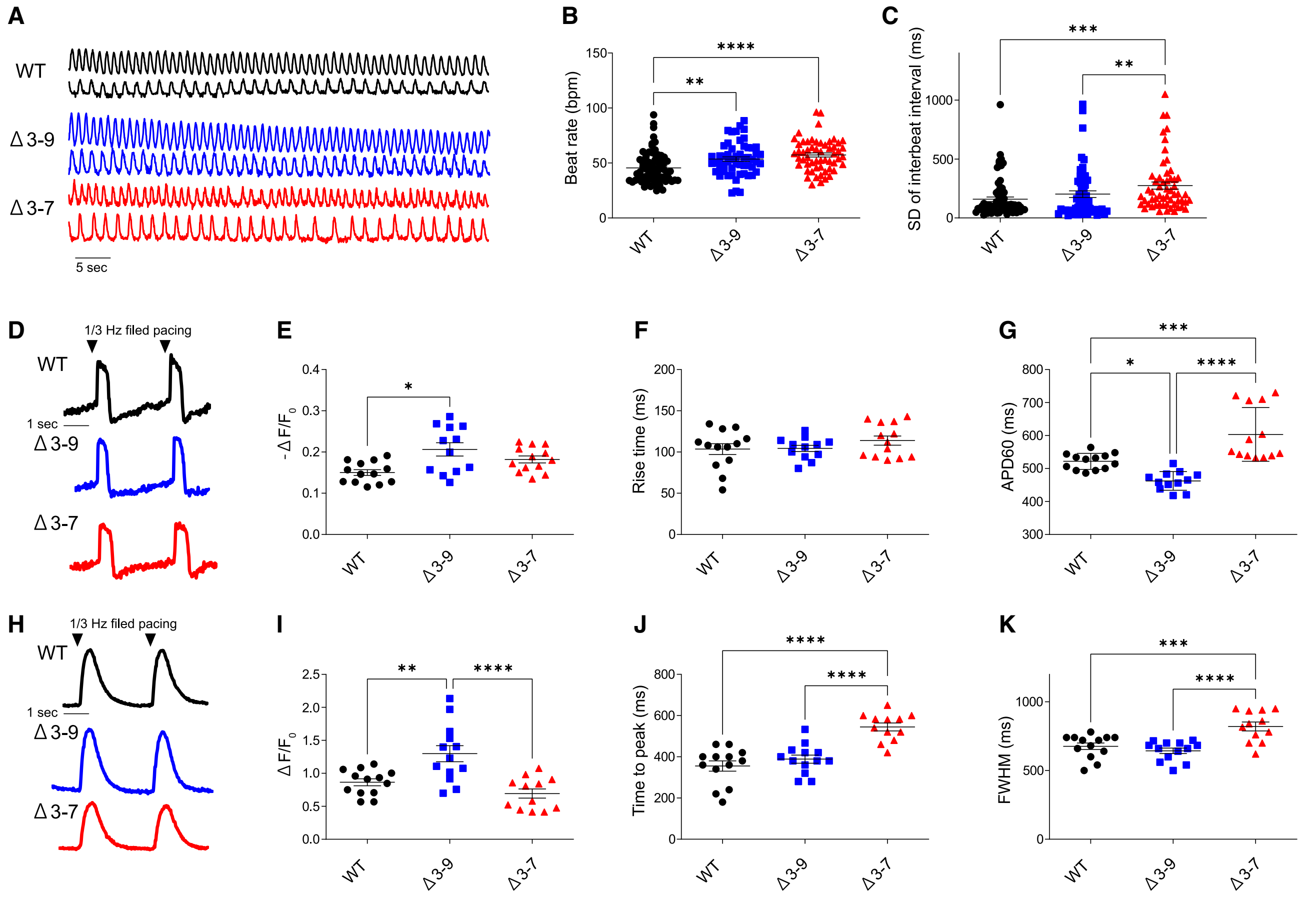

Figure 4. Abnormal action potential and calcium handling in Δ3–7 hiPSC-CMs

(A) Representative traces of the calcium transient of spontaneous beating in WT, Δ3–9, and Δ3–7 hiPSC-CMs on day 55. (B and C) Beating rate (B) and SD of inter-beat intervals (C) of spontaneous beating in single cells (n = 70, 55, 56 for WT, Δ3–9, Δ3–7, respectively). (D–G) ASAP2s imaging of a single-layered hiPSC-CMs sheet at 1/3 Hz field pacing. Representative traces of voltage (D), -(F-F0)/F0 of voltage (E), rise time (F), and APD60 (G) (n = 13, 12, 13 for WT, Δ3–9, Δ3–7, respectively). (H–K) R-CaMP1.07 imaging of a single-layered hiPSC-CMs sheet at 1/3 Hz field pacing. Representative traces of the calcium transient in the single-layered hiPSC-CMs sheet at 1/3 Hz field pacing (H), -(F-F0)/F0 (I), time to peak (J), and FWHM (K) (n = 13, 13, 12 for WT, Δ3–9, Δ3–7, respectively). F/F0, fluorescence (F) normalized to baseline fluorescence (F0). Data are presented as mean ± SEM. *p < 0.05, **p < 0.01, ***p < 0.005, ****p < 0.001.

hiPSC-CMs (Figure S7E). In contrast, no KEGG pathway terms were significantly enriched in the upregulated gene set, indicating that pathways involving downregulated genes have a stronger impact on the pathology of DCM in Δ3–7 hiPSC-CMs than upregulated genes. The GO enrichment analysis of the upregulated gene set showed that several mitochondria-associated terms were significantly enriched (Figure S7F), which is speculated to be a compensatory upregulation, as mitochondrial dysfunction is one of the major pathologies of DCM in DMD.

Next, to determine the pathway involved in cardiac pathology, not only in Δ3–7 hiPSC-CMs, but also in Δ3–9 hiPSC-CMs, we examined the KEGG pathway enrichment of the 1,680 downregulated (Figures 5H and 5I) and 1,646 upregulated (Figures 5J and 5K) genes in both Δ3–7 and Δ3–9 hiPSC-CMs compared with those in WT hiPSC-CMs, with the exclusion of genes showing a mitigating trend in Δ3–9 hiPSC-CMs compared with those in Δ3–7 hiPSC-CMs. As significantly enriched terms in the downregulated genes, ribosome was outstanding (Figures 5I, S7B-e, and S7B-f), while mitochondria-associated terms ranked among the top enriched terms in upregulated genes (Figures 5K, S7D-a, and S7D-b).

Hierarchical clustering of DEGs involved in the sarcomere included numerous genes prominent on the volcano plots (Figures 5B–5D) and overlappingly categorized in DCM, hypertrophic cardiomyopathy, and arrhythmogenic cardiomyopathy (Figures S7C and S7E). Regarding cardiac maturation markers,[41,61,62] the ratios of *TNNI3*/*TNNI1*, *MYH7*/*MYH6*, and *MYL2*/*MYL7* were the lowest in Δ3–7 hiPSC-CMs and *TNNI3*/*TNNI1* and *MYH7*/*MYH6* were the second highest in Δ3–9 hiPSC-CMs among the three groups (Figure 5L). These data indicated a maturation defect in dystrophin-deficient hiPSC-CMs. Regarding the genes involved in calcium handling and

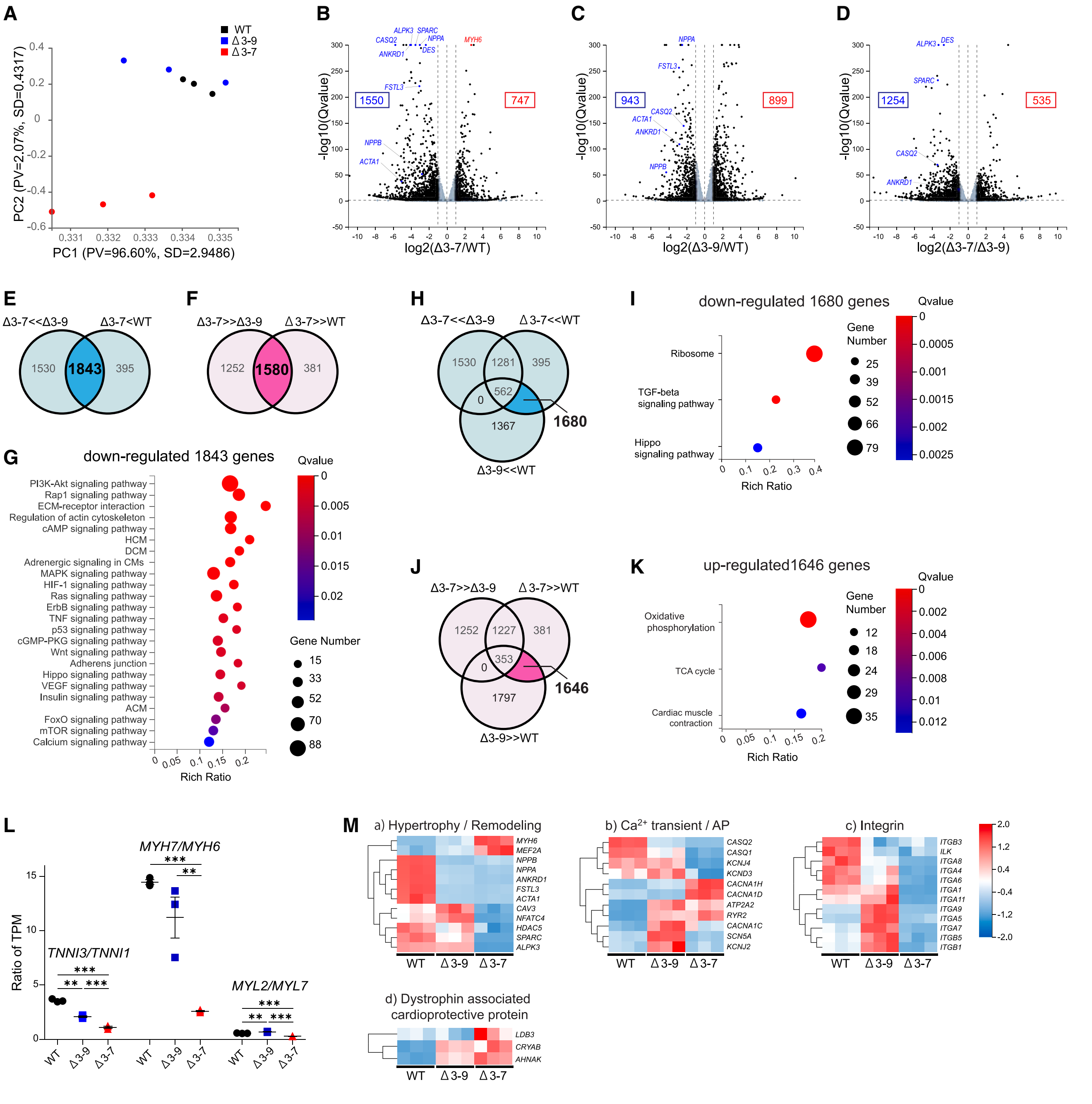

Figure 5. RNA-seq

(A) PCA of RNA-seq performed on day 48 of WT, Δ3–9, and Δ3–7 hiPSC-CMs (n = 3 independent transductions). The percent variance and SD explained by PC1 (96.60%, SD = 2.9486%) and PC2 (2.07%, SD = 0.4317%) are each listed on the respective axes. (B–D) Volcano plot analysis of Δ3–7 hiPSC-CMs versus WT hiPSC-CMs (B), Δ3–9 hiPSC-CMs versus WT hiPSC-CMs (C), and Δ3–7 hiPSC-CMs versus Δ3–9 hiPSC-CMs (D). The x axis represents the fold change in the difference after conversion to log2, and the y axis represents the significance value after conversion to −log10. Gray represents non-DEGs. (E and F) Venn diagram derived from RNA-seq analysis of hiPSC-CMs from three comparisons between WT, Δ3–9, and Δ3–7 hiPSC-CMs. The threshold of DEGs was adjusted to |Log2FC| ≥ 1.0 and false discovery rate-adjusted p ≤ 0.05. In Δ3–7 hiPSC-CMs, 1,843 genes were downregulated (E) and 1,580 genes were upregulated (F) compared with those in WT and Δ3–9 hiPSC-CMs. (G) KEGG pathway enrichment analysis of 1,843 downregulated genes in Δ3–7 hiPSC-CMs. (H–K) Venn diagram derived from RNA-seq analysis of hiPSC-CMs from three comparisons between WT, Δ3–9, and Δ3–7 hiPSC-CMs. In both Δ3–7 and Δ3–9 hiPSC-CMs, 1,680 genes were downregulated compared with WT hiPSC-CMs without downregulation in Δ3–7 hiPSC-CMs compared with Δ3–9 hiPSC-CMs. (H) KEGG pathway enrichment of the 1680 genes extracted from (H). (I) In both Δ3–7 and Δ3–9 hiPSC-CMs, 1,646 genes were upregulated compared with WT hiPSC-CMs without upregulation in Δ3–7 hiPSC-CMs compared with Δ3–9 hiPSC-CMs (J). KEGG pathway enrichment of the

(legend continued on next page)

action potential, *SCN5A*, *KCNJ4*, *KCND3*, *KCNA5*, *CASQ1*, and *CASQ2* were downregulated, and *CACNA1D* and *CACNA1H* were upregulated in Δ3–7 hiPSC-CMs compared with those in Δ3–9 and WT hiPSC-CMs (Figures 5M-b and S8A). *RYR2* and *ATP2A2* were upregulated in both Δ3–7 and Δ3–9 hiPSC-CMs compared with those in WT hiPSC-CMs (Figures 5M-b and S8B). As for the genes encoding dystrophin-associated cardioprotective proteins, *AHNAK* and *CRYAB* were significantly upregulated in both Δ3–7 and Δ3–9 hiPSC-CMs compared with those in WT hiPSC-CMs, and *LDB3* encoding Cypher was upregulated in Δ3–7 hiPSC-CMs compared with that in Δ3–9 hiPSC-CMs, implying a compensatory increase in the loss or aberrant interaction with dystrophin (Figures 5M–5D and S8D). These molecules may be involved in the pathogenesis of DMD cardiomyopathy; however, there has not been a definitive consensus on their expression and function in DMD CMs because there have been limited studies conducted so far.[20] Thus, further study is needed to gain a better understanding.

AOs targeting exon 8 converted the reading frame to Δ3–9, restoring functional dystrophin with reduced Dp71 isoform and improving electrophysiology

To explore whether exon skipping induced by AOs changes the phenotype of Δ3–7 hiPSC-CMs closer to Δ3–9 hiPSC-CMs, we performed exon skipping using Vivo-Morpholinos targeting exon 8 in Δ3–7 hiPSC-CMs, with the concentration range of 0.5–4 μM in a growth medium, which was confirmed to be nontoxic to hiPSC-CMs through a lactate dehydrogenase (LDH) cytotoxic assay (Figure S9). We performed RT-PCR and western blotting on cDNA and whole-protein samples extracted from hiPSC-CMs 14 d after treatment. RT-PCR analysis revealed three skipping patterns by the combination of exon 8 skipping induced by AOs and endogenous exon 9 skipping. The frequency of exon 8+9 skipping increased in a dose-dependent manner (Figure 6A), whereas the frequency of single exon 8 skipping was very low. This is consistent with an experiment on myoblasts derived from patients with DMD using 2′-O-methyl-modified bases on a phosphorothioate backbone.[32] Western blotting analysis revealed that internally truncated dystrophin was detected in AO-treated Δ3–7 hiPSC-CMs and increased in a dose-response manner, reaching 43% of dystrophin protein levels in Δ3–9 hiPSC-CMs at 4 μM AOs. Dp71 was partially decreased by AO treatment in a dose-dependent manner, with a 33% decrease at 4 μM (Figures 6B and 6C). Immunostaining also showed dystrophin restoration, albeit faintly, in Δ3–7 hiPSC-CMs (Figure 6D).

Next, we performed an electrophysiological analysis on hiPSC-CMs 20 d after AO administration. The single-cell analysis showed no significant arrhythmic cells in any of the samples, and the variability of the inter-beat interval tended to be reduced in Δ3–7 hiPSC-CMs, although the difference was not significant (Figure 7A). The monolayered analysis of the action potential with field pacing showed that the APD60 was shortened by the restoration of truncated dystrophin at ∼43% level of Δ3–9 hiPSC-CMs at 4 μM of AOs ($p < 0.005$) (Figure 7B). As for the calcium transient, the time to peak was shortened at 1 μM ($p < 0.05$), with the restoration of trace amounts of dystrophin and 4 μM with a greater effect ($p < 0.001$) (Figure 7D), and the FWHM was shortened at 2 μM and a higher dose ($p < 0.005$) in Δ3–7 hiPSC-CMs (Figure 7E). In summary, in the calcium transient, the rising phase was accelerated by a small restoration of Δ3–9 truncated dystrophin, and the duration of calcium transient and action potential was shortened by more restoration of dystrophin in Δ3–7 hiPSC-CMs.

To further investigate the early effect of exon skipping, we performed RT-PCR and western blotting analyses, focusing on the molecules indicated to be affected in Δ3–7 hiPSC-CMs by the above experiments. Notably, the ratio of *TNNI3/TNNI1* increased after treatment with 4 μM in Δ3–7 hiPSC-CMs (Figures 6E and S10A). *DES*, *CASQ2*, *SPARC*, *ALPK3*, and *ANKRD1* maintained low expression in Δ3–7 hiPSC-CMs for at least 2 weeks after treatment (Figure S10B). Channel genes, including *SCN5A*, *KCNJ2*, *KCNJ4*, *KCND3*, and *RYR2*, showed no significant changes (Figure S10C). The protein levels of α-DG, β-DG, desmin, cTnI, and cTnT and glycosylation of α-DG were not restored by the treatment (Figures S10D and S10E).

Next, we examined whether CaMKII activation was mitigated via exon skipping. Western blotting analysis showed that the protein level of T287 phosphorylated CaMKII was significantly decreased in Δ3–7 hiPSC-CMs by the administration of AOs at 4 μM (Figures 8A–8C), while the protein level of oxidized CaMKII was not changed by the treatment (data not shown).

DISCUSSION

This study investigated the validity and efficacy of ABD1 targeting exon skipping treatment to correct the reading frame to Δ3–9 using three isogenic hiPSC-CMs, including two models of dystrophinopathy: the DMD model caused by Δ3–7 and a very mild version of the BMD model caused by Δ3–9, along with the WT control.

In Δ3–7 hiPSC-CMs, Dp427m protein was absent, as expected. The protein level of internally truncated dystrophin was approximately 2-fold higher in Δ3–9 hiPSC-CMs than in WT hiPSC-CMs, implying a compensatory increase for the impaired function of shortened dystrophin or high tertiary structure stability; this may be partially attributable to the increased transcription rate resulting from array shortening. Following dystrophin coIP, we estimated that the truncated dystrophin lacking ABS2 and ABS3 can bind α-actin at the level of

1646 genes extracted from (J) (K). (L) FKPM ratio of maturation marker genes, including *TNNI3/TNNI1*, *MYH7/MYH6*, and *MYL2/MYL7*, from WT, Δ3–9, and Δ3–7 hiPSC-CMs. (M) Heatmap of gene normalized z-scores for log2-transformed transcripts per kilobase of exon model per million mapped read values using DEGs involved in hypertrophy and remodeling (a), Ca^{2+} transient and action potential (AP) (b), integrin (c), and dystrophin-associated cardioprotective protein (d). Data are presented as mean ± SEM. $^{**}p < 0.01$, $^{***}p < 0.005$.

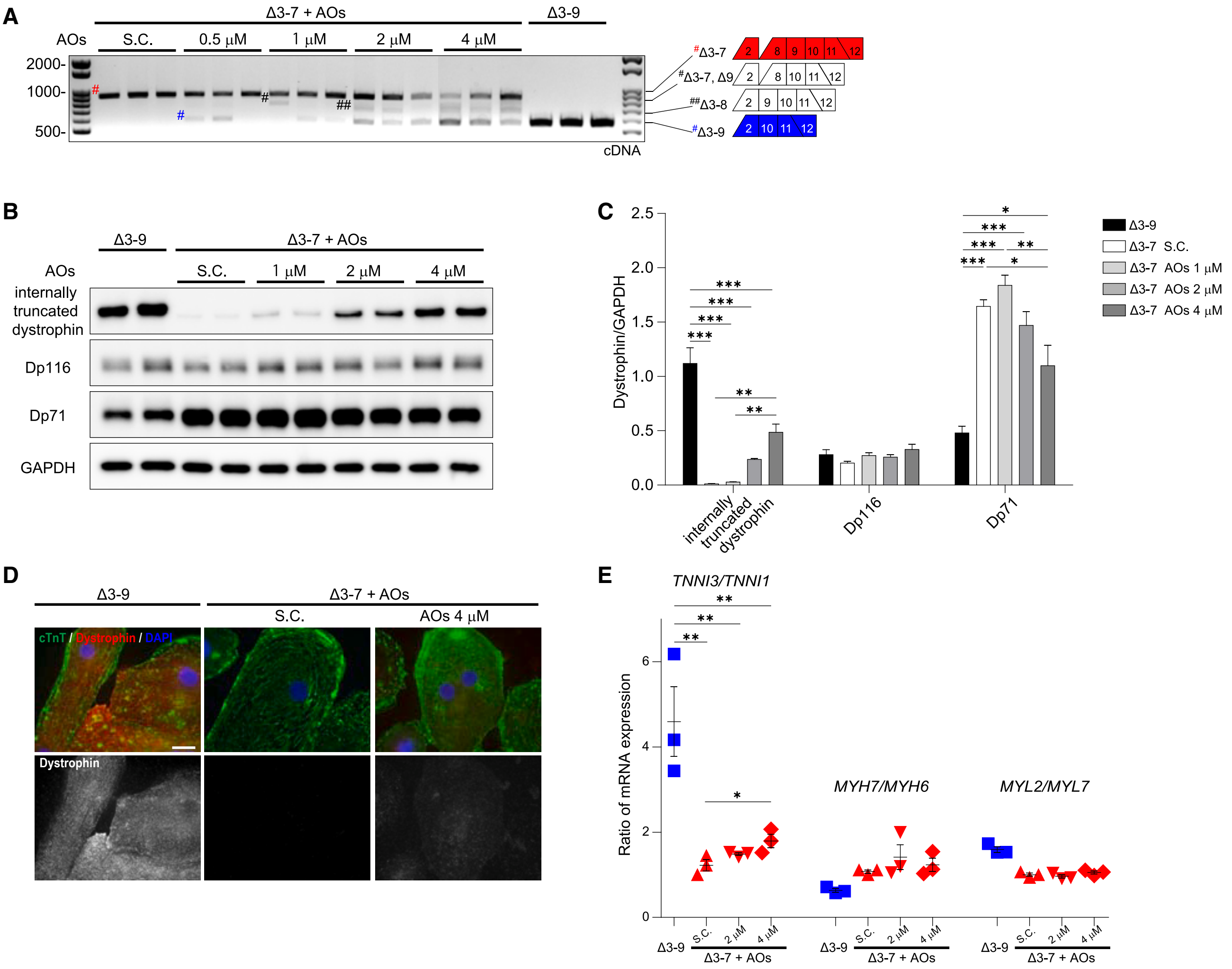

Figure 6. Exons 8–9 skipping restores dystrophin expression in Δ3–7 hiPSC-CMs

(A) RT-PCR analysis of dystrophin transcripts using primers on exons 2 and 12 from Δ3–7 hiPSC-CMs 14 d after treatment with Vivo-S.C. and Vivo-Morpholinos targeting exon 8 at 0.5–4 μM and untreated Δ3–9 hiPSC-CMs. Red octothorpe, Δ3–7; black octothorpe, Δ3–7 and Δ9; black double octothorpes, Δ3–8; blue octothorpe, Δ3–9. (B) Representative Western blot image showing dystrophin expression in untreated Δ3–9 and Δ3–7 hiPSC-CMs 14 d after administration of S.C. and Vivo-Morpholinos at 1.0–4.0 μM. GAPDH was used as a loading control. (C) Quantification of dystrophin internally truncated dystrophin, Dp116, and Dp71 proteins in (B). (D) Immunostaining of cTnT (green) and dystrophin (red) on untreated Δ3–9 hiPSC-CMs and Δ3–7 hiPSC-CMs treated with S.C. and 4 μM Vivo-Morpholino. DNA was counterstained with DAPI. Scale bar, 20 μm. (E) RT-PCR analysis of maturation marker gene ratios, including *TNNI3/TNNI1*, *MYH7/MYH6*, and *MYL2/MYL7*, from untreated Δ3–9 hiPSC-CMs and Δ3–7 hiPSC-CMs 14 d after administration of S.C. and Vivo-Morpholinos at 2.0–4.0 μM. Data are presented as mean ± SEM. *$p < 0.05$, **$p < 0.01$, ***$p < 0.005$.

48% of WT Dp427m. These results suggest that the dystrophin binding rate of α-actin in Δ3–9 hiPSC-CMs is approximately 90% of that in WT hiPSC-CMs, which might corroborate the reduced severity of phenotypes in BMD caused by Δ3–9. Notably, the protein level of the dystrophin shorter isoform Dp71 in Δ3–7 hiPSC-CMs was more than 2-fold higher than that in Δ3–9 hiPSC-CMs and WT hiPSC-CMs (Figures 1E–1G). In addition, Dp71 expression was decreased by AO treatment following the restoration of internally truncated dystrophin in a dose-dependent manner in a short period, which is a new discovery. Dp427m is a full-length dystrophin located in the sarcolemma and T-tubules in skeletal and cardiac muscles and is responsible for the pathogenesis of DMD and BMD. Dp71 is a ubiquitously expressed isoform,[63] and Dp116 is a Schwann cell-specific isoform that is also expressed in the human heart.[64] Although the function of these isoforms in the cardiac muscle remains to be elucidated, previous studies have implicated the detrimental effects of Dp71 in dystrophin-associated DCM pathogenesis.[65–67] Genotype-phenotype analyses of patients with DMD have suggested that the absence of Dp71 or Dp116 might be associated with less severe cardiac symptoms.[64] The mechanism and pathophysiology of Dp71 upregulation in Δ3–7 hiPSC-CMs were not investigated in this study, and the expression of Dp71 in DMD hiPSC-CMs caused by other

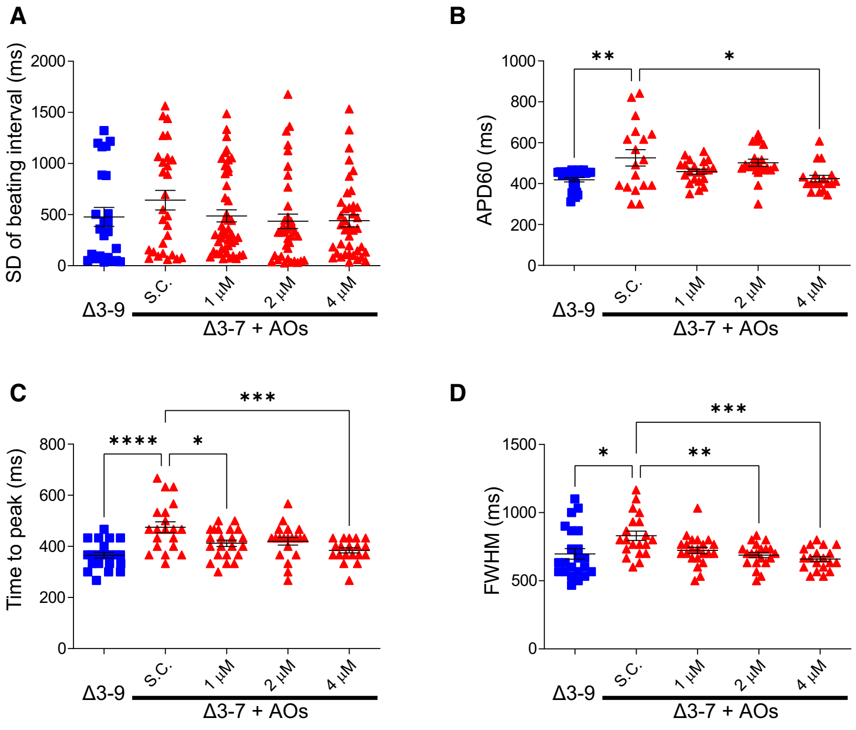

Figure 7. Action potential and calcium handling after exon skipping by Vivo-Morpholinos

(A) SD of the beating interval of spontaneous beating in single-cell analysis (n = 27 and 31, 47, 39, 42 for Δ3–9 hiPSC-CMs and Vivo-S.C. and Vivo-Morpholino-treated Δ3–7 hiPSC-CMs at 1, 2, 4 μM, respectively). (B) APD60 of action potential in monolayered cells at 1/3 Hz pacing (n = 24 and 18, 21, 21, 18 for Δ3–9 hiPSC-CMs and S.C and Vivo-Morpholino-treated Δ3–7 hiPSC-CMs at 1, 2, 4 μM, respectively). (C and D) Time to peak (C) and FWHM (D) of calcium transient in monolayered cells at 1/3 Hz pacing (n = 22 and 20, 22, 20, 19 for Δ3–9 hiPSC-CMs and S.C and Vivo-Morpholino-treated Δ3–7 hiPSC-CMs at 1, 2, 4 μM, respectively). Data are presented as mean ± SEM. *$p < 0.05$, **$p < 0.01$, ***$p < 0.005$, ****$p < 0.001$.

mutations has not been studied so far; however, increased Dp71 might have an aggravating effect on DCM in patients with DMD.

Desmin being significantly reduced in Δ3–7 hiPSC-CMs accompanied by reduced sarcomeric proteins, including cTnT and cTnI, was also a novel insight of this study. Desmin is a major intermediate filament of striated and smooth muscles, which plays an important role in the maintenance of muscle structural and cellular integrity, force transmission, and mitochondrial homeostasis by linking the contractile myofibrils to the sarcolemma and cellular organelles, constructing a network with various muscle component proteins, including dystrophin and dystrophin-associated proteins.[68,69] Mutations in *DES* cause cardiomyopathy and skeletal myopathy, with prominent mitochondrial dysfunction.[70,71] In the skeletal muscles of *mdx* mice, desmin expression is increased and plays a beneficial role in dystrophic muscles.[72] However, the expression and role of desmin in cardiac muscle have not been studied in patients with DMD or animal or cell models. Our study revealed highly reduced desmin expression at both the mRNA and protein levels in Δ3–7 hiPSC-CMs, whereas desmin protein was 2-fold higher in Δ3–9 hiPSC-CMs than in WT hiPSC-CMs. Furthermore, the binding ability of Δ3–9 truncated dystrophin to desmin was comparable with that of WT Dp427m, probably owing to the spared desmin-binding region in the cysteine-rich domain of dystrophin in Δ3–9 hiPSC-CMs.[4] Binding to dystrophin might be necessary for desmin to be stably expressed in CMs, and desmin might reinforce the stability of cTnT and cTnI. Furthermore, decreased desmin might be involved in the pathogenesis of DMD-associated DCM, whereas increased desmin in Δ3–9 hiPSC-CMs might compensate for the impaired function of internally truncated dystrophin, which might partially explain the reduced severity in the phenotype of DCM in Δ3–9 patients. Further research is required to determine whether desmin modulates the DCM phenotype in patients with DMD. Although recovery of the expression of these proteins was expected through skipping therapy, no significant changes were observed in the evaluation during this short period. The extended culture or repeated administration of AOs might be required for the restoration of these proteins, despite the possibility that the decrease in these proteins might be an irreversible change caused by developmental defects caused by the absence of Dp427m.

Δ3–7 hiPSC-CMs specifically represented impaired adhesion, which may be explained by the decreased glycosylated α-DG, integrin, and ILK gene expression. These are major linkers between the ECM and cytoskeleton and are important in cell adhesion and mechano-transduction-related signaling pathways.[73]

Intracellular calcium overload caused by disruption of the sarcolemma is a promising hypothesis for the pathogenesis of DCM in DMD.[74] This can lead to mitochondrial dysfunction, which causes cell death and the acceleration of Ca^{2+}-dependent proteases, including CaMKII and protein kinase A, which could contribute to the development of HF and arrhythmia in patients with DMD and *mdx* mice.[75–77] This study recapitulated the increase in the activated form of CaMKII by auto-phosphorylation at T287 and oxidation at M281/M282, accompanied by increased phosphorylation of PLB at T17 downstream of activated CaMKII[78] in Δ3–7 hiPSC-CMs, indicating an intracellular calcium overload. The targets for activated CaMKII, including ion channels such as RyR2, SERCA2a, and Na^{+}/Ca^{2+} exchanger, and type II histone deacetylases, which regulate MEF2 signaling involved in myocardial hypertrophy, might also be enhanced, leading to aberrant excitation-contraction coupling and hypertrophy signaling in Δ3–7 hiPSC-CMs. AO-mediated exon skipping successfully decreased CaMKII auto-phosphorylation, which is

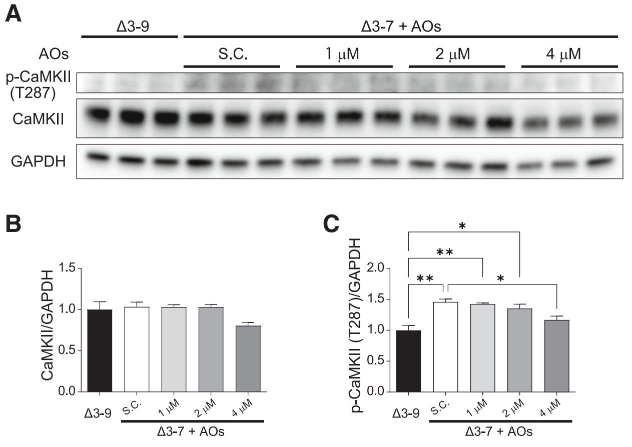

Figure 8. Exon skipping attenuates the phosphorylation of CaMKII

(A) Western blot analysis showing protein levels of CaMKIIδ and phosphorylated CaMKII (T287) in Δ3–9 and Δ3–7 hiPSC-CMs 14 d after treatment with vivo-Morpholinos at 0–4 μM. GAPDH was used as a loading control. (B and C) Quantification of protein expression of CaMKIIδ (B) and phosphorylated CaMKII (T287) (C) in A. Data are presented as mean ± SEM. *p < 0.05, **p < 0.01, ***p < 0.005.

speculated to be caused by the amelioration of calcium over-influx by the restoration of dystrophin.

Transcriptomic analysis of the three groups revealed the greatest similarity in gene expression profiles between Δ3–9 hiPSC-CMs and WT hiPSC-CMs by PCA, which might corroborate the mild cardiac phenotype in patients with Δ3–9 BMD.[23,31] Enrichment analysis indicated that defective signaling pathways associated with mechano-transduction, in which multiple proteins and protein complexes from inside the sarcomere to outside the sarcolemma and ECM, including DAPC, might have a strong impact on the pathology of DCM in Δ3–7 hiPSC-CMs.[79,80] The expression of the genes involved in these pathways was suggested to have the potential to approach the expression patterns in WT hiPSC-CMs and Δ3-9 hiPSC-CMs through exon skipping therapy. Among the genes involved in these pathways, however, *NPPA*, *NPPB*, *CASQ2*, *ANKRD1*, *ACTA1*, and *FSTL3* were prominently downregulated not only in Δ3–7 hiPSC-CMs, but also in Δ3–9 hiPSC-CMs. These genes are associated with cardiac hypertrophy and remodeling and are transcriptionally regulated by sarcomere-mediated mechano-transduction.[57,58,79] *NPPA* and *NPPB*, encoding atrial natriuretic peptide (ANP) and brain natriuretic peptide (BNP), respectively, synthesized mostly in CMs, are clinically used as markers for the progression of HF, of which the biological cardioprotective property has been well studied, leading to drug development.[81–83] In patients with HF caused by dystrophin-associated DCM, plasma BNP levels are reported to be lower relative to the severity of HF than in those with HF caused by other etiologies.[84–86] DCM modeling of hiPSC-CMs with mutations in *TNNT2* and frameshifting deletions (Δ44) in *DMD* have shown decreased *NPPA* and *NPPB* expression.[43,44] The precise mechanism of impaired ANP/BNP production and its biological effect in patients with DMD/BMD remain to be elucidated. However, the impaired production of natriuretic peptides in the heart tissue might contribute to HF progression in patients with DMD/BMD.

The isoform switching of sarcomeric genes, including *TNNI*, *MYH*, and *MYL*, was delayed in Δ3–7 hiPSC-CMs, consistent with previous studies, indicating maturation defects in DMD-hiPSC-CMs.[19] Among these, the decrease in *TNNI3/TNNI1*, a later maturation marker,[87] was significantly improved via exon skipping in Δ3–7 hiPSC-CMs, indicating that dystrophin may play an important role in regulating the transcriptomic switch of *TNNI*. Remarkably, numerous genes involved in ribosome biogenesis were downregulated in both Δ3–7 hiPSC-CMs and Δ3–9 hiPSC-CMs compared with those in WT hiPSC-CMs. Alteration of YAP signaling downstream of Hippo signaling has been demonstrated to be associated with the impairment of actin dynamics, following the decreased proliferation ability in DMD (Δ48–54) hiPSC-CMs.[21] Proteosome analysis of the myocardium from porcine DMD models showed a marked decrease in ribosome proteins with reduced heart weight and cell atrophy, suggesting deficient muscle protein synthesis caused by reduced translation activity due to impaired ribosome protein production.[88] The function of Δ3–9 dystrophin might be insufficient in terms of Hippo/YAP signal transduction and ribosome biogenesis.

Regarding the electrophysiological aspects, the spontaneous beating rate was increased in Δ3–7 hiPSC-CMs, and variability in the interbeat interval was increased both in Δ3–7 hiPSC-CMs and Δ3–9 hiPSC-CMs, which might correspond with the susceptibility to ventricular and sinus tachycardia and accelerated beat rate variability in patients with DMD.[74] Apparent arrhythmic transient was not detected in all groups in this study, which is inconsistent with previous studies on DMD modeling hiPSC-CMs by us and the other groups.[15,17,18] This discrepancy might be due to the differences in cell lines, mutations, cardiac differentiation protocols, and calcium indicators used. The analysis of monolayered hiPSC-CMs with field pacing revealed a significantly prolonged duration of both calcium and potential signals, as well as a prolonged rising phase of calcium transient in Δ3–7 hiPSC-CMs. These findings suggest the presence of ion channel malfunction and abnormalities in calcium handling in DMD hiPSC-CMs, which is consistent with previous studies,[15,18,19,43,89] and are considered phenomena that can explain electrocardiographic abnormalities in patients with DMD.[90] AO treatment improved the electrophysiological properties following partial restoration of the internally truncated dystrophin in Δ3–7 hiPSC-CMs. As a potential mechanism, abnormalities in the expression of channel genes were initially suspected, and RNA-seq data revealed changes in the gene expression of multiple ion channels that contribute to the action potential and calcium transient. However, significant fluctuations in that gene expression did not accompany the rapid improvement in electrophysiology achieved through skipping therapy. Therefore, it was considered highly likely that changes in the expression of channel genes do not directly contribute to therapeutic effects. Previous studies on DMD hiPSC-CMs have failed to come to a consensus on channel gene expression.[89,91] DAPC represented by α1-syntrophin plays an important role in the regulation

of ion channels on the sarcolemma of CMs, including $Na_V1.5$, Kir2.1, and Kir2.2, by interacting with them.[91,92] The dysfunction of DAPC is associated with cardiac conduction defects in *mdx* mice.[91,93] Jimenez-Vaquez et al. (2022) demonstrated that electrophysiological alterations were ameliorated by the overexpression of *SNTA1* encoding α1-syntrophin in hiPSC-CMs derived from patients with DMD.[4,89] Another dystrophin-associated protein, AHNAK1, modulates β-adrenergic regulation of L-type calcium channel (LTCC) activity in CMs.[48,50] The Δ3–9 dystrophin retains the binding domains for α1-syntrophin and AHNAK1 in its structure, and, for AHNAK1, dystrophin IP experiments have demonstrated that its binding ability in hiPSC-CMs is comparable to that in WT Dp427m. In addition, activated CaMKII accelerates the phosphorylation of the targets, including SERCA2a, RyR2, LTCCs, NCX, and PLN.[75] Consequently, it is hypothesized that the aberrant post-translational modification of channels on the sarcolemma and endoplasmic reticulum membrane by DAPC and CaMKII may account for the electrophysiological abnormalities observed in Δ3–7 hiPSC-CMs. Another possible explanation is that increased Dp71 may adversely affect channel function or activity in Δ3–7 hiPSC-CMs. These hypotheses might explain the rapid electrophysiological amelioration by the partial restoration of dystrophin and the decrease in Dp71 following treatment with AOs.

This study has several limitations. First, we used hiPSC-CMs, but these cannot fully represent patients' heart tissue because they are immature, resembling neonatal ventricular cells in terms of gene expression and function. Second, since the analysis relied on isogenic disease model cells derived from a single cell line, evidence for the correlation between the mutation and pathophysiology is not sufficiently strong. Third, this study's exon skipping experiment focused solely on the short-term effects of a single administration. Although we observed rapid electrophysiological improvements alongside the restoration of dystrophin expression, the expression patterns of proteins and genes following exon skipping therapy did not tend to resemble the pattern of Δ3–9. This might be attributed to the time required for the stabilization and functional recovery of DAPC, as well as potential changes in the expression of other genes and proteins after the restoration of dystrophin expression. Determining the optimal skipping level for functional recovery was not possible in this study. Last, the study was carried out *in vitro*, meaning that the delivery of AOs to the heart and the skipping efficiency in the tissue could not be investigated. Further studies using an ABD1-deficient animal model exhibiting a severe DCM phenotype would be required to fully assess the clinical application of this system.

In summary, our findings revealed that internally truncated dystrophin lacking ABS2 and ABS3 in ABD1 was biologically stable with substantial actin-binding function in Δ3–9 hiPSC-CMs, even in comparison with WT Dp427m. Furthermore, Δ3–9 hiPSC-CMs showed little electrophysiological impairment without acceleration of CaMKII auto-phosphorylation and oxidation, contrary to Δ3–7 hiPSC-CMs, although some molecular pathologies, including alterations in ribosomal and mitochondrial biogenesis and impaired mechano-transduction-related hypertrophy and remodeling, were shared with Δ3–7 hiPSC-CMs. We demonstrated that exon skipping by AOs targeting exon 8 efficiently induced exons 8–9 skipping to produce functional dystrophin and ameliorated electrophysiological abnormalities in Δ3–7 hiPSC-CMs. In conclusion, exon skipping therapy targeting ABD1 to convert the reading frame to Δ3–9 may be promising for treating DMD cardiomyopathy.

MATERIALS AND METHODS

Generation of a WT hiPSC line with a genetically encoded dual fluorescent indicator of voltage and intracellular calcium ions

All hiPSC experiments were performed using a genetically modified cell derivative from the 610B1 (RIKEN BRC, Tsukuba, Japan) hiPSC line, a commercially available WT control line derived from a healthy male. First, we developed a plasmid containing ASAP2s, a fluorescent green voltage indicator, and R-CaMP1.07, a fluorescent red calcium indicator. Briefly, a donor plasmid using the pSF-CAG-Ub-Puro (OGS600, Sigma-Aldrich, St. Louis, MO) cloning vector was generated, which consisted of approximately 800-bp-long homology arms flanking the *AAVS1* gRNA (CCAATCCTGTCCCTAGTG GCCCC) cut site surrounded by an 8.8-kb insert with two elements: a cassette bearing the CAG promoter that drives both ASAP2s and subsequent R-CaMP1.07 expression using self-cleaved 2A peptide and a second cassette encoding for Ubc-driven expression of puromycin resistance gene surrounded by *LoxP* sites, as illustrated in Figure 1A. We obtained the ASAP2s sequence from Addgene (#101274, Addgene, Watertown, MA) and the R-CaMP1.07[94] plasmid as a courtesy of Dr. Nakai. Plasmids expressing sgRNA and Cas9 were constructed using the Guide-it CRISPR-Cas9 system (Takara Bio, Kusatsu, Japan) following the manufacturer's protocols. A day prior to electroporation, 610B1 hiPSCs were treated with 10 μM valproic acid and 10 μM Y27632 for 24 h. ASAP2s-RCaMP1.07 donor plasmid (6 μg), Cas9/sgRNA plasmid (3 μg), and RAD51-expressing plasmids[95] (1 μg) were co-electroporated using Nucleofector 2b (Lonza, Basel, Switzerland) with the B-16 program into 1×10^6 hiPSCs. Two days after electroporation, transfected cells were selected with 1 μg/mL puromycin for 4 d. After expansion, PCR genotyping was performed to examine whether the clones were correctly targeted. The pCAG-iCre plasmid (10 μg; #89573, Addgene) was electroporated into the correctly targeted clones to remove the selection cassette. Genomic DNA was isolated using the DNeasy Blood and Tissue Kit (Qiagen, Venlo, the Netherlands) following the standard protocol. The region around the cut site was amplified with PrimeSTAR GXL DNA polymerase (Takara Bio). The primers used are listed in Table S1.

Generation of isogenic DMD exonic deleted model hiPSC lines

To generate isogenic model lines of the DMD exon 3–7 deletion and exon 3–9 deletion of ASAP2s/R-CaMP1.07, transgenic 610B1 hiPSCs were electroporated with a specific pair of Cas9/sgRNA plasmids targeting DMD introns 2 and 7 (4 μg each) for deletion of exons 3–7 and targeting introns 2 and 9 for deletion of exons 3–9[15] (Table S2). Cas9/sgRNA plasmids were sub-cloned using the method described above. Potential off-target genomic sites for each sgRNA were computationally predicted using an online tool for genome editing with the

CRSPR-Cas9 system, CRISPOR.org (http://crispor.org)[96] (Stable S3). Sanger sequencing of the predicted potential off-targets on undifferentiated cell-extracted genomic DNA revealed no sgRNA-mediated nonspecific cleavage by Cas9 for each cell line.

Cardiac differentiation and maturation

Undifferentiated hiPSCs were induced to differentiate into CMs using a previously reported protocol.[97] Briefly, undifferentiated hiPSCs were plated on an MG (Corning, Corning, NY)-coated culture dish and cultured in Essential 8 medium (Thermo Fisher Scientific, Waltham, MA) supplemented with 1 μM CHIR99021 (Sigma-Aldrich). On day 0, the medium was replaced with RPMI 1640 medium (Thermo Fisher Scientific) supplemented with B27 (RPMI/B27) without insulin (RPMI/B27-ins) with 100 ng/mL activin A (R&D Systems, Minneapolis, MN) and MG. On day 1, the medium was replaced with 10 ng/mL BMP-4 (R&D Systems) and 1 μM CHIR99021. On days 3–4, the medium was replaced with 1 μM XAV939 (R&D systems), a Wnt inhibitor. On day 7, insulin was added to RPMI/B27 (RPMI/B27+ins); the medium was changed every 2 d. Spontaneous beating was observed on days 7–10. On day 9, the cells were exposed to a glucose- and glutamine-free medium supplemented with 4 mM lactate and StemFit medium AS501 (Ajinomoto, Tokyo, Japan) to enrich CMs.[36,39,98] To promote cell proliferation, cells were re-plated as dissociated single cells with 2 mM CHIR99021 in 10% knockout serum replacement (Thermo Fisher Scientific) in RPMI/B27+ins onto an MG coated 10 cm culture dish at 4–8 × 10^4/cm^2 density on day 13. On day 15, the medium was replaced with 2 μM CHIR99021, and on day 17 the medium was replaced with 10 μM Y-27632 and 2 μM Wnt-C59, a Wnt inhibitor in RPMI/B27+ins.[38] On day 18, the cells were replated in MG-coated dishes at a density of 3 × 10^5/cm^2. To enhance CM maturation on days 21–35, the culture medium was refreshed daily with RPMI/B27+ins supplemented with 100 nM T3 and 1 μM Dex.[37] Starting from day 35, hiPSC-CMs were maintained in RPMI/B27+ins, with the medium changed every 3–4 d until harvesting for characterization using immunofluorescence, mRNA expression, and protein analyses. Cardiac purity was determined by immunostaining for cTnT (clone CT3), followed by anti-mouse IgG_1 conjugated with phycoerythrin, using FACS Canto II (BD Biosciences, Franklin Lakes, NJ). Cell diameter was determined using the standard curve related to forward scatter and cell size generated using the SPHERO Particle Size Standard Kit (#PPS-6K, Spherotech, Lake Forest, IL).

Immunofluorescence staining of hiPSC-CMs in cell culture

Cultured cells were fixed with 4% paraformaldehyde (Sigma-Aldrich) in PBS for 10 min at (room temperature [RT]; 24 ± 2°C). Next, cells were washed with 0.05% Tween 20 (Thermo Fisher Scientific) in PBS (PBST) and then blocked with 5% normal goat serum in PBST for 1 h at RT. Subsequently, primary antibody incubation was performed overnight at 4°C in a blocking solution. The primary antibodies used in these studies were mouse anti-dystrophin [mandys8] (Sigma-Aldrich; D8168, 1:200), mouse anti-cTnT (Thermo Fisher Scientific; MA5-12960, 1:300), rabbit anti-cTnT [EPR3695] (Abcam, Cambridge, UK; ab91605 1:300), rabbit anti-cTnI (Abcam ab52862; 1:300), and mouse anti-α sarcomeric actinin [EA-53] (Abcam; ab9465, 1:300). The samples were washed three times with PBST and incubated with Alexa Fluor secondary antibodies (Thermo Fisher Scientific) 1:500 in PBST for 1 h at RT. Cells were washed again with PBST (3 × 5 min), followed by the 0.5 mg/mL DAPI (Sigma-Aldrich; D9542) nuclei counterstain in PBS for 1 min at RT. Images were acquired using a fluorescent microscope (BZ-X710; Keyence, Osaka, Japan). For measuring sarcomere length, we selected myofibrils that contained 11 continuous, well recognized alpha-actinin-positive bands and divided the length value by 10.

RNA extraction and real-time qPCR

RNA was isolated from hiPSC-CMs using the RNeasy Mini Kit (Qiagen). Human fetal heart total RNA (#636532, Takara Bio) and human adult heart RNA (# 636583, Takara Bio) were purchased. Reverse transcription of RNA to cDNA was performed using the Superscript IV Reverse Transcriptase Kit (Thermo Fisher Scientific) following the manufacturer's instructions, using random hexamers. qPCR was performed using Fast SYBR Green Master Mix (Thermo Fisher Scientific) and QuantStudio 3 (Thermo Fisher Scientific) with the primers listed in Table S1. Thermal cycling conditions were as follows: initial denaturation at 95°C for 20 s, 40 cycles at 95°C for 3 s, and 60°C for 30 s. We calculated mRNA fold expression using the ΔΔCT method with *GAPDH* as the housekeeping gene. All experiments were conducted in duplicate.

RNA-seq

RNA was isolated from day-48 hiPSC-CMs cultured in six-well plates using the RNeasy Mini Kit, following the manufacturer's instructions. All RNA was quantified using a Qubit 3.0 Fluorometer (Thermo Fisher Scientific) and analyzed using an Agilent 2100 Bioanalyzer (Agilent Technologies, Santa Clara, CA) to ensure RNA integrity (all samples had RIN scores ≥9.1). mRNA was enriched with poly(A) selection, and 50-bp paired-end RNA-seq was completed on the DNBSEQ platform at the Beijing Genomics Institute (BGI, Shenzhen, China). Raw reads were filtered using SOAP and SOAPnuke,[99] and clean reads were mapped to the transcriptome of the RefSeq database using Bowtie2.[100] Gene expression was counted by RSEM[101] and normalized as transcripts per kilobase of exon model per million mapped reads. We used DESeq2 to evaluate the differential expression. DEGs were identified by a false discovery rate-adjusted p value < 0.05.[102] All data were submitted to the online tool Dr. TOM software (BGI) for PCA and GO and pathway enrichment analyses.

Western blotting analysis and coIP

Proteins were extracted by directly lysing day 48 hiPSC-CMs in cell culture wells with M-PER mammalian protein extraction reagent (Thermo Fisher Scientific) supplemented with Protease/Phosphatase Inhibitor Cocktail (100×) (Cell Signaling Technology, Danvers, MA). Protein samples were mixed with 4× Laemmli sample buffer (Bio-Rad, Hercules, CA) and boiled for 5 min at 95°C. Samples containing 1.25–10 μg total protein were separated on an Any kD mini-Protean TGX gel (Bio-Rad) and transferred onto polyvinylidene

fluoride membranes. The membranes were blocked with Bullet Blocking One (Nacalai Tesque, Kyoto, Japan). The membranes were incubated with specific primary antibodies overnight at 4°C, followed by horseradish peroxidase (HRP)-conjugated secondary antibodies for 1 h at RT.

Primary antibodies were rabbit mouse anti-dystrophin (Abcam 15277: 1:3000), sheep anti-DG (R&D Systems AF6868; 1:3,000), rabbit anti-α-actin (HUABIO, Woburn, MA, 0407-3; 1:3,000), mouse anti-desmin [Y66] (Abcam ab32362; 1:3,000), rabbit anti-cTnT [EPR3695] (Abcam ab91605; 1:3,000), rabbit anti-cTnI (Abcam ab152862; 1:3,000), mouse anti-AHNAK (GeneTex, Irvine, CA, GTX80164; 1:3,000), mouse anti-LDB3 (Abcam ab110003; 1:3,000), mouse anti-CRYAB (Enzo Life Sciences, Farmingdale, NY, ADI-SPA-222-D; 1:1,000), rabbit anti-CaMKII delta (GeneTex GTX111401; 1:3,000), rabbit anti-CaMKII (Thr287) (Thermo Fisher Scientific, PA5-37833; 1:3,000), rabbit anti-CaMKII (oxidized) (GeneTex GTX 36254; 1:3,000), mouse anti-PLB [clone A1] (Badrilla, Leeds, UK, A010-14; 1:3,000), rabbit anti-PLB (pThr17) (Badrilla, A010-13AP; 1:3,000), and mouse anti-GAPDH (Proteintech, Rosemont, IL, 60004-1-Ig; 1:10,000). The secondary antibodies were goat anti-mouse IgG HRP-conjugate (Abcam, ab97023; 1:30,000), goat anti-rabbit IgG HRP-conjugate (Abcam, ab205718; 1:30,000), and donkey anti-sheep IgG HRP-conjugate (R&D Systems, HAF016; 1:30,000). Proteins were visualized using Western BLoT Hyper HRP Substrate (Takara Bio) and the ChemiDoc Touch Imaging System (Bio-Rad). CoIP was performed using the Dynabeads Co-Immunoprecipitation Kit (Thermo Fisher Scientific, Novex 14321D), following the manufacturer's instructions. Next, 35 mg mouse anti-human dystrophin antibody (Santa Cruz Biotechnology, Dallas, TX; sc-73592) was coupled with 5 mg Dynabeads M-270 epoxy overnight at 37°C. Antibody-coupled beads were washed and extracted using a magnetic field. Lysate (50 mg) derived from day 48 hiPSC-CMs in extraction buffer (25 mM NaCl) were added to 1.5 mg antibody-coupled beads and incubated overnight at 4°C. The protein-antibody-coupled bead complex was washed, and the purified protein samples were separated using a magnetic field. A fraction was mixed with 4 × Laemmli sample buffer (Bio-Rad), boiled for 5 min at 95°C, and used for SDS-PAGE and western blotting to probe for dystrophin, α-actin, desmin, cTnT, AHNAK1, Cypher, and CRYAB. To analyze dystrophin isoforms, we performed western blotting in the following manner. After mixing with NuPAGE LDS Sample Buffer (Thermo Fisher Scientific), lysates were denatured at 70°C for 10 min, electrophoresed on a 3%–8% NuPAGE Novex Tris-acetate gel (Thermo Fisher Scientific) at 150 V for 75 min, and transferred onto polyvinylidene fluoride membranes (Bio-Rad). The membranes were sequentially incubated with primary and secondary antibodies using an iBind Flex Western Device (Thermo Fisher Scientific). Rabbit anti-dystrophin (Abcam, ab15277; 1:400), mouse anti-glyceraldehyde-3-phosphate dehydrogenase (GAPDH; EMD Millipore, MAB374; 1:2,000), and anti-α tubulin (Millipore, 05–829; 1:1,000) were used as the primary antibodies. Goat anti-rabbit IgG (H+L) HRP conjugate (Bio-Rad, 1706515; 1:2,000) was used as the secondary antibody. Protein expression was detected using the ECL Prime Western Blotting Detection Reagent (Cytiva, Parramatta, Australia).

Voltage and calcium imaging

Monolayered beating hiPSC-CMs were dissociated with 0.05% trypsin EDTA for 10 min, and pellets were resuspended in RPMI/B27+ins with 10 μM Y-27632. Cells were reseeded on the MG-coated 26-mm glass bottom of a 35-mm dish (IWAKI, Tokyo, Japan) at a density of 6,000 cells/cm^2 for single-cell spontaneous beating analysis and at a density of 75,000 cells/cm^2 onto an MG-coated 12-mm glass bottom for monolayered sheet analysis. Next, 5–7 d after plating, hiPSC-CMs were loaded with Tyrode's solution (140 mM NaCl, 0.5 mM $MgCl_2$, 1.8 mM $CaCl_2$, 5.4 mM KCl, 5.5 mM glucose, and 5.5 mM HEPES; pH 7.4), and voltage and calcium imaging was performed using a microscope (BZ-X710, Keyence) at 37°C. For single-cell analysis, spontaneous intracellular calcium transient was captured in a red fluorescent signal as R-CaMP1.07 for 1 min per region at 25 Hz. Three different fields of view, including two to eight spontaneously beating, isolated single cells selected as regions of interest, were acquired per well. For sheet analysis, hiPSC-CMs were paced at 1/3 Hz (50V, 1 ms duration) using an electric stimulator (SEN-3401, Nihon Kohden, Tokyo, Japan) with platinum electrodes (RC-37FS, Warner Instruments, Hamden, CT), which captured two fluorescent signals as ASAP2s (ET470/40) and RCaMP1.07 (ET545/25) at 60 Hz for 12 s per region. One field of view was acquired per well. Data were analyzed semi-manually using Excel (Microsoft, Redmond, WA) and HRV Tool and Peak analysis Tool in LabChart Pro 8 (ADInstruments, Dunedin, New Zealand) with the following settings: automatic recognition of resting membrane potential, TStart 10% of height away from resting membrane potential, and TRise and TFall defined between 10% and 90% of the peak height. APD30/60/90 was defined as the time from the maximum of the potential until 30%/60%/90% signal decay. For calcium transient analysis, the time to peak was defined as the time from the start to the maximum of the transient, and FWHM was defined as the distance between the two half-maximum points. For analysis of hiPSC-CMs without transfected ASAP2s/RCaMP, Fluo-4AM (AAT Bioquest, Inc., Sunnyvale, CA) and FluoVolt (Thermo Fisher Scientific) were used following the manufacturer's instructions.

Exon 8 (+9) skipping for DMD Δ3–7 hiPSC-CMs

For exon 8(+9) skipping, we applied previously reported sequences (H8A(-6 + 18) 5′-ATAGGTGGTATCAACATCTGTAA-3′)[32] targeting exon 8 of DMD mRNA for Vivo-Morpholinos. Vivo-Morpholinos and Vivo-standard control (S.C.) were obtained from Gene Tools, LLC (Philomath, OR). On day 36, hiPSC-CMs were treated with Vivo-Morpholinos (0.5, 1.0, 2.0, and 4 μM in RPMI/B27+ins) and S.C. (4 μM in RPMI/B27+ins) for 12 h. The cells were maintained in RPMI/B27+ medium and changed every 3 d for 14 d. On day 60, hiPSC-CMs were reseeded on glass-bottom dishes for imaging analysis, fixed with 4% paraformaldehyde for immunofluorescence staining, lysed with RNeasy Mini Kit for mRNA analysis, and lysed with

the M-PER Mammalian Protein Extraction Reagent for western blotting analysis.

LDH assay

On day 36, WT hiPSC-CMs were reseeded at a density of 4 × 10^4 cells/cm^2 in an MG-coated 96-well plate and cultured for 2 d before being replaced with RPMI B27+ media containing Vivo-Morpholinos (0.25–8 μM). After 10 h of incubation, a cytotoxicity LDH assay was performed using the Cytotoxicity LDH Assay WST (Dojindo Laboratories, Kumamoto, Japan), following the manufacturer's instructions. A microplate reader (SpectraMax iD5; Molecular Devices, San Jose, CA) was used to measure the absorbance at 490 nm.

Statistical analysis

Results are displayed as the mean ± SEM. Statistical analyses were performed using unpaired t-tests for two-group comparisons and a one-way ANOVA with Tukey's post hoc test for multiple comparisons. Values of $p < 0.05$ were considered significant. Statistical analyses were performed using GraphPad Prism version 9.0 (GraphPad Software, Inc., La Jolla, CA).

DATA AND CODE AVAILABILITY

Raw data necessary for confirming the results reported in the paper are presented herein or are available from the authors upon request.

SUPPLEMENTAL INFORMATION

Supplemental information can be found online at https://doi.org/10.1016/j.omtn.2023.102060.

ACKNOWLEDGMENTS

This research was supported by the JSPS KAKENHI grant number 18H02577 and 20K08253, Miyata Foundation Board for Pediatric Cardiovascular Research to N.S/, and Grants-in-Aid for Research on Nervous and Mental Disorders [grant number 2–6]. The authors thank Y. Karatsu (Shinshu University, Japan) for their technical assistance and Dr. Hiroyuki Mizuguchi (Osaka University, Japan) for providing the RAD51-expressing plasmid.

AUTHOR CONTRIBUTIONS

N.S. performed experiments and wrote the manuscript; Y.X., M.S., S.K., Y.K., M.A., K.N., M.I., Y.H., and T.K. performed experiments; H.K., H.I., S.C., J.N., S.T., K.F., D.M., A.N., and Y.S. analyzed the results and supervised the design and execution of the study and manuscript preparation. All authors have reviewed the manuscript.

DECLARATION OF INTERESTS

K.F. is a co-founder and CEO of Heartseed Inc. S.T. is an advisor for Heartseed, Inc. S.T. and K.F. have owned equity in Heartseed, Inc.

REFERENCES

1. Emery, A.E.H. (2002). The muscular dystrophies. Lancet *359*, 687–695.
2. Bushby, K., Finkel, R., Birnkrant, D.J., Case, L.E., Clemens, P.R., Cripe, L., Kaul, A., Kinnett, K., McDonald, C., Pandya, S., et al. (2010). Diagnosis and management of Duchenne muscular dystrophy, part 1: diagnosis, and pharmacological and psychosocial management. Lancet Neurol. *9*, 77–93.
3. Mias-Lucquin, D., Dos Santos Morais, R., Chéron, A., Lagarrigue, M., Winder, S.J., Chenuel, T., Pérez, J., Appavou, M.S., Martel, A., Alviset, G., et al. (2020). How the central domain of dystrophin acts to bridge F-actin to sarcolemmal lipids. J. Struct. Biol. *209*, 107411.
4. Duan, D., Goemans, N., Takeda, S., Mercuri, E., and Aartsma-Rus, A. (2021). Duchenne muscular dystrophy. Nat. Rev. Dis. Prim. *7*, 13.
5. Hoffman, E.P., Knudson, C.M., Campbell, K.P., and Kunkel, L.M. (1987). Subcellular fractionation of dystrophin to the triads of skeletal muscle. Nature *330*, 754–758.
6. Campbell, K.P., and Kahl, S.D. (1989). Association of dystrophin and an integral membrane glycoprotein. Nature *338*, 259–262.
7. Dumont, N.A., Wang, Y.X., von Maltzahn, J., Pasut, A., Bentzinger, C.F., Brun, C.E., and Rudnicki, M.A. (2015). Dystrophin expression in muscle stem cells regulates their polarity and asymmetric division. Nat. Med. *21*, 1455–1463.
8. Ryder, S., Leadley, R.M., Armstrong, N., Westwood, M., de Kock, S., Butt, T., Jain, M., and Kleijnen, J. (2017). The burden, epidemiology, costs and treatment for Duchenne muscular dystrophy: an evidence review. Orphanet J. Rare Dis. *12*, 79.
9. Hor, K.N., Mah, M.L., Johnston, P., Cripe, T.P., and Cripe, L.H. (2018). Advances in the diagnosis and management of cardiomyopathy in Duchenne muscular dystrophy. Neuromuscul. Disord. *28*, 711–716.
10. Yazaki, M., Yoshida, K., Nakamura, A., Koyama, J., Nanba, T., Ohori, N., and Ikeda, S. (1999). Clinical characteristics of aged Becker muscular dystrophy patients with onset after 30 years. Eur. Neurol. *42*, 145–149.
11. Ferreiro, V., Giliberto, F., Muñiz, G.M.N., Francipane, L., Marzese, D.M., Mampel, A., Roqué, M., Frechtel, G.D., and Szijan, I. (2009). Asymptomatic Becker muscular dystrophy in a family with a multiexon deletion. Muscle Nerve *39*, 239–243.
12. Nakamura, A., Shiba, N., Miyazaki, D., Nishizawa, H., Inaba, Y., Fueki, N., Maruyama, R., Echigoya, Y., and Yokota, T. (2017). Comparison of the phenotypes of patients harboring in-frame deletions starting at exon 45 in the Duchenne muscular dystrophy gene indicates potential for the development of exon skipping therapy. J. Hum. Genet. *62*, 459–463.
13. Bladen, C.L., Salgado, D., Monges, S., Foncuberta, M.E., Kekou, K., Kosma, K., Dawkins, H., Lamont, L., Roy, A.J., Chamova, T., et al. (2015). The TREAT-NMD DMD Global Database: analysis of more than 7,000 Duchenne muscular dystrophy mutations. Hum. Mutat. *36*, 395–402.
14. Guan, X., Mack, D.L., Moreno, C.M., Strande, J.L., Mathieu, J., Shi, Y., Markert, C.D., Wang, Z., Liu, G., Lawlor, M.W., et al. (2014). Dystrophin-deficient cardiomyocytes derived from human urine: new biologic reagents for drug discovery. Stem Cell Res. *12*, 467–480.
15. Kyrychenko, V., Kyrychenko, S., Tiburcy, M., Shelton, J.M., Long, C., Schneider, J.W., Zimmermann, W.H., Bassel-Duby, R., and Olson, E.N. (2017). Functional correction of dystrophin actin binding domain mutations by genome editing. JCI Insight *2*, e95918.
16. Tsurumi, F., Baba, S., Yoshinaga, D., Umeda, K., Hirata, T., Takita, J., and Heike, T. (2019). The intracellular Ca2+ concentration is elevated in cardiomyocytes differentiated from hiPSCs derived from a Duchenne muscular dystrophy patient. PLoS One *14*, e0213768.
17. Sato, M., Shiba, N., Miyazaki, D., Shiba, Y., Echigoya, Y., Yokota, T., Takizawa, H., Aoki, Y., Takeda, S., and Nakamura, A. (2019). Amelioration of intracellular Ca(2+) regulation by exon-45 skipping in Duchenne muscular dystrophy-induced pluripotent stem cell-derived cardiomyocytes. Biochem. Biophys. Res. Commun. *520*, 179–185.
18. Eisen, B., Ben Jehuda, R., Cuttitta, A.J., Mekies, L.N., Shemer, Y., Baskin, P., Reiter, I., Willi, L., Freimark, D., Gherghiceanu, M., et al. (2019). Electrophysiological abnormalities in induced pluripotent stem cell-derived cardiomyocytes generated from Duchenne muscular dystrophy patients. J. Cell Mol. Med. *23*, 2125–2135.
19. Pioner, J.M., Guan, X., Klaiman, J.M., Racca, A.W., Pabon, L., Muskheli, V., Macadangdang, J., Ferrantini, C., Hoopmann, M.R., Moritz, R.L., et al. (2020). Absence of full-length dystrophin impairs normal maturation and contraction of cardiomyocytes derived from human-induced pluripotent stem cells. Cardiovasc. Res. *116*, 368–382.

20. Kamdar, F., Das, S., Gong, W., Klaassen Kamdar, A., Meyers, T.A., Shah, P., Ervasti, J.M., Townsend, D., Kamp, T.J., Wu, J.C., et al. (2020). Stem Cell-Derived Cardiomyocytes and Beta-Adrenergic Receptor Blockade in Duchenne Muscular Dystrophy Cardiomyopathy. J. Am. Coll. Cardiol. *75*, 1159–1174.

21. Yasutake, H., Lee, J.K., Hashimoto, A., Masuyama, K., Li, J., Kuramoto, Y., Higo, S., Hikoso, S., Hidaka, K., Naito, A.T., et al. (2021). Decreased YAP activity reduces proliferative ability in human induced pluripotent stem cell of duchenne muscular dystrophy derived cardiomyocytes. Sci. Rep. *11*, 10351.

22. Tuffery-Giraud, S., Béroud, C., Leturcq, F., Yaou, R.B., Hamroun, D., Michel-Calemard, L., Moizard, M.P., Bernard, R., Cossée, M., Boisseau, P., et al. (2009). Genotype-phenotype analysis in 2,405 patients with a dystrophinopathy using the UMD-DMD database: a model of nationwide knowledgebase. Hum. Mutat. *30*, 934–945.

23. Nakamura, A., Fueki, N., Shiba, N., Motoki, H., Miyazaki, D., Nishizawa, H., Echigoya, Y., Yokota, T., Aoki, Y., and Takeda, S. (2016). Deletion of exons 3-9 encompassing a mutational hot spot in the DMD gene presents an asymptomatic phenotype, indicating a target region for multiexon skipping therapy. J. Hum. Genet. *61*, 663–667.

24. Corrado, K., Mills, P.L., and Chamberlain, J.S. (1994). Deletion analysis of the dystrophin-actin binding domain. FEBS Lett. *344*, 255–260.

25. Novaković, I., Bojić, D., Todorović, S., Apostolski, S., Luković, L., Stefanović, D., and Milasin, J. (2005). Proximal dystrophin gene deletions and protein alterations in becker muscular dystrophy. Ann. N. Y. Acad. Sci. *1048*, 406–410.

26. Banks, G.B., Gregorevic, P., Allen, J.M., Finn, E.E., and Chamberlain, J.S. (2007). Functional capacity of dystrophins carrying deletions in the N-terminal actin-binding domain. Hum. Mol. Genet. *16*, 2105–2113.

27. Henderson, D.M., Lee, A., and Ervasti, J.M. (2010). Disease-causing missense mutations in actin binding domain 1 of dystrophin induce thermodynamic instability and protein aggregation. Proc. Natl. Acad. Sci. USA *107*, 9632–9637.

28. Toh, Z.Y.C., Thandar Aung-Htut, M., Pinniger, G., Adams, A.M., Krishnaswarmy, S., Wong, B.L., Fletcher, S., and Wilton, S.D. (2016). Deletion of Dystrophin In-Frame Exon 5 Leads to a Severe Phenotype: Guidance for Exon Skipping Strategies. PLoS One *11*, e0145620.

29. Gibbs, E.M., Barthélémy, F., Douine, E.D., Hardiman, N.C., Shieh, P.B., Khanlou, N., Crosbie, R.H., Nelson, S.F., and Miceli, M.C. (2019). Large in-frame 5' deletions in DMD associated with mild Duchenne muscular dystrophy: Two case reports and a review of the literature. Neuromuscul. Disord. *29*, 863–873.

30. Muntoni, F., Gobbi, P., Sewry, C., Sherratt, T., Taylor, J., Sandhu, S.K., Abbs, S., Roberts, R., Hodgson, S.V., Bobrow, M., et al. (1994). Deletions in the 5' region of dystrophin and resulting phenotypes. J. Med. Genet. *31*, 843–847.

31. Heald, A., Anderson, L.V., Bushby, K.M., and Shaw, P.J. (1994). Becker muscular dystrophy with onset after 60 years. Neurology *44*, 2388–2390.

32. Fletcher, S., Adkin, C.F., Meloni, P., Wong, B., Muntoni, F., Kole, R., Fragall, C., Greer, K., Johnsen, R., and Wilton, S.D. (2012). Targeted exon skipping to address "leaky" mutations in the dystrophin gene. Mol. Ther. Nucleic Acids *1*, e48.

33. Chamberland, S., Yang, H.H., Pan, M.M., Evans, S.W., Guan, S., Chavarha, M., Yang, Y., Salesse, C., Wu, H., Wu, J.C., et al. (2017). Fast two-photon imaging of subcellular voltage dynamics in neuronal tissue with genetically encoded indicators. Elife *6*, e25690.

34. Bethge, P., Carta, S., Lorenzo, D.A., Egolf, L., Goniotaki, D., Madisen, L., Voigt, F.F., Chen, J.L., Schneider, B., Ohkura, M., et al. (2017). An R-CaMP1.07 reporter mouse for cell-type-specific expression of a sensitive red fluorescent calcium indicator. PLoS One *12*, e0179460.

35. Ichimura, H., Kadota, S., Kashihara, T., Yamada, M., Ito, K., Kobayashi, H., Tanaka, Y., Shiba, N., Chuma, S., Tohyama, S., et al. (2020). Increased predominance of the matured ventricular subtype in embryonic stem cell-derived cardiomyocytes in vivo. Sci. Rep. *10*, 11883.

36. Tohyama, S., Hattori, F., Sano, M., Hishiki, T., Nagahata, Y., Matsuura, T., Hashimoto, H., Suzuki, T., Yamashita, H., Satoh, Y., et al. (2013). Distinct metabolic flow enables large-scale purification of mouse and human pluripotent stem cell-derived cardiomyocytes. Cell Stem Cell *12*, 127–137.

37. Parikh, S.S., Blackwell, D.J., Gomez-Hurtado, N., Frisk, M., Wang, L., Kim, K., Dahl, C.P., Fiane, A., Tønnessen, T., Kryshtal, D.O., et al. (2017). Thyroid and Glucocorticoid Hormones Promote Functional T-Tubule Development in Human-Induced Pluripotent Stem Cell-Derived Cardiomyocytes. Circ. Res. *121*, 1323–1330.

38. Maas, R.G.C., Lee, S., Harakalova, M., Snijders Blok, C.J.B., Goodyer, W.R., Hjortnaes, J., Doevendans, P.A.F.M., Van Laake, L.W., van der Velden, J., Asselbergs, F.W., et al. (2021). Massive expansion and cryopreservation of functional human induced pluripotent stem cell-derived cardiomyocytes. STAR Protoc. *2*, 100334.

39. Tohyama, S., Fujita, J., Hishiki, T., Matsuura, T., Hattori, F., Ohno, R., Kanazawa, H., Seki, T., Nakajima, K., Kishino, Y., et al. (2016). Glutamine oxidation is indispensable for survival of human pluripotent stem cells. Cell Metabol. *23*, 663–674.

40. Gilbert, G., Kadur Nagaraju, C., Duelen, R., Amoni, M., Bobin, P., Eschenhagen, T., Roderick, H.L., Sampaolesi, M., and Sipido, K.R. (2021). Incomplete Assembly of the Dystrophin-Associated Protein Complex in 2D and 3D-Cultured Human Induced Pluripotent Stem Cell-Derived Cardiomyocytes. Front. Cell Dev. Biol. *9*, 737840.

41. Bedada, F.B., Chan, S.S.K., Metzger, S.K., Zhang, L., Zhang, J., Garry, D.J., Kamp, T.J., Kyba, M., and Metzger, J.M. (2014). Acquisition of a quantitative, stoichiometrically conserved ratiometric marker of maturation status in stem cell-derived cardiac myocytes. Stem Cell Rep. *3*, 594–605.

42. de Feraudy, Y., Ben Yaou, R., Wahbi, K., Stalens, C., Stantzou, A., Laugel, V., Desguerre, I., FILNEMUS Network, Servais, L., Leturcq, F., and Amthor, H. (2021). Very Low Residual Dystrophin Quantity Is Associated with Milder Dystrophinopathy. Ann. Neurol. *89*, 280–292.

43. Atmanli, A., Chai, A.C., Cui, M., Wang, Z., Nishiyama, T., Bassel-Duby, R., and Olson, E.N. (2021). Cardiac Myoediting Attenuates Cardiac Abnormalities in Human and Mouse Models of Duchenne Muscular Dystrophy. Circ. Res. *129*, 602–616.

44. Pettinato, A.M., Ladha, F.A., Mellert, D.J., Legere, N., Cohn, R., Romano, R., Thakar, K., Chen, Y.S., and Hinson, J.T. (2020). Development of a Cardiac Sarcomere Functional Genomics Platform to Enable Scalable Interrogation of Human TNNT2 Variants. Circulation *142*, 2262–2275.

45. Matsumura, K., Ervasti, J.M., Ohlendieck, K., Kahl, S.D., and Campbell, K.P. (1992). Association of dystrophin-related protein with dystrophin-associated proteins in mdx mouse muscle. Nature *360*, 588–591.

46. Parvatiyar, M.S., Brownstein, A.J., Kanashiro-Takeuchi, R.M., Collado, J.R., Dieseldorff Jones, K.M., Gopal, J., Hammond, K.G., Marshall, J.L., Ferrel, A., Beedle, A.M., et al. (2019). Stabilization of the cardiac sarcolemma by sarcospan rescues DMD-associated cardiomyopathy. JCI Insight *5*, e123855.

47. Vatta, M., Mohapatra, B., Jimenez, S., Sanchez, X., Faulkner, G., Perles, Z., Sinagra, G., Lin, J.H., Vu, T.M., Zhou, Q., et al. (2003). Mutations in Cypher/ZASP in patients with dilated cardiomyopathy and left ventricular non-compaction. J. Am. Coll. Cardiol. *42*, 2014–2027.

48. Haase, H., Alvarez, J., Petzhold, D., Doller, A., Behlke, J., Erdmann, J., Hetzer, R., Regitz-Zagrosek, V., Vassort, G., and Morano, I. (2005). Ahnak is critical for cardiac Ca(V)1.2 calcium channel function and its beta-adrenergic regulation. Faseb. J. *19*, 1969–1977.

49. Inagaki, N., Hayashi, T., Arimura, T., Koga, Y., Takahashi, M., Shibata, H., Teraoka, K., Chikamori, T., Yamashina, A., and Kimura, A. (2006). Alpha B-crystallin mutation in dilated cardiomyopathy. Biochem. Biophys. Res. Commun. *342*, 379–386.

50. Johnson, E.K., Zhang, L., Adams, M.E., Phillips, A., Freitas, M.A., Froehner, S.C., Green-Church, K.B., and Montanaro, F. (2012). Proteomic analysis reveals new cardiac-specific dystrophin-associated proteins. PLoS One *7*, e43515.

51. Leyton-Mange, J.S., Mills, R.W., Macri, V.S., Jang, M.Y., Butte, F.N., Ellinor, P.T., and Milan, D.J. (2014). Rapid cellular phenotyping of human pluripotent stem cell-derived cardiomyocytes using a genetically encoded fluorescent voltage sensor. Stem Cell Rep. *2*, 163–170.

52. Shinnawi, R., Huber, I., Maizels, L., Shaheen, N., Gepstein, A., Arbel, G., Tijsen, A.J., and Gepstein, L. (2015). Monitoring Human-Induced Pluripotent Stem Cell-Derived Cardiomyocytes with Genetically Encoded Calcium and Voltage Fluorescent Reporters. Stem Cell Rep. *5*, 582–596.

53. Robinson, P., Sparrow, A.J., Psaras, Y., Steeples, V., Simon, J.N., Broyles, C.N., Chang, Y.F., Brook, F.A., Wang, Y.J., Blease, A., et al. (2023). Comparing the effects

of chemical Ca(2+) dyes and R-GECO on contractility and Ca(2+) transients in adult and human iPSC cardiomyocytes. J. Mol. Cell. Cardiol. *180*, 44–57.

54. Thomas, T.O., Morgan, T.M., Burnette, W.B., and Markham, L.W. (2012). Correlation of heart rate and cardiac dysfunction in Duchenne muscular dystrophy. Pediatr. Cardiol. *33*, 1175–1179.

55. Kamdar, F., and Garry, D.J. (2016). Dystrophin-Deficient Cardiomyopathy. J. Am. Coll. Cardiol. *67*, 2533–2546.

56. Ben-Ari, M., Schick, R., Barad, L., Novak, A., Ben-Ari, E., Lorber, A., Itskovitz-Eldor, J., Rosen, M.R., Weissman, A., and Binah, O. (2014). From beat rate variability in induced pluripotent stem cell-derived pacemaker cells to heart rate variability in human subjects. Heart Rhythm *11*, 1808–1818.

57. Ovchinnikova, E., Hoes, M., Ustyantsev, K., Bomer, N., de Jong, T.V., van der Mei, H., Berezikov, E., and van der Meer, P. (2018). Modeling Human Cardiac Hypertrophy in Stem Cell-Derived Cardiomyocytes. Stem Cell Rep. *10*, 794–807.

58. Johansson, M., Ulfenborg, B., Andersson, C.X., Heydarkhan-Hagvall, S., Jeppsson, A., Sartipy, P., and Synnergren, J. (2020). Cardiac hypertrophy in a dish: a human stem cell based model. Biol. Open *9*, bio052381.

59. Deckx, S., Johnson, D.M., Rienks, M., Carai, P., Van Deel, E., Van der Velden, J., Sipido, K.R., Heymans, S., and Papageorgiou, A.P. (2019). Extracellular SPARC increases cardiomyocyte contraction during health and disease. PLoS One *14*, e0209534.

60. Herkert, J.C., Verhagen, J.M.A., Yotti, R., Haghighi, A., Phelan, D.G., James, P.A., Brown, N.J., Stutterd, C., Macciocca, I., Leong, K., et al. (2020). Expanding the clinical and genetic spectrum of ALPK3 variants: Phenotypes identified in pediatric cardiomyopathy patients and adults with heterozygous variants. Am. Heart J. *225*, 108–119.

61. Yang, X., Pabon, L., and Murry, C.E. (2014). Engineering adolescence: maturation of human pluripotent stem cell-derived cardiomyocytes. Circ. Res. *114*, 511–523.

62. Tiburcy, M., Hudson, J.E., Balfanz, P., Schlick, S., Meyer, T., Chang Liao, M.L., Levent, E., Raad, F., Zeidler, S., Wingender, E., et al. (2017). Defined Engineered Human Myocardium With Advanced Maturation for Applications in Heart Failure Modeling and Repair. Circulation *135*, 1832–1847.

63. Masubuchi, N., Shidoh, Y., Kondo, S., Takatoh, J., and Hanaoka, K. (2013). Subcellular localization of dystrophin isoforms in cardiomyocytes and phenotypic analysis of dystrophin-deficient mice reveal cardiac myopathy is predominantly caused by a deficiency in full-length dystrophin. Exp. Anim. *62*, 211–217.

64. Yamamoto, T., Awano, H., Zhang, Z., Sakuma, M., Kitaaki, S., Matsumoto, M., Nagai, M., Sato, I., Imanishi, T., Hayashi, N., et al. (2018). Cardiac Dysfunction in Duchenne Muscular Dystrophy Is Less Frequent in Patients With Mutations in the Dystrophin Dp116 Coding Region Than in Other Regions. Circ. Genom. Precis. Med. *11*, e001782.

65. Leibovitz, S., Meshorer, A., Fridman, Y., Wieneke, S., Jockusch, H., Yaffe, D., and Nudel, U. (2002). Exogenous Dp71 is a dominant negative competitor of dystrophin in skeletal muscle. Neuromuscul. Disord. *12*, 836–844.

66. Urasawa, N., Wada, M.R., Machida, N., Yuasa, K., Shimatsu, Y., Wakao, Y., Yuasa, S., Sano, T., Nonaka, I., Nakamura, A., and Takeda, S. (2008). Selective vacuolar degeneration in dystrophin-deficient canine Purkinje fibers despite preservation of dystrophin-associated proteins with overexpression of Dp71. Circulation *117*, 2437–2448.

67. Lim, K.R.Q., Shah, M.N.A., Woo, S., Wilton-Clark, H., Zhabyeyev, P., Wang, F., Maruyama, R., Oudit, G.Y., and Yokota, T. (2021). Natural history of a mouse model overexpressing the Dp71 dystrophin isoform. Int. J. Mol. Sci. *22*, 12617.

68. Capetanaki, Y., Papathanasiou, S., Diokmetzidou, A., Vatsellas, G., and Tsikitis, M. (2015). Desmin related disease: a matter of cell survival failure. Curr. Opin. Cell Biol. *32*, 113–120.

69. Agnetti, G., Herrmann, H., and Cohen, S. (2022). New roles for desmin in the maintenance of muscle homeostasis. FEBS J. *289*, 2755–2770.

70. Milner, D.J., Weitzer, G., Tran, D., Bradley, A., and Capetanaki, Y. (1996). Disruption of muscle architecture and myocardial degeneration in mice lacking desmin. J. Cell Biol. *134*, 1255–1270.

71. Li, Z., Colucci-Guyon, E., Pinçon-Raymond, M., Mericskay, M., Pournin, S., Paulin, D., and Babinet, C. (1996). Cardiovascular lesions and skeletal myopathy in mice lacking desmin. Dev. Biol. *175*, 362–366.

72. Ferry, A., Messéant, J., Parlakian, A., Lemaitre, M., Roy, P., Delacroix, C., Lilienbaum, A., Hovhannisyan, Y., Furling, D., Klein, A., et al. (2020). Desmin prevents muscle wasting, exaggerated weakness and fragility, and fatigue in dystrophic mdx mouse. J. Physiol. *598*, 3667–3689.

73. Wilson, D.G.S., Tinker, A., and Iskratsch, T. (2022). The role of the dystrophin glycoprotein complex in muscle cell mechanotransduction. Commun. Biol. *5*, 1022.

74. van Westering, T.L.E., Betts, C.A., and Wood, M.J.A. (2015). Current understanding of molecular pathology and treatment of cardiomyopathy in duchenne muscular dystrophy. Molecules *20*, 8823–8855.

75. Ather, S., Wang, W., Wang, Q., Li, N., Anderson, M.E., and Wehrens, X.H.T. (2013). Inhibition of CaMKII phosphorylation of RyR2 prevents inducible ventricular arrhythmias in mice with Duchenne muscular dystrophy. Heart Rhythm *10*, 592–599.

76. Wang, Q., Quick, A.P., Cao, S., Reynolds, J., Chiang, D.Y., Beavers, D., Li, N., Wang, G., Rodney, G.G., Anderson, M.E., and Wehrens, X.H.T. (2018). Oxidized CaMKII (Ca(2+)/calmodulin-dependent protein kinase II) is essential for ventricular arrhythmia in a mouse model of Duchenne muscular dystrophy. Circ. Arrhythm. Electrophysiol. *11*, e005682.

77. Shirokova, N., and Niggli, E. (2013). Cardiac phenotype of Duchenne Muscular Dystrophy: insights from cellular studies. J. Mol. Cell. Cardiol. *58*, 217–224.

78. Mattiazzi, A., Mundiña-Weilenmann, C., Guoxiang, C., Vittone, L., and Kranias, E. (2005). Role of phospholamban phosphorylation on Thr17 in cardiac physiological and pathological conditions. Cardiovasc. Res. *68*, 366–375.

79. Lyon, R.C., Zanella, F., Omens, J.H., and Sheikh, F. (2015). Mechanotransduction in cardiac hypertrophy and failure. Circ. Res. *116*, 1462–1476.

80. Jaalouk, D.E., and Lammerding, J. (2009). Mechanotransduction gone awry. Nat. Rev. Mol. Cell Biol. *10*, 63–73.

81. Zois, N.E., Bartels, E.D., Hunter, I., Kousholt, B.S., Olsen, L.H., and Goetze, J.P. (2014). Natriuretic peptides in cardiometabolic regulation and disease. Nat. Rev. Cardiol. *11*, 403–412.

82. Kerkelä, R., Ulvila, J., and Magga, J. (2015). Natriuretic Peptides in the Regulation of Cardiovascular Physiology and Metabolic Events. J. Am. Heart Assoc. *4*, e002423.

83. Hall, E.J., Pal, S., Glennon, M.S., Shridhar, P., Satterfield, S.L., Weber, B., Zhang, Q., Salama, G., Lal, H., and Becker, J.R. (2022). Cardiac natriuretic peptide deficiency sensitizes the heart to stress-induced ventricular arrhythmias via impaired CREB signalling. Cardiovasc. Res. *118*, 2124–2138.

84. Mori, K., Manabe, T., Nii, M., Hayabuchi, Y., Kuroda, Y., and Tatara, K. (2002). Plasma levels of natriuretic peptide and echocardiographic parameters in patients with Duchenne's progressive muscular dystrophy. Pediatr. Cardiol. *23*, 160–166.

85. Demachi, J., Kagaya, Y., Watanabe, J., Sakuma, M., Ikeda, J., Kakuta, Y., Motoyoshi, I., Kohnosu, T., Sakuma, H., Shimazaki, S., et al. (2004). Characteristics of the increase in plasma brain natriuretic peptide level in left ventricular systolic dysfunction, associated with muscular dystrophy in comparison with idiopathic dilated cardiomyopathy. Neuromuscul. Disord. *14*, 732–739.

86. Mohyuddin, T., Jacobs, I.B., and Bahler, R.C. (2007). B-type natriuretic peptide and cardiac dysfunction in Duchenne muscular dystrophy. Int. J. Cardiol. *119*, 389–391.

87. Cai, W., Zhang, J., de Lange, W.J., Gregorich, Z.R., Karp, H., Farrell, E.T., Mitchell, S.D., Tucholski, T., Lin, Z., Biermann, M., et al. (2019). An Unbiased Proteomics Method to Assess the Maturation of Human Pluripotent Stem Cell-Derived Cardiomyocytes. Circ. Res. *125*, 936–953.

88. Tamiyakul, H., Kemter, E., Kösters, M., Ebner, S., Blutke, A., Klymiuk, N., Flenkenthaler, F., Wolf, E., Arnold, G.J., and Fröhlich, T. (2020). Progressive Proteome Changes in the Myocardium of a Pig Model for Duchenne Muscular Dystrophy. iScience *23*, 101516.

89. Jimenez-Vazquez, E.N., Arad, M., Macías, Á., Vera-Pedrosa, M.L., Cruz, F.M., Gutierrez, L.K., Cuttitta, A.J., Monteiro da Rocha, A., Herron, T.J., Ponce-Balbuena, D., et al. (2022). SNTA1 gene rescues ion channel function and is antiarrhythmic in cardiomyocytes derived from induced pluripotent stem cells from muscular dystrophy patients. Elife *11*, e76576.

90. Finsterer, J., and Stöllberger, C. (2003). The heart in human dystrophinopathies. Cardiology *99*, 1–19.

91. Gavillet, B., Rougier, J.S., Domenighetti, A.A., Behar, R., Boixel, C., Ruchat, P., Lehr, H.A., Pedrazzini, T., and Abriel, H. (2006). Cardiac sodium channel Nav1.5 is regulated by a multiprotein complex composed of syntrophins and dystrophin. Circ. Res. *99*, 407–414.

92. Matamoros, M., Pérez-Hernández, M., Guerrero-Serna, G., Amorós, I., Barana, A., Núñez, M., Ponce-Balbuena, D., Sacristán, S., Gómez, R., Tamargo, J., et al. (2016). Nav1.5 N-terminal domain binding to alpha1-syntrophin increases membrane density of human Kir2.1, Kir2.2 and Nav1.5 channels. Cardiovasc. Res. *110*, 279–290.

93. Rubi, L., Koenig, X., Kubista, H., Todt, H., and Hilber, K. (2017). Decreased inward rectifier potassium current IK1 in dystrophin-deficient ventricular cardiomyocytes. Channels *11*, 101–108.

94. Ohkura, M., Sasaki, T., Kobayashi, C., Ikegaya, Y., and Nakai, J. (2012). An improved genetically encoded red fluorescent Ca2+ indicator for detecting optically evoked action potentials. PLoS One *7*, e39933.

95. Takayama, K., Igai, K., Hagihara, Y., Hashimoto, R., Hanawa, M., Sakuma, T., Tachibana, M., Sakurai, F., Yamamoto, T., and Mizuguchi, H. (2017). Highly efficient biallelic genome editing of human ES/iPS cells using a CRISPR/Cas9 or TALEN system. Nucleic Acids Res. *45*, 5198–5207.

96. Concordet, J.P., and Haeussler, M. (2018). CRISPOR: intuitive guide selection for CRISPR/Cas9 genome editing experiments and screens. Nucleic Acids Res. *46*, W242–W245.

97. Zhang, J., Klos, M., Wilson, G.F., Herman, A.M., Lian, X., Raval, K.K., Barron, M.R., Hou, L., Soerens, A.G., Yu, J., et al. (2012). Extracellular matrix promotes highly efficient cardiac differentiation of human pluripotent stem cells: the matrix sandwich method. Circ. Res. *111*, 1125–1136.

98. Tanosaki, S., Akiyama, T., Kanaami, S., Fujita, J., Ko, M.S.H., Fukuda, K., and Tohyama, S. (2022). Purification of cardiomyocytes and neurons derived from human pluripotent stem cells by inhibition of de novo fatty acid synthesis. STAR Protoc. *3*, 101360.

99. Li, R., Yu, C., Li, Y., Lam, T.W., Yiu, S.M., Kristiansen, K., and Wang, J. (2009). SOAP2: an improved ultrafast tool for short read alignment. Bioinformatics *25*, 1966–1967.

100. Langmead, B., and Salzberg, S.L. (2012). Fast gapped-read alignment with Bowtie 2. Nat. Methods *9*, 357–359.

101. Li, B., and Dewey, C.N. (2011). RSEM: accurate transcript quantification from RNA-Seq data with or without a reference genome. BMC Bioinf. *12*, 323.

102. Love, M.I., Huber, W., and Anders, S. (2014). Moderated estimation of fold change and dispersion for RNA-seq data with DESeq2. Genome Biol. *15*, 550.

OMTN, Volume *34*

Supplemental information

Efficacy of exon-skipping therapy for DMD cardiomyopathy with mutations in actin binding domain 1

Naoko Shiba, Xiao Yang, Mitsuto Sato, Shin Kadota, Yota Suzuki, Masahiro Agata, Kohei Nagamine, Masaki Izumi, Yusuke Honda, Tomoya Koganehira, Hideki Kobayashi, Hajime Ichimura, Shinichiro Chuma, Junichi Nakai, Shugo Tohyama, Keiichi Fukuda, Daigo Miyazaki, Akinori Nakamura, and Yuji Shiba

Supplemental Tables

Table S1. List of primers for genomic PCR and RT-PCR

**genomic PCR**	**Forward primer (5'->3')**	**Reverse primer (5'->3')**
P1 (*AAVS1* intron1)	CTCTTCTCTGTTCAGCCCTAAGAATC	
P2 (*AAVS1* intron1)		CATAGCTCAGTCTGGTCTATCTGCC
P3 (Puro)		CGCGCGTGAGGAAGAGTTCTTG
P4 (*R-CaMP1.07*)	ATGGGTTCTCATCATCATCATCATCATGGTAT GG	
DMD exon1-exon11	GATCACTCACTTTCCCCCTACAG	TGAGGCATTCCCATCTTGAATTTAG
DMD exon1-exon 6	GGCAATTACCTTCGGAGAAAAACG	TTACATTTTTGACCTGCCAGTGGA
Off-target sequencing for *MEF2C-AS1*	AGCTAGGATTTTTAGGAGTGAGCAA	TTGAGAGGGAGTGCTATAAACACAA
Off-target sequencing for *CCIN*	GGTGCTTGGTGAAAGGTTATATCTC	GATTGATGGTGTAGTCCTTTGTCTG
Off-target sequencing for *POLR2K*	CTGGGAATTCAGAGGAATGTCTTCA	GGGAAGCAACTTTACCCTTTATTGT
Off-target sequencing for *NRF1*	TTTGTTATCTGTGCTGAATTTGGGA	GTTCCTGACATACTAATCCATGAATCTT
Off-target sequencing for *LINC00661*	AACGATGACTAGGATGATGAGTGAG	AAACCAAGAGTCTAAAGGCACAAAG
Off-target sequencing for *TOM1L1*	TAGTTATTCTTTGTGTGTCGCACTG	TTAAGGTGTCTTCCATCTCCAAACT
Off-target sequencing for *PARP16*	CAATGTAAAAACTGTGGTAGTGGCT	TAGTCTTTGCTGAGTAAAAGGGGAA
Off-target sequencing for *ENHO/RP11-296L22.8*	CAGGGTAGAGCCATAGTTCCATTTA	CTCACCTAGAGCCTGGAATTAGGAT
Off-target sequencing for *KLHL4-ACA64*	TGAATTGGAGCCTGAACAACTTTAC	TTATATTCATATACCTAAATCTTTTCACTTGCC
Off-target sequencing for *VIT*	CTAATGGTGACACTGGAGGATTTGA	GGAAATCTCAAACTCTTTGGTGAGG
Off-target sequencing for *MAS1*	GAAATACATTTGGCCACCAGTAGAG	TGAGCAAAAATATCAGAGTCCTCCA
Off-target sequencing for *SLAIN2*	ATGTACTAGTGCCCAATCATCAGTT	TGGTTAGAGTAGCGCTGTTGATATT
**RT-PCR**	**Forward primer (5'->3')**	**Reverse primer (5'->3')**
DMD exon2 - exon12	CATTCACAAAATGGGTAAATGCACA	AAGCACCTTATGTTGTTGTACTTGG
**qRT-PCR**	**Forward primer (5'->3')**	**Reverse primer (5'->3')**
TNNT2	TTCACCAAAGATCTGCTCCTCGCT	TTATTACTGGTGTGGAGTGGGTGTGG
MYH6	CTCAAGCTCATGGCCACTCT	GCCTCCTTTGCTTTTACCACT
MYH7	ACAAGCTGCAGCTAAAGGTC	TCAAGATGTGGCAAAGCTAC
TNNI1	CTCTTCAGCAAGAGTTTGCG	CAGCTCCACGAGGACTGAAC
TNNI3	CAGTAGGCAGGAAGGCTCAG	CCTCAAGCAGGTGAAGAAGG
MYL7	GTCTTCCTCACGCTCTTTGG	CCACCTCAGCTGGAGAGAAC
MYL2	TTGGGCGAGTGAACGTGAAAA	CCGAACGTAATCAGCCTTCAG
ATP2A2	CATGACAACCCACTGAGAAGAGAA	CGAAGGTCAGATTGGTCTCATATTT
RYR2	CTGCGCCATTCCTATAGTGG	AGTTGAAGACCGGGAGGTG
CASQ2	AAAGACCCACTCTACGTCGC	CAGGAATTCGTAGCCATCTGGA
CACNA1C	CATGCTCACGGTGTTCCA	TCCTACGGCATCATTGACC
SCN5A	GAGCAACTTGTCGGTGCTG	GATTTGGCCAGCTTGAAGAC
KCNJ2	GGTTTGCTTTGGCTCACTCG	GAACATGTCCTGTTGCTGGC
KCNJ4	TAAACTTGGCCCTGCGTCTT	CTTCTTGACGAAGCGGTTGC
KCND3	TGGCTTCGCGGAAGGGTTT	TGGTGACTCCAGCTCTTGGG
DES	CTGAGCAAAGGGGTTCTGAG	ACTTCATGCTGCTGCTGTGT
SPARC	TTCGGCATCAAGCAGAAGGA	GAAACACGAAGGGGAGGGTT
ALPK3	CTGAGGCCATGCAGAAATGC	ATCTTCCAGTCAACCCCTGC
ANKRD1	TCAAGAACTGTGCTGGGAAG	TAGCTATGCGAGAGGTCTTG
NPPA	TCCAACGCAGACCTGATGGA	GGGCACGACCTCATCTTCTA
NPPB	TGGAAACGTCCGGGTTACAG	CTTCCAGACACCTGTGGGAC
GAPDH	ATGGAAATCCCATCACCATCTT	CGCCCCACTTGATTTTGG

Table S2. DMD sgRNA target sequence

name	region	target sequence		guide sequence + **PAM**	
sgRNA1	intron 2	**ccg** tcatcttcggcagattaatt	rev	AATTAATCTGCCGAAGATGA **CGG**	chrX:32,849,933-32,849,955
sgRNA2	intron 7	**ccc** tatggatggagcatactgca	rev	TGCAGTATGCTCCATCCATA **GGG**	chrX:32,809,222-32,809,244
sgRNA3	intron 9	**cct** cgtgaagagctggtttgttt	rev	AAACAAACCAGCTCTTCACG **AGG**	chrX:32,697,747-32,697,769

PAM; protospacer adjacent motif

Table S3. Potential candidate off-target exonic loci with CFD off-target score more than 0.02.

Off-target sequence is shown with mismatch to sgRNA sequence shown in red.

	**Sequence + PAM**	**CFD Score**		**Associated Gene**
sgRNA1	ACTTAATTTGCTGAAAATGA **AGG**	0.3702	chr5:88943055-88943077	*MEF2C-AS1*
	AATCAATCTGCAGAAGGTGA **AGG**	0.0816	chr9:36170969-36170991	*CCIN*
	AATGTATCTCCCGAAGATAA **GGG**	0.0649	chr8:100153416-100153438	*POLR2K*
	AATTATTCTGCCGTGGCTGA **TGG**	0.0437	chr7:129711550-129711572	*NRF1*
sgRNA2	TGGACTATGCTCCATCCCAA **GGG**	0.0214	chr9:34521211-34521233	*ENHO/RP11-296L22.8*
	CGCAGCAGGCTCCATCCATC **CGG**	0.1299	chr19:16026659-16026681	*LINC00661*
	TGAAGGAGGCTCCATCCACA **TGG**	0.1002	chr15:65258380-65258402	*PARP16*
	AGGAGTATTCTCCATCAATA **TGG**	0.1244	chr17:54915804-54915826	*TOM1L1*
sgRNA3	GGCCAAACCAGCTCTGCACG **TGG**	0.1187	chr2:36808504-36808526	*VIT*
	AAATAACCCACCTCTTCATG **GGG**	0.0692	chr6:159908886-159908908	*MAS1*
	ACACAAACCACCTCCTCACC **TGG**	0.0394	chr4:48420247-48420269	*SLAIN2*

Supplemental Videos

Movie S1. Voltage and calcium imaging of monolayered WT hiPSC-CMs on day 50.

A. Voltage imaging

B. Calcium imaging

Supplemental Figures

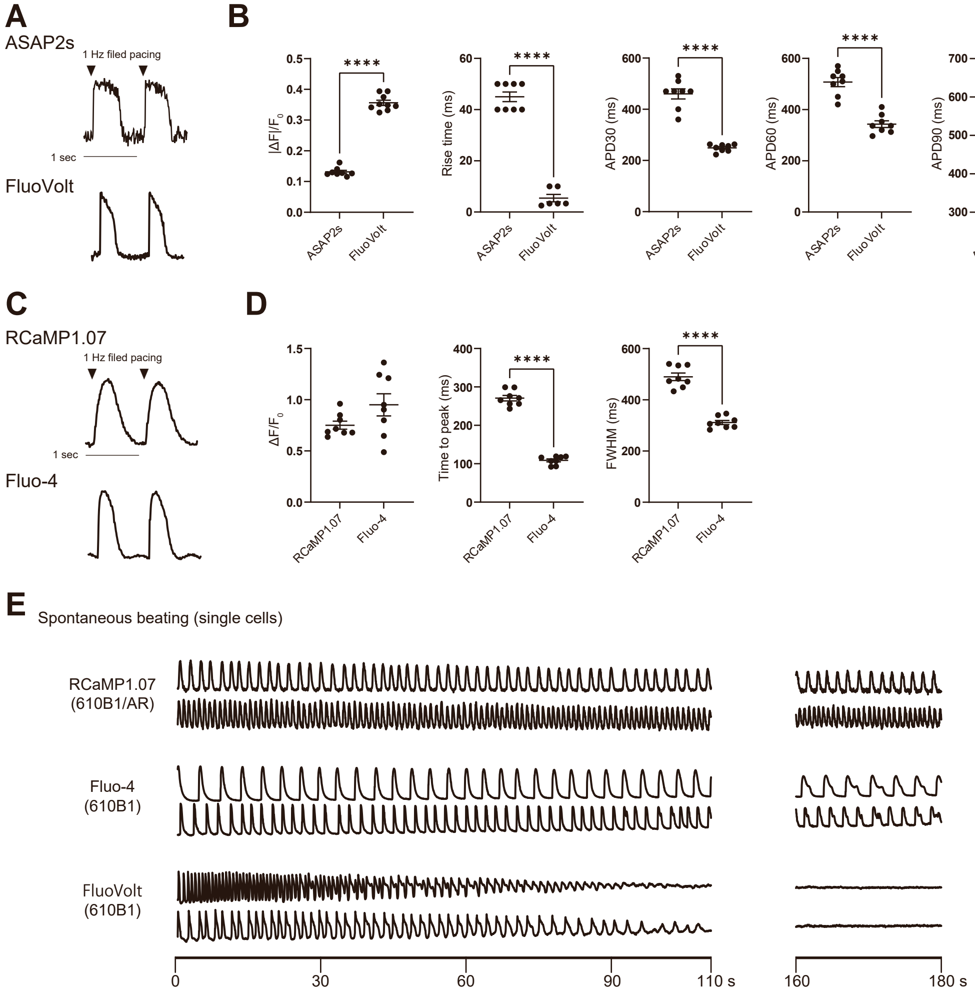

Figure S1. Comparison of live cell imaging waveform between genetically encoded fluorescent ASAP2s/RCaMP1.07 and chemical dyes for voltage and intracellular calcium on day 28 wild-type induced pluripotent stem cell (610B1) derived cardiomyocytes (hiPSC-CMs).
A. Representative traces of ASAP2s and FluoVolt imaging of a single-layered hiPSC-CMs sheet at 1 Hz field pacing. **B.** ΔF/F0, rise time, APD30, APD60, and APD90 (n=8). **C.** Representative traces of RCaMP1.07 and Fluo-4 AM imaging of single-layered hiPSC-CMs sheet at 1 Hz field pacing. **D.** ΔF/F0, Time to peak and full-width at half maximum (FWHM) (n=8). **E.** Representative traces of the single-cell spontaneous beating with RCaMP1.07 on 610B1-ASAP2s/RCaMP1.07 (619B1/AR) cell line and FluoVolt and Fluo-4 on 610B1 cell line for 180-sec traces. In the analysis with FluoVolt and Fluo-4, a decrease in waveform amplitude and the appearance of irregular rhythms were observed after approximately 1 and 2 min, respectively. Abbreviations: fluorescence (F), baseline of fluorescence (F0). Data are presented as mean ± SEM. ****$P < 0.001$.

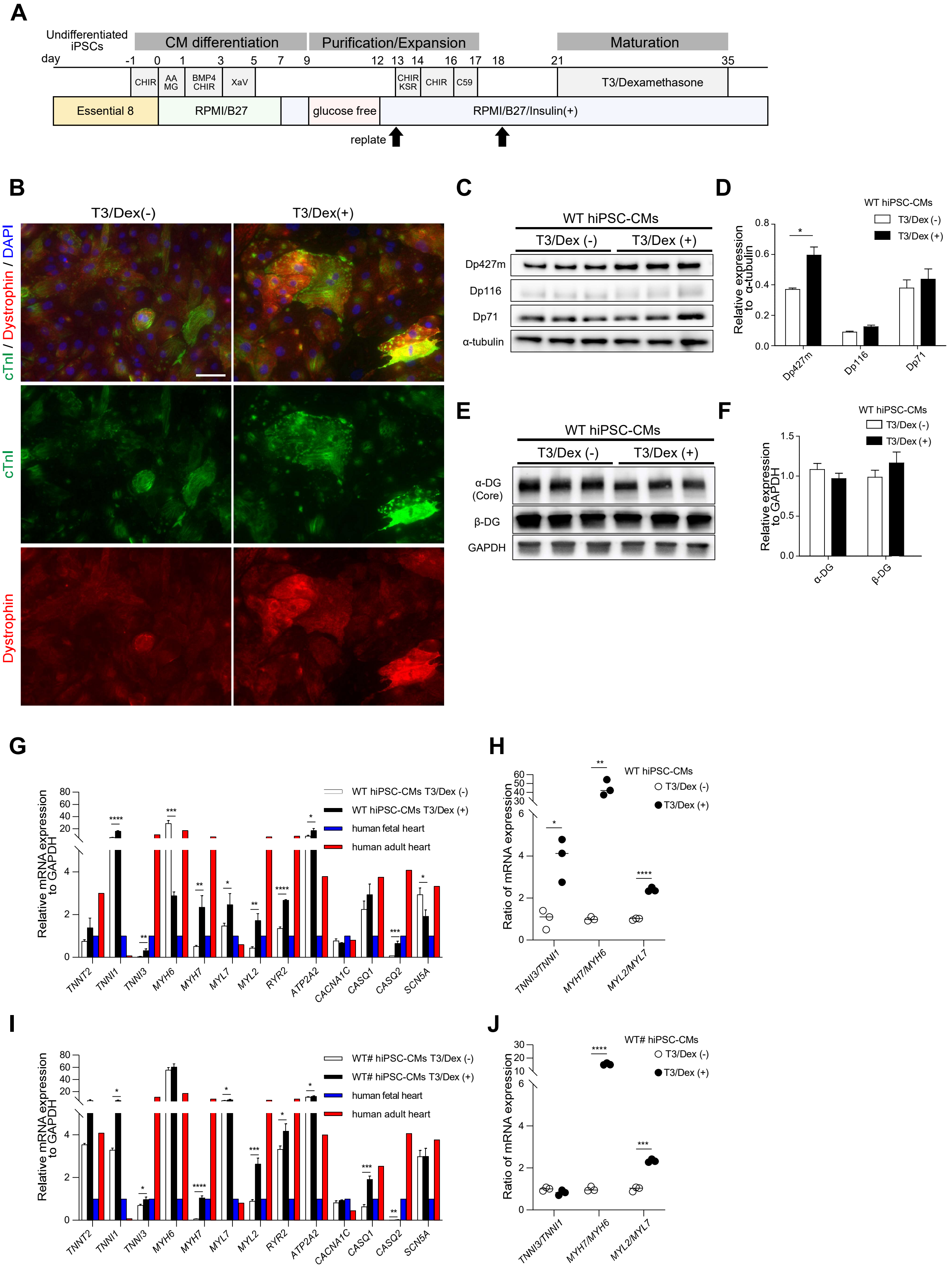
A
Undifferentiated iPSCs
CM differentiation
Purification/Expansion
Maturation
day
-1 0 1 3 5 7 9 12 13 14 16 17 18 21 35
CHIR
AA MG
BMP4 CHIR
XaV
CHIR KSR
CHIR
C59
T3/Dexamethasone
Essential 8
RPMI/B27
glucose free
RPMI/B27/Insulin(+)
replate
B
T3/Dex(-)
T3/Dex(+)
cTnI / Dystrophin / DAPI
cTnI
Dystrophin
C
WT hiPSC-CMs
T3/Dex (-)
T3/Dex (+)
Dp427m
Dp116
Dp71
α-tubulin
D
WT hiPSC-CMs
T3/Dex (-)
T3/Dex (+)
Relative expression to α-tubulin
Dp427m
Dp116
Dp71
E
WT hiPSC-CMs
T3/Dex (-)
T3/Dex (+)
α-DG (Core)
β-DG
GAPDH
F
WT hiPSC-CMs
T3/Dex (-)
T3/Dex (+)
Relative expression to GAPDH
α-DG
β-DG
G
WT hiPSC-CMs T3/Dex (-)
WT hiPSC-CMs T3/Dex (+)
human fetal heart
human adult heart
Relative mRNA expression to GAPDH
TNNT2
TNNI1
TNNI3
MYH6
MYH7
MYL7
MYL2
RYR2
ATP2A2
CACNA1C
CASQ1
CASQ2
SCN5A
H
WT hiPSC-CMs
T3/Dex (-)
T3/Dex (+)
Ratio of mRNA expression
TNNI3/TNNI1
MYH7/MYH6
MYL2/MYL7
I
WT# hiPSC-CMs T3/Dex (-)
WT# hiPSC-CMs T3/Dex (+)
human fetal heart
human adult heart
Relative mRNA expression to GAPDH
TNNT2
TNNI1
TNNI3
MYH6
MYH7
MYL7
MYL2
RYR2
ATP2A2
CACNA1C
CASQ1
CASQ2
SCN5A
J
WT# hiPSC-CMs
T3/Dex (-)
T3/Dex (+)
Ratio of mRNA expression
TNNI3/TNNI1
MYH7/MYH6
MYL2/MYL7

Figure S2. Cardiac differentiation and maturation.
A. Protocol for cardiac differentiation, purification, expansion, and maturation. Abbreviations: AA, activin A; MG, Matrigel; CHIR, CHIR99021; T3, triiodothyronine. **B.** Immunostaining of cardiac troponin I (cTnI) (green) and full-length dystrophin (red) on day 48 isogenic WT control (WT) human induced pluripotent stem cell-derived cardiomyocytes (hiPSC-CMs) with and without T3/Dexamethasone (Dex) treatment. DNA was counterstained with DAPI. Scale bar: 50 µm. **C, D.** Western blot analysis using the anti-dystrophin N-terminus antibody showing protein levels of three isoforms: Dp427, Dp116, and Dp71 in day 48 WT hiPSC-CMs with and without T3/Dex treatment (**C**). Quantification of protein expression in **C** (**D**). **E, F.** Western blot analysis using the anti-human dystroglycan antibody showing protein levels of α-dystroglycan (α-DG (core)) and β-dystroglycan (β-DG) in the same cell lysates as **C** (**E**), and quantification of protein expression in **E** (**F**). **G, I.** Relative mRNA expression of cardiac marker genes to GAPDH in day 48 WT hiPSC-CMs (**G**) and another healthy male-derived non-isogenic control hiPSC-CMs (WT#) (**I**). **H, J.** mRNA ratio of maturation marker genes, TNNI3/TNNI1, MYH7/MYH6, and MYL2/MYL7, in **G** (**H**) and **I** (**J**). Data are presented as mean ± SEM. * $P < 0.05$, ** $P < 0.01$, *** $P < 0.005$, **** $P < 0.001$.

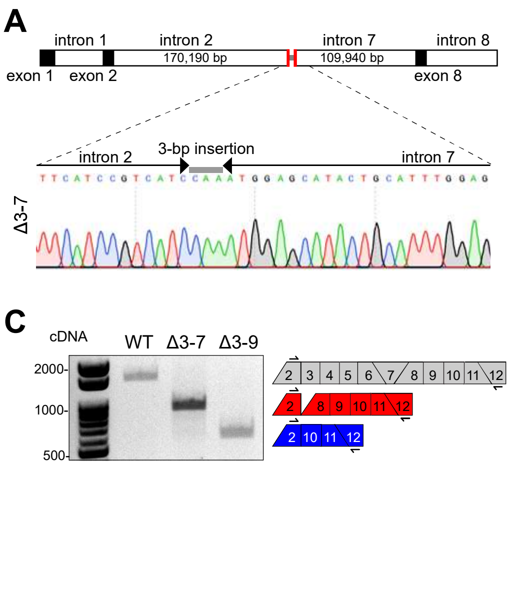

Figure S3. Sequence analysis of isogenic control (WT), Δ3–9, and Δ3–7 human induced pluripotent stem cell-cardiomyocytes (hiPSC-CMs).

A, B. Sanger sequence analyses of genomic DNA extracted from Δ3–7 (**A**) and Δ3–9 (**B**) hiPSC-CMs, including the 5′ side of intron 2 and the 3′ side of the contiguous intron, shows that each cell has a 3-bp and 15-bp insertion in a deep intron close to the protospacer adjacent motif sequences. Red vertical lines indicate cut ends by Cas9. **C.** RT-PCR analysis of CMs differentiated from ASAP2s/R-CaMP1.07-transduced WT, Δ3–7, and Δ3–9 hiPSC-CMs using primers for exons 2 and 12. **D.** Sanger sequence analyses of cDNA from cardiomyocytes differentiated from iCtrl, Δ3–7, and Δ3–9 hiPSC-CMs, including the 3′ side of exon 2 and 5′ side of the subsequent exon, showed that each cell had a correct exon deletion.

A

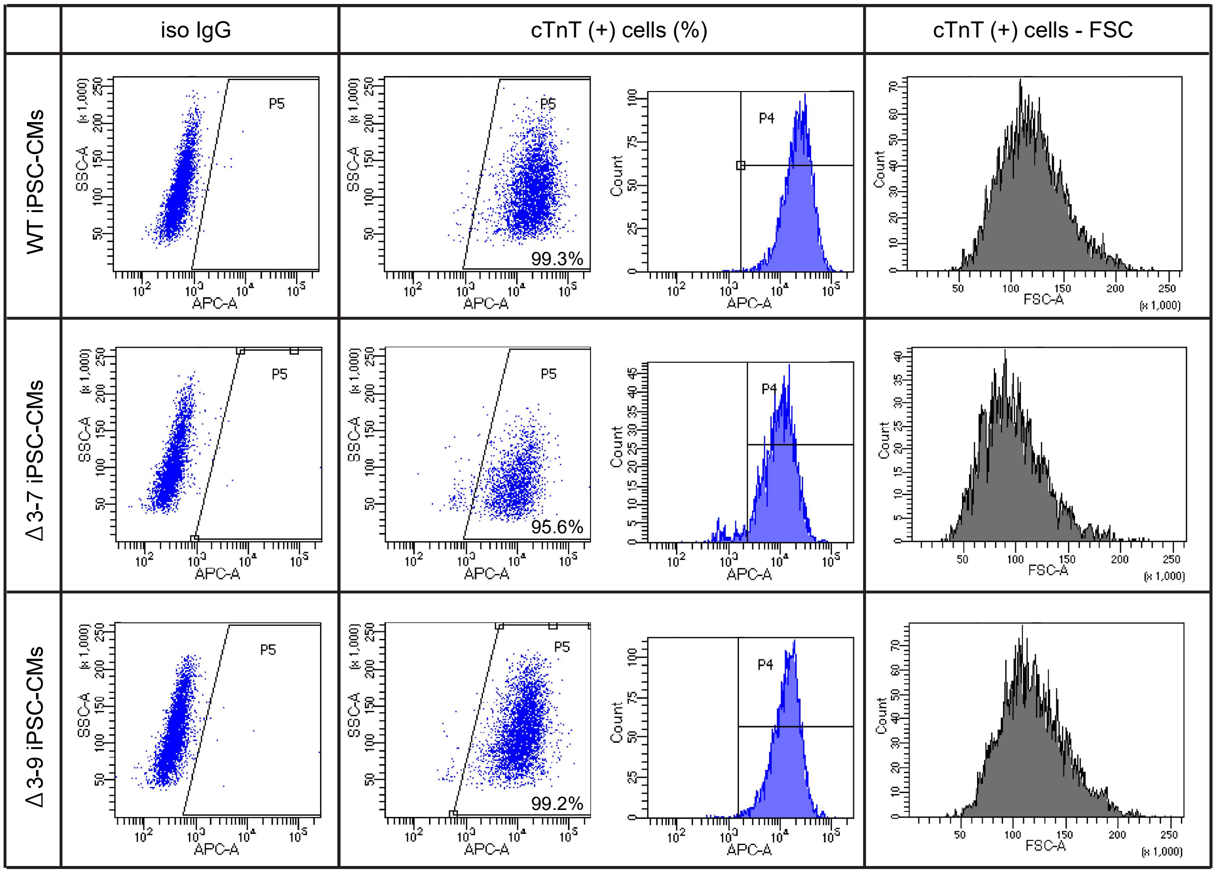

B

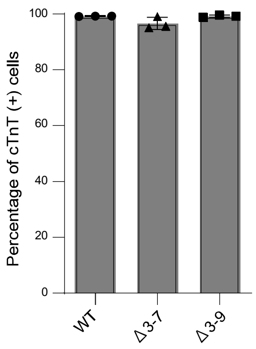

Figure S4. Flow cytometry.
A. Representative flow cytometric analysis at day 48 of human induced pluripotent stem cell-derived cardiomyocytes (hiPSC-CMs) showing the percentage of cardiac troponin T (cTnT) protein expression.
B. Percentage of cTnT positive cells in each cell line, including WT, Δ3-7, and Δ3-9 hiPSC-CMs (n=3).

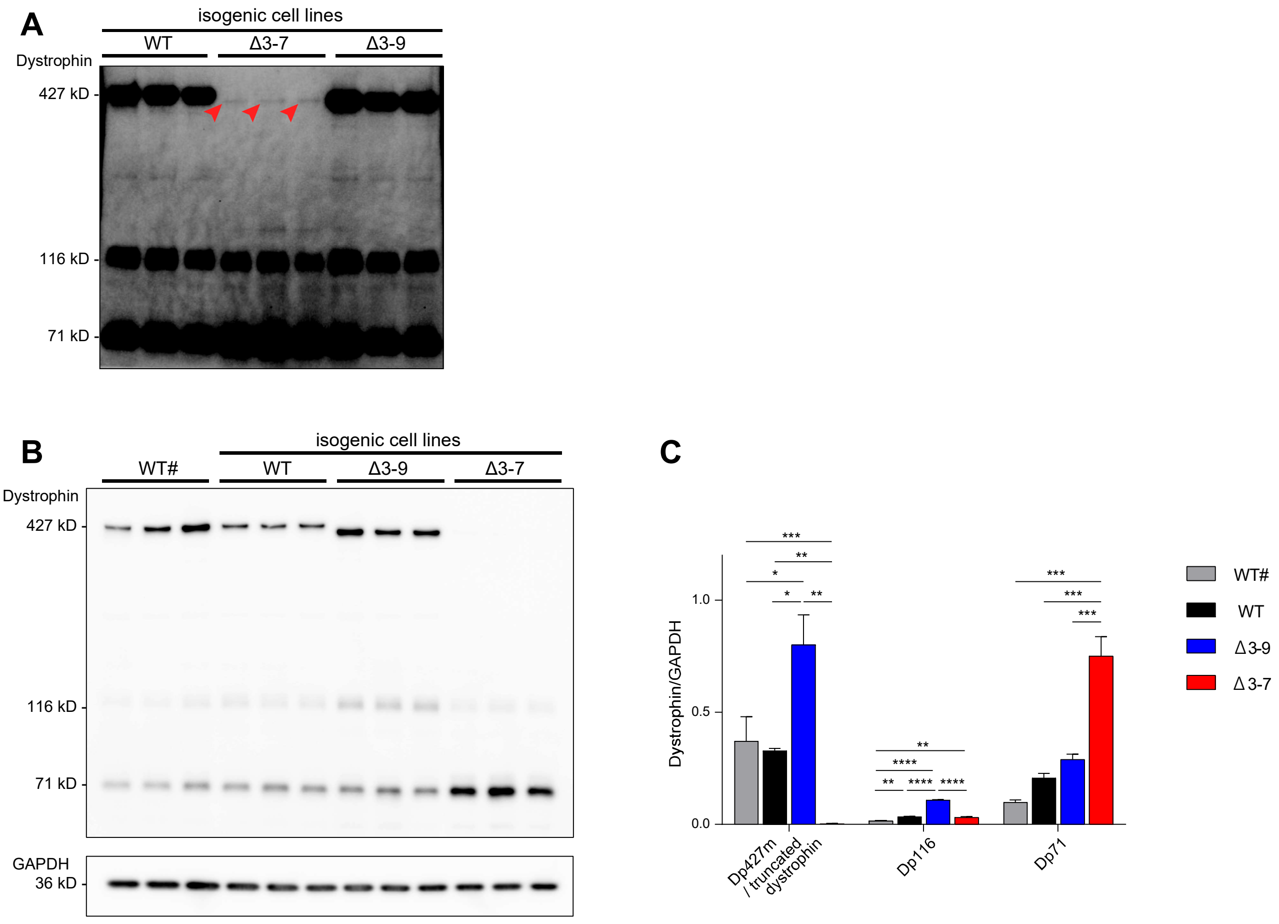

Figure S5. Western blotting analysis.

A. Strongly enhanced membrane picture of dystrophin western blotting using anti-dystrophin N-terminus antibody on Fig. 1F showing faint bands of ~395-kD internally truncated dystrophin on Δ3–7 hiPSC-CMs with the density of approximately 0.5% of WT Dp427m (red arrowhead). B, C. Western blot analysis using the anti-dystrophin N-terminus antibody in day 48 WT hiPSC-CMs, Δ3–7 hiPSC-CMs, Δ3–9 hiPSC-CMs, and another healthy male-derived hiPSC-CMs (WT#) (**B**). Quantification of protein expression in **B** (n=3 independent cardiomyocyte differentiation batches per group) (**C**). Data are presented as mean ± SEM. *$P < 0.05$, **$P < 0.01$, ***$P < 0.005$, ****$P < 0.001$.

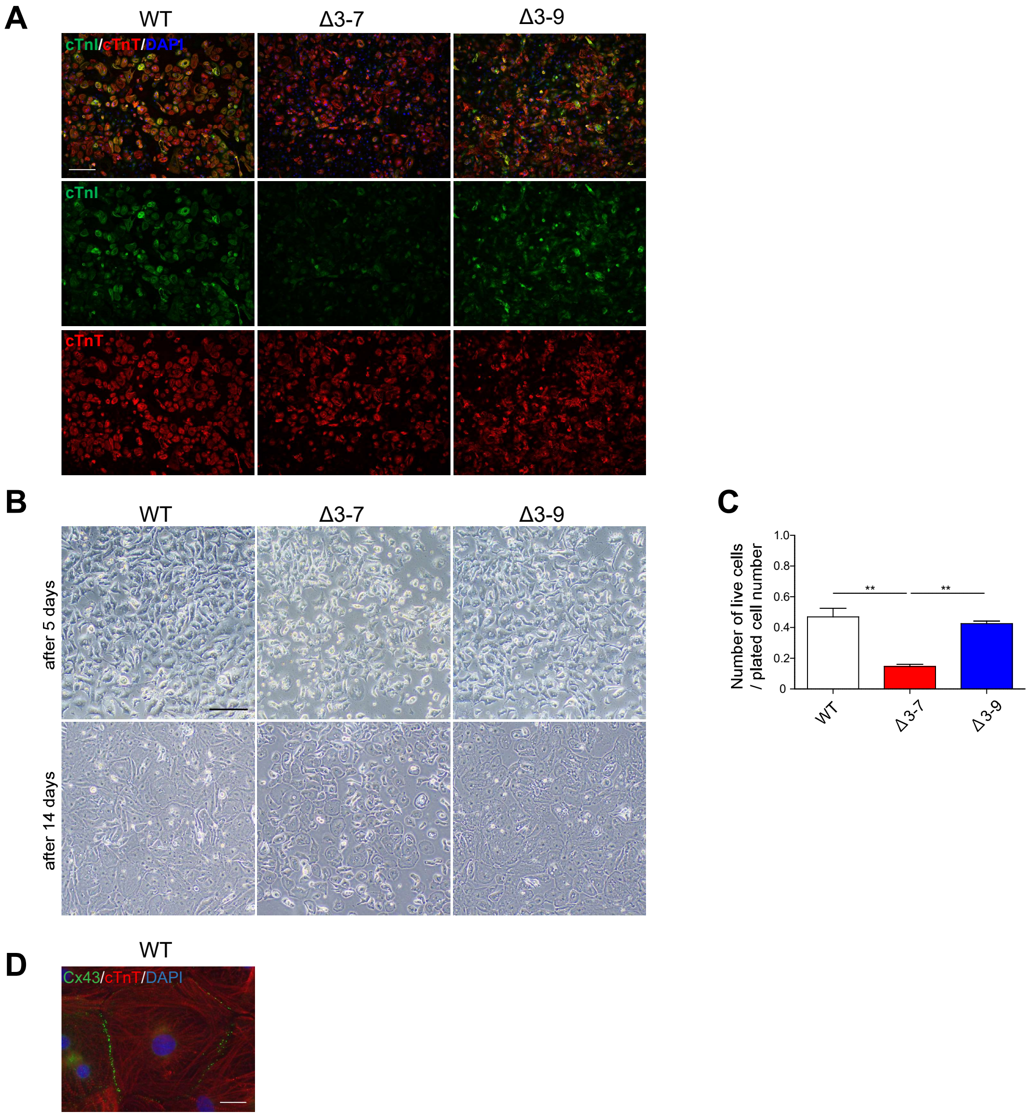

Figure S6. Immunostaining of human induced pluripotent stem cell-cardiomyocytes (hiPSC-CMs).
A. Immunostaining of cardiac troponin I (cTnI) (green) and cTnT (red) on day 48 in WT, Δ3–7, and Δ3–9 hiPSC-CMs. DNA was counterstained with DAPI. Scale bar: 200 μm. **B.** Phase contrast microscopy images of day 35 WT, Δ3–7, and Δ3–9 hiPSC-CMs at 5 d (upper) and 14 d (lower) after replating. Scale bar 500 μm. **C.** Ratio of the number of live cells 14 d after replating to the number of plated cells. **D.** Immunostaining of connexin 43 (Cx43) (green) and cTnT (red) on day 48 in WT hiPSC-CMs. DNA was counterstained with DAPI. Scale bar: 20 μm. Data are presented as mean ± SEM. ***P* < 0.01.

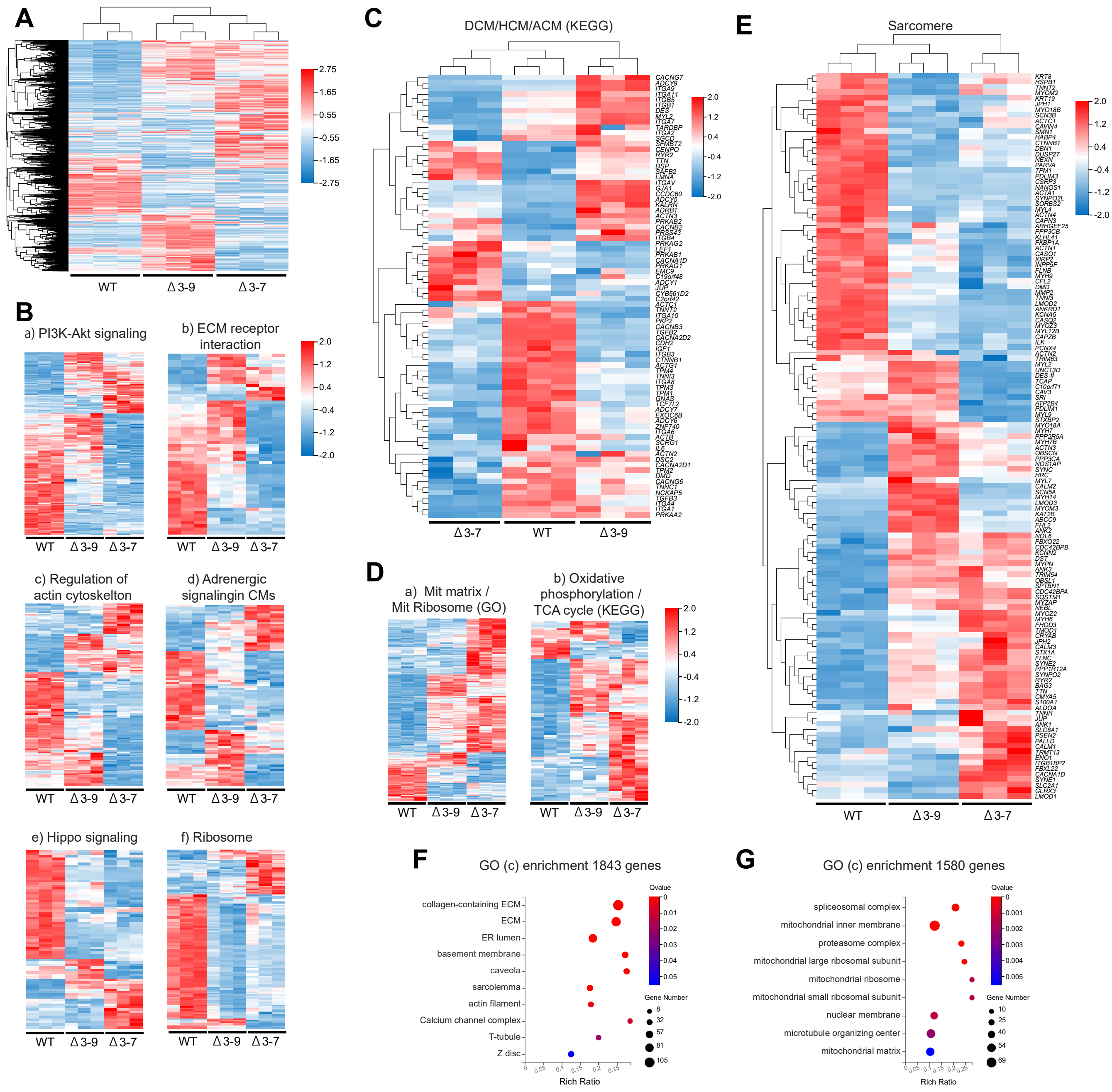

Figure S7. RNA-seq data, related to Figure 5.

A. Hierarchical clustering was performed on 10,000 genes after filtering for low expression levels and coefficients of variation. **B.** Heat map of gene normalized z-scores for log2-transformed FPKM values using the differentially expressed genes (DEGs) involved in major pathways ranked in the Kyoto Encyclopedia of Genes and Genomes pathway enrichment analysis of downregulated genes in Δ3–7 CMs in Fig. 5G, including PI3K-Akt signaling (**a**), ECM receptor interaction (**b**), regulation of actin cytoskeleton (**c**), adrenergic signaling in CMs (**d**), Hippo signaling (**e**), and ribosome (**f**). **C.** Hierarchical clustering of DEGs involved in DCM/HCM/ACM. **D.** Heat map of gene normalized z-scores for log2-transformed FPKM values using the DEGs involved in the mitochondrial matrix and mitochondrial ribosome (**a**) and oxidative phosphorylation and TCA cycle (**b**). **E.** Hierarchical clustering of DEGs involved in the sarcomere, Z-disc, I-band, M-band, and A-band. **F, G.** GO enrichment analysis of the 1843 downregulated (**F**) and 1580 upregulated genes (**G**) in Δ3–7 human induced pluripotent stem cell-cardiomyocytes. Abbreviations: HCM, hypertrophic cardiomyopathy; DCM, dilated cardiomyopathy; ACM, arrhythmogenic cardiomyopathy; CMs, cardiomyocytes; ECM, extracellular matrix.

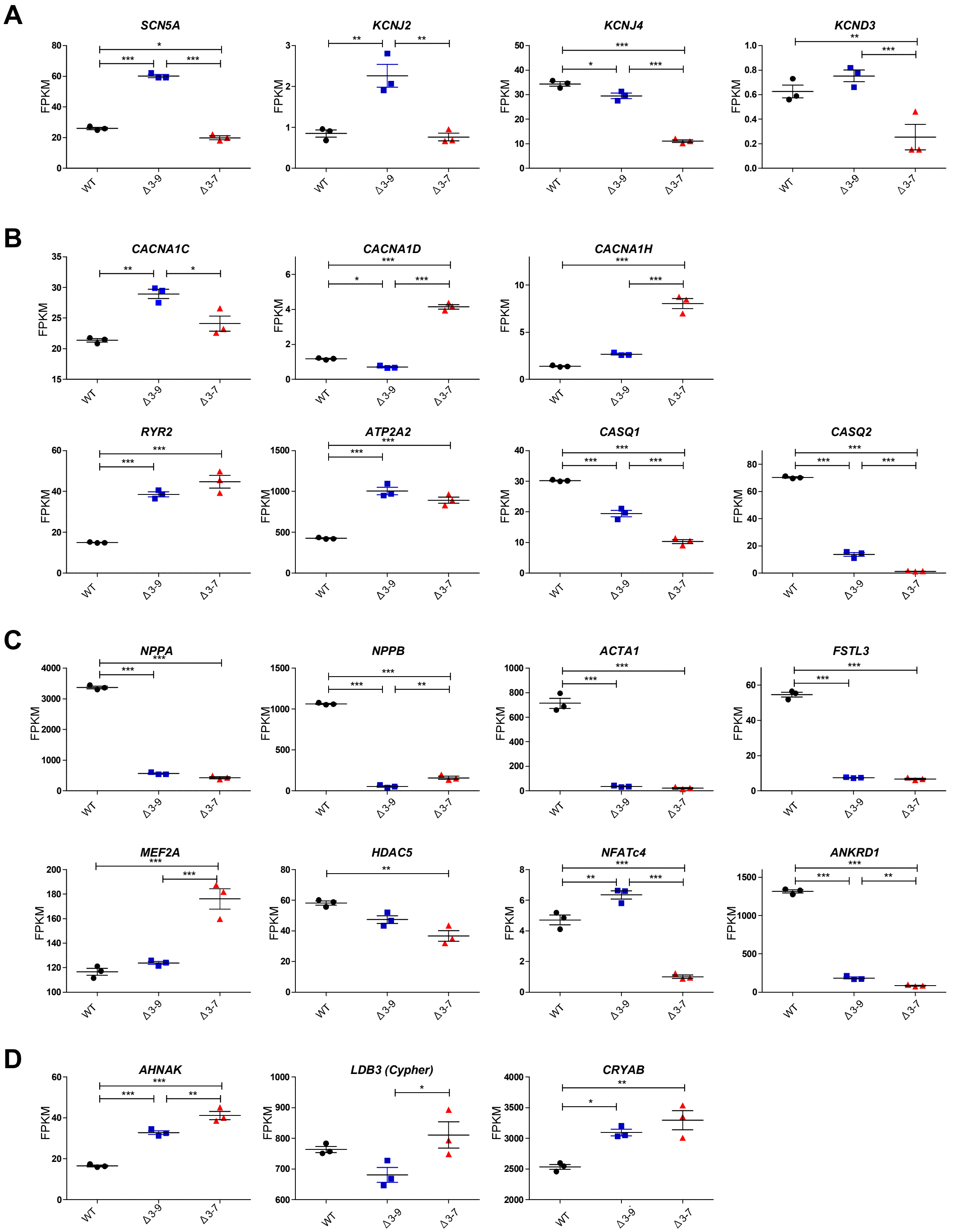

Figure S8. RNA-seq data, related to Figure 5.

A. Action potential associated gene expression (FPKM). **B.** Calcium handling-associated gene expression (FPKM). **C.** Fetal gene and cardiac hypertrophy-related gene expression (FPKM). **D.** Dystrophin-associated cardioprotective gene expression (FPKM). Data are presented as mean ± SEM. **P* < 0.05, ***P* < 0.01, ****P* < 0.005.

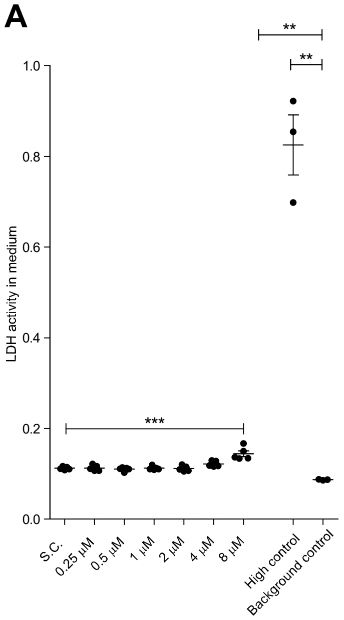

Figure S9. Lactate dehydrogenase (LDH) assay on Δ3–7 human induced pluripotent stem cell-cardiomyocytes (hiPSC-CMs) treated with Vivo-Morpholinos.

A. LDH release level after 12-h incubation with different concentrations of Vivo-Morpholinos (n=5) and Vivo-standard control (S.C.). The high control is the maximum LDH release from the Δ3–7 hiPSC-CMs treated with lysis buffer, and the background control is LDH activity in the culture medium (n=3).

Data are presented as mean ± SEM. $^{**}P < 0.01$, $^{***}P < 0.005$.

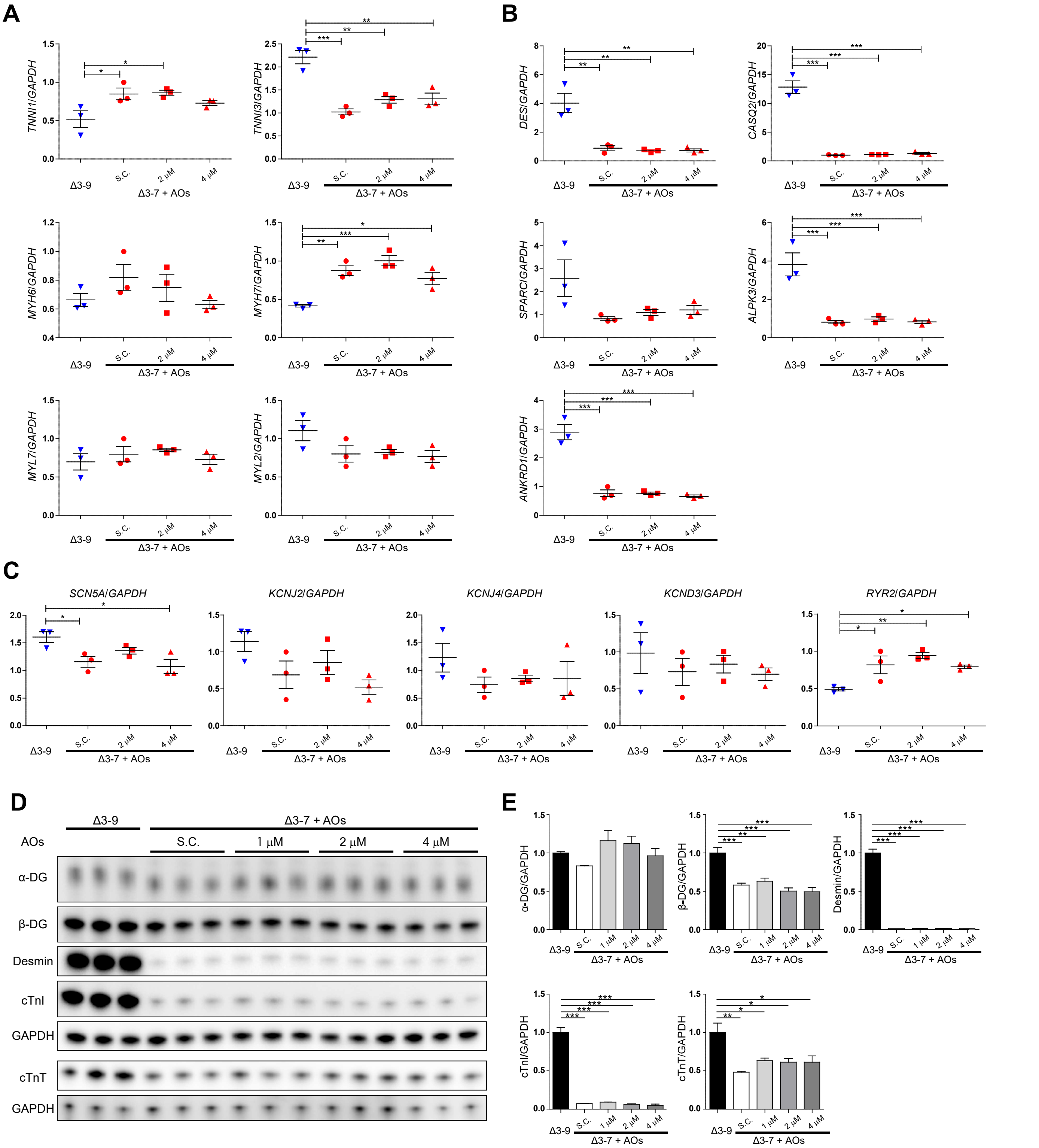

Figure S10. mRNA and protein expression of human induced pluripotent stem cell-cardiomyocytes (hiPSC-CMs) 14 d after exon skipping.

A. RT-PCR analysis of maturation marker genes, including *TNNI3, TNNI1, MYH7, MYH6, MYL2,* and *MYL7*, from untreated Δ3–9 and Δ3–7 hiPSC-CMs 14 d after administration of Vivo-standard control (S.C.) and Vivo-Morpholinos at 1.0–4.0 µM. **B.** RT-PCR analysis of selected genes that were significantly downregulated in day 48 Δ3–7 hiPSC-CMs compared with those in Δ3–9 and wild-type (WT) from untreated Δ3–9 hiPSC-CMs and Δ3–7 hiPSC-CMs 14 d after the administration of S.C. and Vivo-Morpholinos at 1.0–4.0 µM. **C.** RT-PCR analysis of channel genes related to action potential from untreated Δ3–9 hiPSC-CMs and Δ3–7 hiPSC-CMs 14 d after the administration of S.C. and Vivo-Morpholinos at 1.0-4.0 µM. **D.** Western blot analysis showing protein levels of dystrophin-glycoprotein complex composing proteins, including α-DG and β-DG, desmin, cardiac troponin T (cTnT), and cardiac troponin I (cTnI), in Δ 3–9 and Δ3–7 hiPSC-CMs 14 d after administration of S.C. and Vivo-Morpholinos at 1.0–4.0 µM. **E.** Quantification of protein expression in **D**. Data are presented as mean ± SEM. *$P < 0.05$, **$P < 0.01$, ***$P < 0.005$.
